# The clinical impact of conventional therapies for adults and adolescents suffering from eosinophilic esophagitis, a type 2 inflammatory chronic disease, and their economic consequences in Italy: Systematic literature review and meta-analysis

**DOI:** 10.1016/j.jacig.2024.100383

**Published:** 2024-12-11

**Authors:** Giorgio Walter Canonica, Gherardo Mazziotti, Alessandro Repici, Massimiliano Povero, Luca Castello, Lorenzo Pradelli, Miryana Dobreva, Francesca Fanelli, Jean Pierre Saab, Edoardo Vincenzo Savarino

**Affiliations:** a Department of Biomedical Sciences, Humanitas University of Milan, Pieve Emanuele, Milan, Italy; b Personalized Medicine Asthma & Allergy Clinic Humanitas Research Hospital—IRCCS, Rozzano, Milan, Italy; c Endocrinology, Diabetology and Medical Andrology Unit, Metabolic Bone Diseases and Osteoporosis Section, IRCCS Humanitas Research Hospital, Milan, Italy; d Department of Gastroenterology Humanitas University & Humanitas Research Hospital, Rozzano, Milan, Italy; e AdRes SrL, Turin, Italy; f Sanofi Italia, Milan, Italy; g Division of Gastroenterology, Department of Surgery, Oncology and Gastroenterology, University of Padua, Padua, Italy

**Keywords:** Eosinophilic esophagitis, treatment response, economic burden, systematic review, proton pump inhibitors, topical corticosteroids

## Abstract

**Background:**

Eosinophilic esophagitis (EoE) is a chronic inflammatory disorder marked by eosinophilic infiltration of the esophageal mucosa. Despite advances in understanding and management, optimal therapeutic strategies remain unclear, with conflicting guidelines.

**Objective:**

We sought to evaluate effectiveness and safety of topical corticosteroids (TCSs) and proton pump inhibitors (PPIs) in managing EoE and their economic implications in Italy.

**Methods:**

MEDLINE, Embase, and the Cochrane Central Register of Controlled Trials were searched up to December 2023 and 78 publications were included, covering treatment outcomes and adverse events. Meta-analyses were performed to evaluate treatment efficacy and safety across various patient populations and study designs.

**Results:**

TCSs showed superior efficacy over PPIs in achieving histologic, endoscopic, and partial clinical responses. Older patients responded better to both treatments. Treatment outcomes varied by sex and presence of atopic conditions. TCSs discontinuation increased the risk of clinical relapse (0.70 cases per person-year), whereas continuous use was linked to a rise in nonserious adverse events (dilation, infections, upper respiratory tract infections, and skin disorders). Economic analysis indicated cost variations based on treatment regimens and follow-up protocols, with dilation and relapse being significant cost drivers in Italy.

**Conclusions:**

This review provides insights into efficacy, safety, and economic impact of TCSs and PPIs in managing EoE. TCSs were more effective in achieving histologic and endoscopic responses, whereas PPIs were effective in reducing symptoms. Standardized treatment guidelines are needed because of varied treatment efficacy across studies. Future research and new therapies may enhance outcomes and reduce health care costs, improving patient quality of life.

Eosinophilic esophagitis (EoE) is an immune-mediated chronic inflammatory esophageal disease. The primary symptoms include dysphagia and food impaction in adolescents and adults.^1^^,^^2^ Even though EoE is still somewhat new as a condition, its prevalence in Western countries has been steadily increasing in the last 2 decades^3^, ^4^, ^5^ and, as a consequence, it is nowadays recognized as an ordinary disease in clinical practice. EoE has been associated with potential severe clinical consequences, including esophageal stenosis and perforation.^6^ Moreover, EoE has a very negative impact on the health-related quality of life (QoL) of patients because long-term avoidance of the foods that trigger EoE, which is a main pillar in maintaining drug-free disease remission, places a psychological burden that can result in stress and anxiety.^7^

First guidelines for EoE were published in 2007^8^ and then updated in 2011.^9^ On the basis of these guidelines, EoE diagnosis was defined according to the following criteria: (1) clinical symptoms of esophageal dysfunction; (2) 15 or more eosinophil granulocytes per hpf (eos/hpf) on esophageal biopsies; (3) lack of responsiveness to high-dose proton pump inhibitor (PPI) trial (up to 2 mg/kg/d); and (4) exclusion of other causes of esophageal eosinophilia (ie, systemic diseases). Because of this, subsequent diagnostic guidelines defined a new condition termed PPI-responsive esophageal eosinophilia (PPI-REE) for patients with criteria 1 and 2, but improvement or resolution of symptoms and eosinophilia after a high-dose PPI trial.^1^ Starting from 2018, the PPI trial requirement was removed because of similarities between EoE and PPI-REE in terms of clinical, endoscopic, and histologic findings.^10^ Updated versions of the guidelines were published in 2017^7^ and 2024.^6^

Current nonpharmacologic therapeutic options include elemental diet or 2-4-6 food elimination diets (FEDs), but long-term adherence to this approach is difficult to maintain.^11^, ^12^, ^13^ As for the pharmacologic approach, the most widely used pharmacologic agents include PPIs and topical corticosteroids (TCSs), which are adapted from asthma medications that are swallowed rather than inhaled.^14^ However, recently, a novel formulation of budesonide as orally disintegrated tablets (ODTs) has become available in many European countries.^15^ All these formulations of corticosteroids have the theoretical advantage of targeted delivery to the inflammatory site with low systemic absorption. However, a variable amount of TCSs may be absorbed with consequent potential undesired effects on extraintestinal tissues.^16^ As a matter of fact, should TCSs in EoE be notably associated with systemic effects, alternate therapies may increase in appeal. Recently, dupilumab, a fully human mAb that blocks the IL-4 receptor, inhibiting both IL-4 and IL-13 signaling, has been approved by the Food and Drug Administration and the European Medicines Agency for induction and maintenance of remission in patients with EoE aged 12 years or older who are nonresponders to conventional therapies.^17^^,^^18^ On the basis of the results of the phase 3 EoE KIDS study,^19^ dupilumab has recently been approved by the Food and Drug Administration for the pediatric population also. The treatment algorithm of EoE was recently updated with the incorporation of dupilumab in the most recent guidelines on EoE management.^6^ Relapse is common when any treatment for EoE is stopped, including TCSs. In addition, given potential concerns over adverse effects with long-term corticosteroid treatment, additional safety data for this drug class are needed.^15^

The aim of the present study was to review the available evidence to estimate (1) the burden related to EoE in adults and adolescents associated with PPI and TCS treatment in terms of efficacy, safety, QoL, and treatment dynamics and (2) their economic impact for the Italian National Health Service.

## Methods

This systematic literature review (SLR) and meta-analysis (MA) have been designed and conducted to estimate the economic burden related to adult and adolescent patients with EoE treated with PPIs and TCSs. The study protocol has been registered in the International Prospective Register of Systematic Reviews (https://www.crd.york.ac.uk/prospero/; reference no. CRD42024505951) and has been reported according to the PRISMA 2020 checklist.^20^ A brief description of the methods used to perform the review and the MA is given in the following sections.

### Search strategy and study selection

A literature search, designed according to the Patients, Intervention, Comparator, Outcome, Settings criteria,^21^ was conducted on MEDLINE, Embase, and the Cochrane Central Register of Controlled Trials from any date up to December 31, 2023. All terms and strings used for each database are provided in the Online Repository (see Tables E1-E3 in this article’s Additional Material 1 section in the Online Repository at www.jaci-global.org). Two review authors (M.P. and L.C.) independently screened titles and abstracts of all publications according to the eligibility criteria and classified them as *acceptable*, *reconsider*, or *excluded*. Full texts of all articles identified as potentially fulfilling the inclusion/exclusion criteria were retrieved. Conflicting opinions on eligibility and all cases classified as *reconsider* were discussed with a third review author (L.P.), after having consulted the original publication authors for clarification, until consensus was reached.

### Inclusion and exclusion criteria

Randomized clinical trials (RCTs) (*any*), observational prospective, and retrospective studies and case studies (sample size >10 patients per treatment arm) reporting information about efficacy, safety, QoL, and treatment dynamics in adults and adolescent patients (≥12 years) having EoE treated with PPIs and TCS were included. There were no restrictions except for patients’ age and knowledge of the English language. EoE was defined on the basis of at least symptoms and the eos/hpf threshold. Interventions included were PPIs or TCSs in monotherapy or in combination, and the concomitant use of FED as a therapeutic option was also allowed but only if the outcomes were reported for patients with and without FEDs, separately. For observational studies, only those that investigated on-label drugs were considered. For publications for which only the abstract was available, duplicate and nonreliable data (eg, retracted articles) were excluded.

### Data extraction

Two review authors (M.P. and L.C.) independently extracted predefined data from each selected article (ie, classified as acceptable) onto a Microsoft Excel spreadsheet in a standardized collection grid. Data extracted were year of publication, country of origin, analysis period, population characteristics, definition of active EoE, treatments included, duration of therapy, end points of the study (histologic, endoscopic, and symptomatic ones), response duration, number of dilations, and number of patients reporting adverse events, QoL measures, and treatment dynamics. Results of this process were compared and any disagreement was resolved through discussion and in consultation with the principal investigator.

For investigated outcomes shown in graphical format only, numerical values were extrapolated using the Engauge software to digitalize the curves. SE values were transformed into SD with standard formulas. Data reported as median and interquartile range were converted into estimated mean and SD using formulas suggested in the study by Wan et al.^22^

### Outcome assessment

The primary outcomes assessed were histologic response (HR) (≤15 eos/hpf, ≤5-7 eos/hpf, and ≤1 eos/hpf); clinical response (CR), defined as the number of patients with a prespecified reduction in one of the published dysphagia scores (see Table E4 in this article’s Additional Material 1 section in the Online Repository); and endoscopic response (ER), defined as the number of patients with a prespecified reduction in the endoscopic reference score (EREFS).^23^ Variation between values after treatment and at baseline in the Eosinophilic Esophagitis Histology Scoring System,^24^ in total and in each domain of EREFS and in clinical symptoms scores, was also calculated. Moreover, response duration (time to relapse in patients reaching any type of response after PPI or TCS treatment) and any treatment-emergent adverse event (TEAE), including dilations, were also assessed. Secondary outcomes were variation of any QoL score from baseline to follow-up (after PPI or TCS treatment), first-line treatment distribution (at EoE diagnosis), and subsequent treatments after PPI or TCS failure.

### Risk of bias assessment

We based the methodological quality of each study on preselected criteria. The risk of bias for observational studies was assessed through the Newcastle Ottawa Scale questionnaire.^25^ For RCTs, we decided to use the same questionnaire because only active arms were selected and no relative effects (eg, risk ratio of response with TCSs vs placebo) were extracted. In other words, the main advantage of RCT (ie, randomization) did not influence the data extracted and it should not have been evaluated in the bias assessment. The Newcastle Ottawa Scale questionnaire was judged the most suitable instrument to use for all articles because it focused on the aspects that we judged important for our analysis only. For the same reason, 2 of the 8 questions of the questionnaire were excluded (question 2 on selection of the nonexposed cohort and question 1 on comparability between exposed and unexposed) because they could not be evaluated in the included studies (see this article’s Online Repository at www.jaci-global.org). After excluding 2 questions, the total score ranged from 0 (high risk of bias) to 6 (low risk of bias). Articles were classified as “high quality” if the score was greater than or equal to 5 and as “low quality” otherwise.

### Data synthesis and statistical analysis

The MA was performed using the STATA 15 software (StataCorp LLC, College Station, Tex). On the basis of the expected high heterogeneity among studies, all the outcomes (reported in more than 2 studies) were analyzed using a random-effects model. For response outcomes (ie, HR, CR, and ER), the weighting of the studies was performed using the STATA command metaprop and was summarized as mean prevalence (with 95% CI). For continuous outcomes (eg, EREFS), the comparison between baseline and follow-up values was performed using the inverse variance method, and the summary measure of the comparison was the absolute mean difference (AMD). Standardized mean difference (SMD) was exploited if different measurement scales were used (eg, symptoms score). Rate of events (relapse, dilation, and TEAE) was expressed as the number of cases per person-year (PY). A comprehensive set of subgroup analyses was implemented for subgroups with a minimum of 10 studies: studies were stratified according to EoE definition (exclusion vs inclusion of PPI-REE), study characteristics (low quality vs high quality), enrollment countries (Europe, the United States, Asia, and others), TCS type (fluticasone, budesonide, and mometasone furoate) and formulation (ODT vs suspension), and concomitant FED (yes vs no). Furthermore, meta-regression (MR) analysis was conducted to investigate whether high heterogeneity may be further explained by differences in characteristics of the studies. The following variables were taken into consideration when formulating the regression models: age at enrollment; sex (% male); symptoms duration; percentage of each clinical presentation; percentage of each endoscopic *exitus* at baseline; percentage of each comorbidity; baseline EREFS, TCS, or PPI dose; treatment duration; percentage of concomitant PPI (for TCS only); and response assessment (timing). On the basis of the Cochrane guidelines, MR was performed only for outcomes with a minimum number of 10 studies.^26^

### Economic evaluation

Evidences collected in the SLR were used to develop an economic evaluation to estimate the economic burden related to EoE in Italy. A cohort of adults and adolescent patients (≥12 years), which started a first-line treatment with PPI and/or TCS with or without FED, was simulated for 3 years. To estimate the target population, the annual incidence rates of EoE for adults (7 cases per 100,000 residents; 95% CI, 1-18.3) and adolescents (5.1 cases per 100,000 residents; 95% CI, 1.5-10.9)^27^ were applied to the projected Italian population for the next 3 years.^28^ Patients who responded to the treatment at follow-up continued the treatment, whereas nonresponders switched to a subsequent line. During any treatment line, patients could undergo dilation or could experience a relapse according to treatment-specific risks. Patients treated with TCSs could also experience adverse events. All clinical inputs and the treatment distribution in first, second, and subsequent lines were based on the SLR and MA results presented in the next section.

Treatment cost was calculated, for each drug, according to the posology and the least ex-factory price.^29^ The cost of dilation procedure was estimated according to outpatient and inpatient tariffs.^30^^,^^31^ Finally, the cost of each adverse event was estimated on the basis of the specific hospital diagnosis-related group tariff^30^ or published literature^32^ in case of severe events, or by using the cost of €248.25 for an emergency room visit.^33^ Model structure and all input parameters are shown in Figs E1 to E3 and Table E1 (in this article’s Additional Material 6 section in the Online Repository).

## Results

### Literature search results

**Fig 1:**
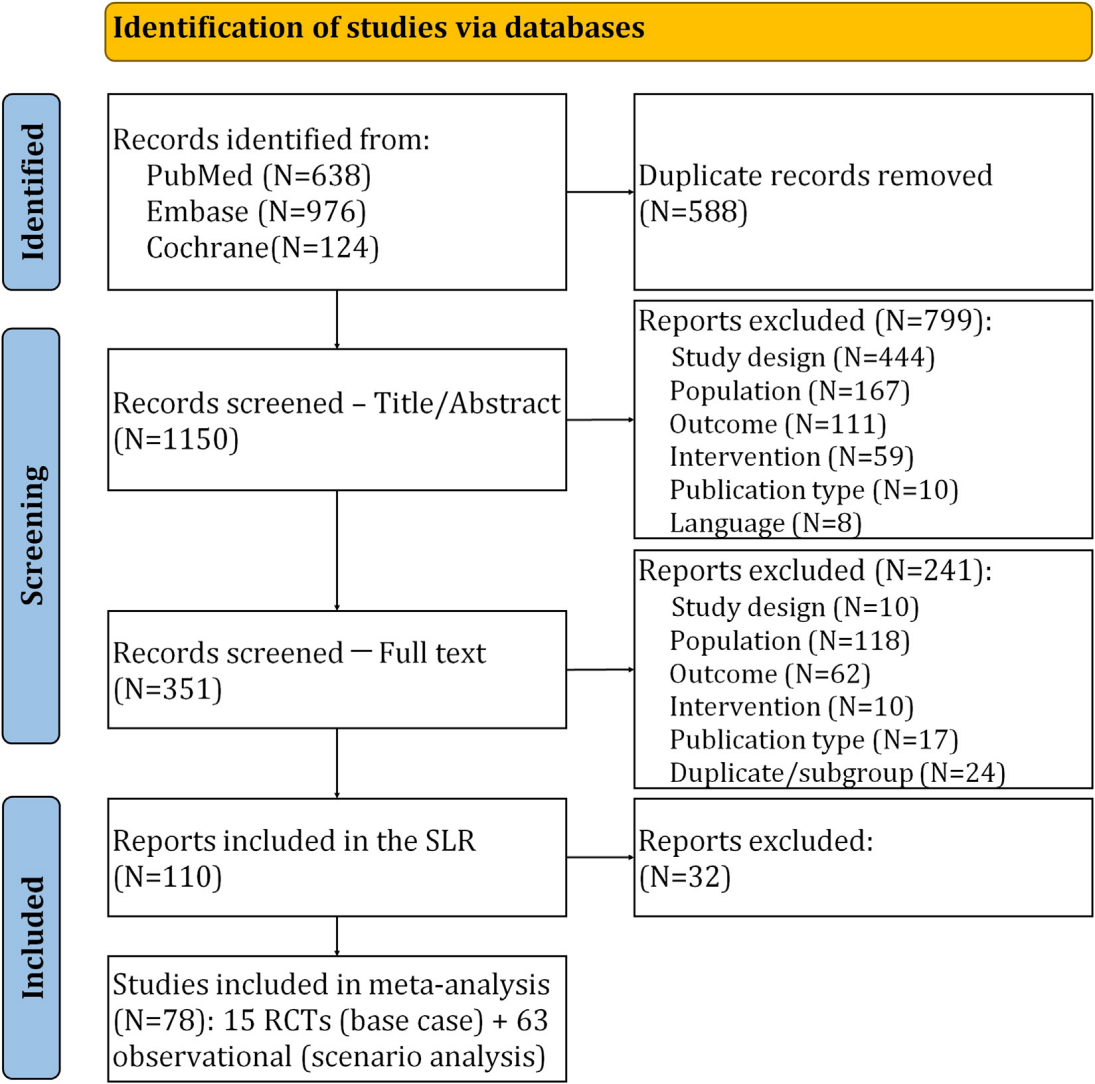
Flowchart of included studies in the MA**.**

Results for each step in the review have been reported according to the PRISMA 2020 checklist.^20^ A total of 1738 articles were selected (Fig 1): 638 from PubMed, 976 from Embase, and 124 from Cochrane. Once the duplicates were removed, 1150 articles were screened by title and abstract and 799 records were excluded (because of wrong study design, mainly). A total of 351 full texts were assessed for eligibility and 241 were excluded (because of wrong population, mainly), and 110 articles were included in the SLR, 78 of which were in the MA (15 RCTs and 63 observational studies).

### Characteristics of included studies

The analysis included all studies from any country published up to December 31, 2023, involving a mean number of patients whose age ranged from 14 to 51 years (mean age, 38 years; 5% adolescents), and on average 71% were male (see Table E1 in this article’s Additional Material 3 section in the Online Repository). At study enrollment, the major clinical symptom was dysphagia (79.2%), the main endoscopic findings were furrows (64.6%) and rings (63.8%), and almost two-thirds of patients had a history of atopic comorbidities (63.9%). A complete description of all clinical symptoms, endoscopic findings, and atopic comorbidities at enrollment is provided in the Online Repository (see Figs E1-E3 in this article’s Additional Material 3 section in the Online Repository).

**Fig 2:**
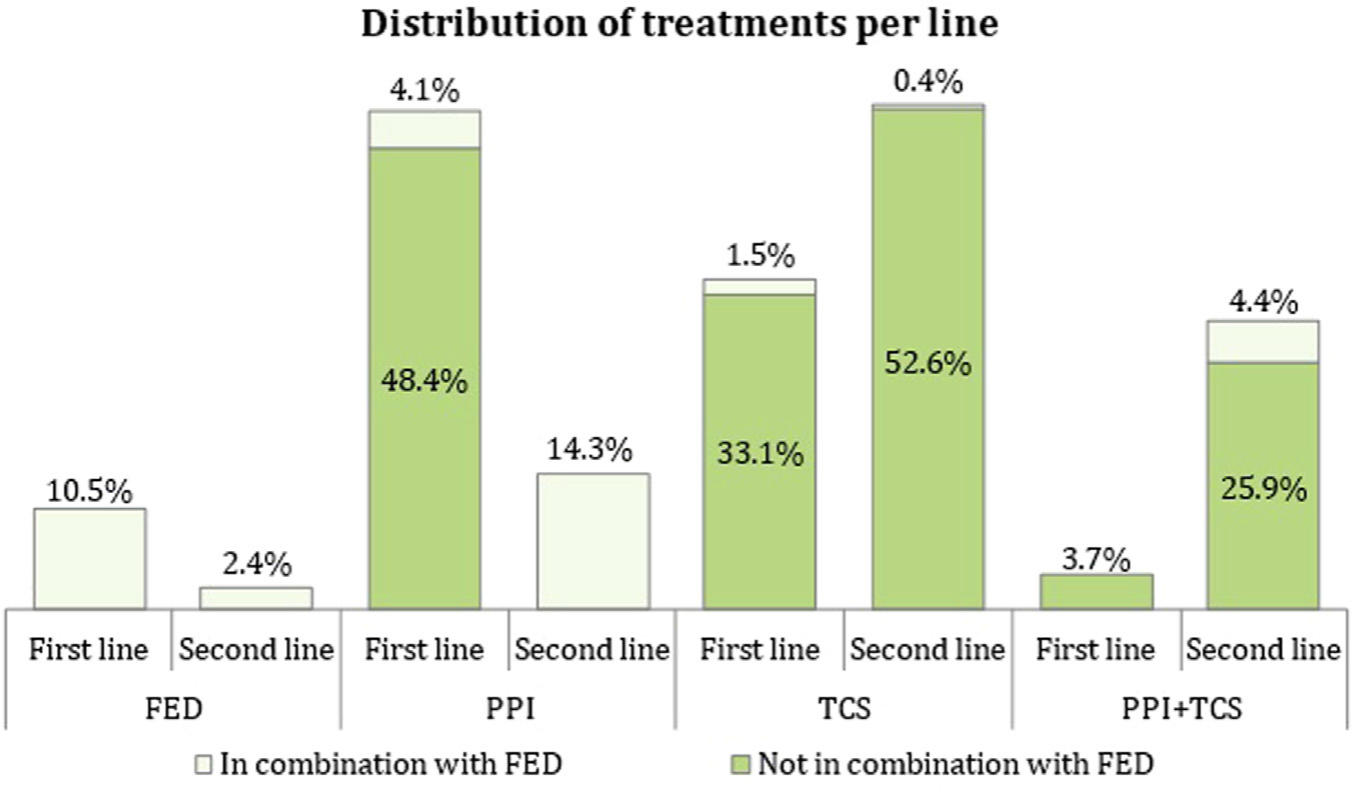
Distribution of treatment in first and second lines (with or without FED).

After EoE diagnosis, about 86% of patients started an active treatment. The most frequent treatment in first line was PPIs, followed by TCSs and FEDs, whereas combination therapy of PPIs and TCSs was prescribed only in 3.7% of patients (Fig 2). Concomitant use of FEDs and PPIs or TCSs was not frequent in first line (8% of total PPIs and 4% of total TCSs). In second line, the most frequent treatment was TCSs alone (52.6%) or in combination with PPIs (25.9%).

In almost all studies, PPIs have been prescribed for 8 weeks and then treatment was continued in case of clinical improvement. However, PPI duration was no longer than 6 months in any study. Regarding TCS treatment, the most prescribed drugs were fluticasone (daily dose of 880 μg) and budesonide (daily dose of 1-2 mg). The treatment was taken continuously, without interruption, and the mean duration was longer than 1 year. Esophagogastroduodenoscopy was performed routinary at the end of treatment for patients treated with PPIs and after a mean of 21.3 weeks for patients treated with TCSs.

Finally, QoL was measured in 13 studies mainly using EoE-QoL-A score (N = 7), and the EuroQol-5D questionnaire was used in only 1 study. Five studies reported an increase in QoL associated with PPI and TCS treatment and 1 study measured a positive significant correlation between endoscopic symptoms reduction and QoL improvement (*ρ* = 0.61; *P* < .001). However, no data could be meta-analyzed because QoL was reported as qualitative results in all articles (see Table E3 in this article’s Additional Material 4 section in the Online Repository).

### Bias assessment

**Fig 3:**
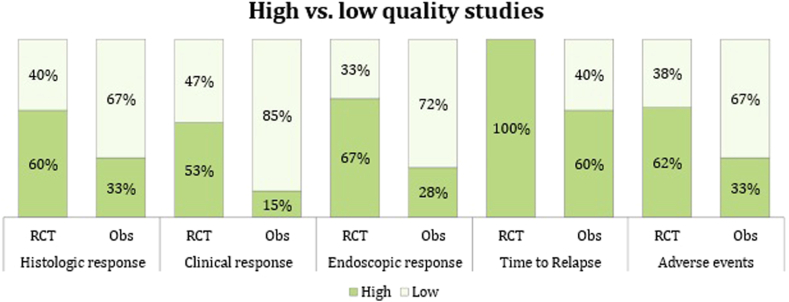
Bias assessment evaluation according to Newcastle Ottawa **S**cale. *Obs*, Observational studies.

According to the Newcastle Ottawa Scale, the quality of the RCTs enrolled in the analysis was overall good (see Fig E4 in this article’s Additional Material 3 section in the Online Repository). There were some concerns about the presence of outcome at the beginning of the observation because some articles were extensions of previous RCTs that investigated the induction phase. The quality of the observational studies was, as expected, lower than that of the RCTs (Fig 3; see also Fig E5 in this article’s Additional Material 3 section in the Online Repository).

### Outcomes

On the basis of the outcomes evaluated, the number of included studies that investigated PPIs were 35 (45%) for HR, 8 (10%) for ER, 8 (10%) for CR, and 2 (3%) for time to relapse, whereas the number of included studies that investigated TCSs were 45 (58%) for HR, 19 (24%) for ER, 31 (40%) for CR, and 8 (10%) for time to relapse. Specifically, time to relapse was evaluated after treatment cessation in 2 studies and after dose reduction in 4 studies; in only 2 studies, patients were treated with the same TCS dose used in induction to achieve the remission. Finally, 32 studies (41%) related to TEAE and dilations with TCSs were also included. For almost all outcomes, the information was based on observational studies mainly (see Fig E8 in this article’s Additional Material 3 section in the Online Repository). Major characteristics of RCTs and observational studies in the MA are provided in Tables E1 and E2 (see this article’s Additional Material 4 section in the Online Repository). Characteristics of excluded studies (ie, those in the SLR, but not in the MA) such as QoL and disease management are provided in Table E3 (see this article’s Additional Material 4 section in the Online Repository). In all the articles included in the MA, TCSs were prescribed alone or in association with PPIs, and no study investigated TCSs in association with FEDs.

#### Efficacy outcomes

Results of MA on efficacy outcomes (based on RCTs only, based on observational studies only, and overall) are presented in Table I, and forest plots for each outcome are presented in the Online Repository (see Figs E1-E23 in this article’s Additional Material 5 section in the Online Repository).

**Table I:** Results of MA for efficacy outcomes

Outcome	RCTs			Observational			HBG	Overall		
	Studies	No. of patients	Estimate (95% CI)	Studies	No. of patients	Estimate (95% CI)	*P* value	Studies	No. of patients	Estimate (95% CI)
HR PPI										
≤15 eos/hpf	1	15	53.3% (26.6 to 78.7)	29	4432	46.0% (37.6 to 54.3)	.59	30	4447	46.2% (37.9 to 54.6)
≤5-7 eos/hpf	2	36	33.3% (17.9 to 48.7)	4	400	37.1% (25.6 to 48.6)	.70	6	436	36.1% (26.7 to 45.4)
≤1 eos/hpf	—	—	NA	2	240	30.3% (21.2 to 39.4)	—	2	240	30.3% (21.2 to 39.4)
HR TCS										
≤15 eos/hpf	8	764	65.2% (53.8 to 76.5)	27	1718	62.6% (55.7 to 69.6)	.71	35	2482	63.4% (57.4 to 69.4)
≤5-7 eos/hpf	14	1332	56.3% (43.6 to 69.1)	10	1078	55.0% (44.2 to 65.8)	.88	24	2410	55.8% (47.0 to 64.6)
≤1 eos/hpf	8	1045	52.9% (37.7 to 68.0)	7	650	32.1% (25.1 to 39.2)	.02	15	1695	45.1% (34.3 to 55.9)
CR PPI (all scores)										
Partial∗	1	15	26.7% (7.8 to 55.1)	3	902	55.6% (25.4 to 85.7)	.13	4	917	48.9% (22.0 to 75.8)
Complete†	—	—	NA	1	53	52.8% (38.6 to 66.7)	—	1	53	52.8% (38.6 to 66.7)
SMD symptoms score‡	2	36	−0.95 (−1.50 to −0.41)	2	140	−2.97 (−3.85 to −2.10)	<.001	4	176	−1.98 (−3.12 to −0.84)
CR TCS (all scores)										
Partial∗	9	826	50.7% (37.5 to 63.9)	8	287	74.4% (63.0 to 85.7)	.008	17	1113	59.5% (48.7 to 70.2)
Complete†	1	21	42.9% (21.8 to 66.0)	2	18	27.9% (8.5 to 47.4)	.31	3	39	34.4% (15.2 to 53.5)
SMD symptoms score‡	12	1205	−0.81 (−1.14 to −0.48)	11	450	−1.43 (−1.88 to −0.97)	.03	23	1655	−1.06 (−1.32 to −0.80)
ER PPI										
Partial§	—	—	NA	3	955	56.1% (0 to 100)	—	3	955	56.1% (0 to 100)
Complete (EREFS = 0)	—	—	NA	2	240	44.0% (37.7 to 50.2)	—	2	240	44.0% (37.7 to 50.2)
AMD EREFS‡	—	—	NA	5	378	−1.72 (−2.09 to −1.34)	—	5	378	−1.72 (−2.09 to −1.34)
ER TCS										
Partial§	1	57	64.0% (51.7 to 76.3)	2	358	63.9% (50.0 to 78.1)	.99	3	415	64.0% (54.4 to 73.6)
Complete (EREFS = 0)	2	317	52.1% (46.6 to 57.6)	—	—	NA	—	2	317	52.1% (46.6 to 57.6)
AMD EREFS‡	9	995	−1.67 (−2.60 to −0.74)	10	940	−1.54 (−2.09 to −1.00)	.81	19	1935	−1.64 (−2.30 to −0.98)
AMD edema	2	180	−0.50 (−0.72 to −0.29)	2	47	−0.45 (−0.72 to −0.19)	.77	4	227	−0.46 (−0.56 to −0.36)
AMD rings	2	180	−0.55 (−0.73 to −0.34)	2	47	−0.54 (−1.19 to 0.12)	.98	4	227	−0.54 (−0.73 to −0.34)
AMD exudates	2	180	−0.87 (−1.43 to −0.27)	2	47	−0.53 (−0.99 to −0.08)	.37	4	227	−0.70 (−1.01 to −0.39)
AMD furrows	2	180	−0.91 (−1.42 to −0.41)	2	47	−0.62 (−1.30 to 0.07)	.50	4	227	−0.77 (−1.11 to −0.43)
AMD strictures	2	180	−0.03 (−0.13 to 0.06)	2	47	−0.10 (−0.17 to −0.02)	.26	4	227	−0.07 (−0.13 to −0.01)
Relapse (event/PY)										
PPI	—	—	—	2	88	0.15 (0.09 to 0.23)	—	2	88	0.15 (0.09 to 0.23)
TCS	4	247	0.29 (0.12 to 0.70)	4	202	0.40 (0.23 to 0.71)	.94	8	449	0.35 (0.22 to 0.54)

*HBG*, Heterogeneity between groups; *NA*, not applicable.

∗ Symptoms reduction.

† Symptoms resolution.

‡ Change from baseline.

§ EREFS ≤ 2 or documented global improvement in EREFS compared with baseline.

For both PPIs and TCSs, the HR was mainly measured according to a threshold less than or equal to 15 eos/hpf (30 studies for PPIs and 35 studies for TCSs). More restrictive thresholds were not frequent in the studies investigating the efficacy of PPIs (6 studies used the ≤5-7 eos/hpf threshold and only 2 studies used the ≤1 eos/hpf threshold), whereas they were much more common in TCS studies (24 and 15 studies, respectively) (Table I). For each threshold, the HR was higher in patients treated with TCSs than in those treated with PPIs (Table I). No significant difference was observed between HR estimated in RCTs and observational studies with the exception of the proportion of patients reaching less than or equal to 1 eos/hpf, with TCSs being significantly higher in randomized studies (52.9% vs 32.1%; *P* = .015).

CR was evaluated in a total of 35 studies (6 on PPIs, 17 on TCSs, and 8 on both). The most frequently validated score used to assess the CR was the Dysphagia Severity Score (23.5%), followed by the Mayo Dysphagia Questionnaire (17.6%), the Dysphagia Symptom Questionnaire and Eosinophilic Esophagitis Activity Index (11.8%), the Watson Dysphagia Scale (8.8%), and the Physician Global Assessment (2.9%). In the remaining 23.5% of included articles (all observational studies), the CR was assessed using a prespecified questionnaire based on severity and frequency of dysphagia symptoms. Even if not validated, such studies were included in the analysis because the scores were objective. On the basis of MA results (Table I), TCSs were associated with higher partial CR than PPIs both in RCTs (50.7% vs 26.7%) and in observational studies (74.4% vs 55.6%); however, the rates observed in RCTs were significantly lower than those observed in observational studies, particularly for TCSs (*P* = .008). The reduction in symptoms score, expressed in terms of SMD, showed the same trend (−0.49 in RCTs vs −1.53 in observational studies; *P* = .042). Conversely, complete CR was higher in PPIs (52.8%) than in TCSs (34.4%); however, the outcome for PPIs was reported just in 1 observational study. Results were confirmed in the scenario analysis by excluding studies assessing CR with nonvalidated scores (Table II).

**Table II:** Results of scenario analysis on CR excluding studies that assessed the response using a nonvalidated score

Outcome	RCTs			Observational			HBG	Overall		
	Studies	No. of patients	Estimate (95% CI)	Studies	No. of patients	Estimate (95% CI)	*P* value	Studies	No. of patients	Estimate (95% CI)
CR PPI										
Partial∗	—	—	NA	2	782	72.4% (69.3 to 75.6)	—	2	782	72.4% (69.3 to 75.6)
Complete†	—	—	NA	1	53	52.8% (38.6 to 66.7)	—	1	53	52.8% (38.6 to 66.7)
SMD symptoms score‡	1	21	−1.21 (−1.87 to −0.55)	—	—	NA	—	1	21	−1.21 (−1.87 to −0.55)
CR TCS (all scores)										
Partial∗	9	826	50.7% (37.5 to 63.9)	8	287	74.4% (63.0 to 85.7)	.008	17	1113	59.5% (48.7 to 70.2)
Complete†	1	21	42.9% (21.8 to 66.0)	2	18	27.9% (8.5 to 47.4)	.31	3	39	34.4% (15.2 to 53.5)
SMD symptoms score‡	12	1205	−0.81 (−1.14 to −0.48)	11	450	−1.43 (−1.88 to −0.97)	.03	23	1655	−1.06 (−1.32 to −0.80)

*HBG*, Heterogeneity between groups.

∗ Symptoms reduction.

† Symptoms resolution.

‡ Change from baseline.

ER was classified in terms of EREFS in all included studies for both PPIs and TCSs. The response was defined as partial if (1) follow-up EREFS was less than or equal to 2 or (2) global improvement was documented in EREFS compared with baseline, whereas response was defined as complete if follow-up EREFS was equal to 0. According to prespecified inclusion/exclusion criteria, no RCT evaluating the ER with PPIs was found. As observed for HR and partial CR, ER (both partial and complete) was also higher in TCSs than in PPIs (partial, 64.0% vs 56.1%; complete, 52.1% vs 44.0%; Table I). The variation of EREFS from baseline to follow-up was expressed in terms of AMD because the same score was used in all articles. The improvement was comparable between PPIs and TCSs (−1.72 vs −1.64) (Table I). Regarding TCSs, no difference was observed between the endoscopic outcomes between RCTs and observational studies (*P* = .990 for partial ER and *P* = .813 for AMD EREFS). Moreover, 4 studies reported the variation in the score for each domain (edema, rings, exudates, furrows, and strings). In each domain, a significant reduction from baseline to follow-up was observed with no difference in the AMDs estimated from RCTs and from observational studies.

Finally, mean relapse rate during TCS treatment was estimated in 0.35 cases per PY (95% CI, 0.22-0.54); the rate was slightly lower in the RCTs (0.29 cases per PY) but the difference was not statistically significant (*P* = .547). The relapse rate was significantly higher when evaluated after treatment cessation (0.70 cases per PY; 95% CI, 0.33-1.50) with respect to dose reduction (0.28 cases per PY; 95% CI, 0.22-0.37) or stable dose, that is, the same dose used to achieve remission in induction (0.20 cases per PY; 95% CI, 0.06-0.59). Details on dose adjustment and relapse consequences for each study are provided in Table E4 (see this article’s Additional Material 4 section in the Online Repository).

The relapse rate with PPIs seemed to be lower (0.15 cases per PY; 95% CI, 0.09-0.23), but the estimate was retrieved just from 2 small observational studies.

#### Safety

Results of MA on safety outcomes (based on RCTs only, based on observational studies only, and overall) are presented in Table III, and forest plots for each outcome are presented in Figs E24 to E51 (in this article’s Additional Material 5 section in the Online Repository).

**Table III:** Adverse event rates estimated from the MA

Adverse events	RCTs			Observational			HBG	Both		
	Studies	No. of patients	Estimate (95% CI)	Studies	No. of patients	Estimate (95% CI)	*P* value	Studies	No. of patients	Estimate (95% CI)
All TEAEs	7	1028	2.92 (1.91-4.45)	1	82	1.34 (0.94-1.89)	.005	8	1110	2.55 (1.73-3.74)
Nonserious	7	1028	2.84 (1.86-4.33)	1	82	1.31 (0.96-1.79)	.004	8	1110	2.48 (1.69-3.64)
Serious	7	1028	0.12 (0.06-0.27)	1	82	0.05 (0.01-0.27)	.34	8	1110	0.11 (0.05-0.22)
Leading to discontinuation	6	740	0.25 (0.11-0.54)	1	82	0.15 (0.05-0.41)	.45	7	822	0.21 (0.11-0.40)
Hospitalizations	2	432	0.03 (0.01-0.10)	—	—	NA	—	2	432	0.03 (0.01-0.10)
Dilation (any time)	4	197	0.20 (0.02-1.93)	10	418	0.33 (0.15-0.70)	.69	14	615	0.29 (0.14-0.57)
Dilation (on treatment)	4	197	0.20 (0.02-1.93)	7	263	0.37 (0.14-0.64)	.61	11	460	0.30 (0.14-0.64)
Infection and infestation	2	232	1.10 (0.80-1.53)	1	82	0.90 (0.64-1.26)	.40	3	314	1.00 (0.79-1.26)
Oral candidiasis	7	509	0.22 (0.13-0.36)	5	362	0.02 (0.01-0.04)	<.001	12	871	0.12 (0.05-0.27)
Esophageal candidiasis	10	923	1.02 (0.50-2.08)	8	396	0.05 (0.02-0.11)	<.001	18	1319	0.32 (0.15-0.69)
UTI	4	499	0.50 (0.28-0.88)	3	155	0.16 (0.04-0.64)	.14	7	654	0.32 (0.16-0.64)
Sinusitis	3	483	0.19 (0.12-0.29)	1	82	0.05 (0.01-0.21)	.01	4	565	0.17 (0.11-0.25)
GI disorders	2	232	0.51 (0.17-1.56)	1	82	0.22 (0.11-0.45)	.21	3	314	0.34 (0.15-0.78)
Diarrhea	3	286	0.45 (0.08-2.40)	1	82	0.08 (0.03-0.25)	.09	4	368	0.25 (0.07-0.85)
Nausea/vomiting	3	502	0.35 (0.14-0.85)	—	—	NA	—	3	502	0.35 (0.14-0.85)
Abdominal pain/discomfort	2	289	0.23 (0.04-1.22)	1	82	0.05 (0.01-0.21)	.19	3	371	0.13 (0.05-0.38)
Respiratory disorders	2	232	0.46 (0.28-0.76)	1	82	0.28 (0.15-0.51)	.22	3	314	0.38 (0.25-0.56)
Oropharyngeal pain	3	199	0.25 (0.07-0.92)	1	82	0.05 (0.01-0.25)	.13	4	281	0.16 (0.05-0.47)
Cough	3	483	0.13 (0.08-0.21)	—	—	NA	—	3	483	0.13 (0.08-0.21)
Dyspnea	1	51	0.08 (0.01-0.60)	1	82	0.05 (0.01-0.27)	.73	2	133	0.06 (0.02-0.22)
Skin disorders	2	232	0.13 (0.03-0.66)	1	82	0.17 (0.08-0.39)	.76	3	314	0.17 (0.09-0.32)
Acne	2	264	0.10 (0.04-0.22)	1	82	0.10 (0.03-0.34)	.99	3	346	0.10 (0.05-0.19)
Contact dermatitis/hives	2	108	0.63 (0.15-2.63)	2	110	0.10 (0.03-0.36)	.06	4	218	0.29 (0.09-0.91)
Others	8	828	0.44 (0.25-0.78)	4	209	0.06 (0.02-0.19)	.002	12	1037	0.24 (0.12-0.46)
Bolus impaction	—	—	NA	1	82	0.02 (0.01-0.05)	—	1	82	0.02 (0.01-0.05)
Chest pain	1	129	0.05 (0.01-0.36)	1	82	0.05 (0.01-0.27)	.99	2	211	0.05 (0.02-0.18)
BCD	4	648	0.40 (0.15-1.07)	—	—	NA	—	4	648	0.40 (0.15-1.07)
Fever	1	51	0.08 (0.01-0.60)	1	82	0.08 (0.03-0.25)	.99	2	133	0.08 (0.03-0.22)
Fatigue	2	270	0.13 (0.07-0.26)	1	82	0.05 (0.01-0.25)	.28	3	352	0.11 (0.06-0.21)
Dysgeusia	1	181	0.10 (0.02-0.38)	—	—	NA	—	1	181	0.10 (0.02-0.38)
Psychiatric	3	613	0.11 (0.06-0.21)	—	—	NA	—	3	613	0.11 (0.06-0.21)

Data are presented as number of events per PY and 95% CI.

*BCD*, Blood cortisol decrease; *GI*, gastrointestinal; *NA*, not applicable; *UTI*, upper tract infection.

Annual rate of adverse events was 2.55 cases per PY (95% CI, 1.73-3.74). The estimate was significantly higher in the RCTs than in the observational studies (2.92 vs 1.34; *P* = .005). However, in both RCTs and observational studies, almost all adverse events were nonserious (2.84 and 1.31 cases per PY, respectively). Consequently, the rate of adverse events leading to discontinuation was low (0.21 cases per PY; 95% CI, 0.11-0.40), with no significant difference among RCTs and observational studies (*P* = .448). Similarly, adverse events leading to hospitalization were rare because they were reported in only 2 studies and the overall rate was very low (0.03 cases per PY; 95% CI, 0.01-0.10).

The most common adverse events reported in the RCTs were (1) infections (1.10 cases per PY; 95% CI, 0.80-1.53), mainly esophageal candidiasis or upper tract infection (1.02 and 0.50 cases per PY, respectively); (2) contact dermatitis or hives (0.63 cases per PY; 95% CI, 0.15-2.63); (3) gastrointestinal disorders (0.51 cases per PY; 95% CI, 0.17-1.56); (4) respiratory disorders (0.46 cases per PY; 95% CI, 0.28-0.76); and (5) blood cortisol decrease (0.40 cases per PY; 95% CI, 0.15-1.07). The severity of all events of blood cortisol decrease was mild to moderate and the events resolved after treatment discontinuation without clinical consequences.

A similar trend was observed in the observational studies but the adverse event rates were systematically lower than those observed in the RCTs.

Finally, the rate of dilation was moderate both during treatment (0.30 cases per PY; 95% CI, 0.14-0.66) and for longer follow-up (0.29 cases per PY; 95% CI, 0.14-0.58), with no significant difference between RCTs and observational studies.

An exploratory analysis on the association between the proportion of patients experiencing adverse events and the duration of follow-up is provided in Table E1 (see this article’s Additional Material 5 section in the Online Repository). Studies were stratified on the basis of short-term follow-up (<12 weeks), mid-term follow-up (12-26 weeks), and long-term follow-up (>26 weeks). Most of the adverse events were more frequent in mid- to long-term follow-up studies; proportion of patients experiencing any TEAEs was 57.6% in short-term follow-up studies, 61.0% in mid-term follow-up studies, and 73.1% in long-term follow-up studies (*P* < .001). The same trend was observed in the proportion of patients experiencing dilation, any infection and infestation, upper respiratory tract infection, and skin disorders. Conversely, esophageal candidiasis was more frequent in short-term follow-up studies.

#### Subgroup analysis and MR

Subgroup analyses are provided in Table E2 (see this article’s Additional Material 5 section in the Online Repository) for the outcomes reported in more than 10 studies, according to the prespecified variables: inclusion versus exclusion of PPI-REE, study quality (low vs high), enrollment countries, TCS type, and formulation. The effect of concomitant FED has not been investigated because none of the included studies investigated PPIs or TCSs in association with FED. Conversely, an additional subgroup analysis based on sample size lower versus higher than 50 patients was performed to assess the impact of small studies on the MA results.

Efficacy was higher, on average, in the studies in which EoE diagnosis was made according to the A working Group on PPI-Responsive Esophageal Eosinophilia (AGREE) Guidelines,^10^ that is, those studies that did not exclude PPI-REE from the enrollment. Specifically, HR with PPIs and SMD in symptoms score with TCSs were significantly higher in this subgroup. Efficacy was also higher in studies conducted in Europe with respect to those conducted in the United States; conversely, the dilation rate was significantly lower.

No differences were observed between patients treated with budesonide or fluticasone in almost all outcomes with the exception of HR on the basis of the threshold of less than or equal to 1 eos/hpf (*P* < .001); however, the presence of studies that did not report results for budesonide and fluticasone separately could bias this subgroup analysis. Conversely, the ODT TCSs, compared with oral suspension, were associated with higher HR and lower dilation rate (*P* < .001), but higher incidence of esophageal candidiasis (*P* = .005).

Results from high-quality studies agreed with those estimated from low-quality studies, with the exception of the reduction in EREFS from baseline in patients treated with TCSs, which resulted statistically higher in high-quality studies (−2.45 vs −0.62; *P* < .001). Finally, studies that enrolled fewer than 50 patients were more likely to underestimate the HR to TCSs, whereas no differences between small and big studies were observed in the other outcomes.

MR results are provided in Table E3 (see this article’s Additional Material 5 section in the Online Repository) for the outcomes and covariates reported in more than 10 studies. Age at enrollment seemed to explain about 13% to 23% of the total heterogeneity (ie, variability) observed in the HR among selected studies, with older patients associated with better response. Sex seemed to explain about 12% to 26% of the variability observed in more restrictive HR definitions (≤5-7 and ≤1 eos/hpf), dilation rate, and adverse events. Endoscopic characteristics at enrollment explained from 16% (baseline EREFS) to 55% (prevalence of exudates) of the heterogeneity observed in the variation in the EREFS from baseline after TCS treatment. Atopic comorbidities explained almost 50% of the variability in HR (≤15 eos/hpf) both to PPIs and TCSs and variation in symptoms score from baseline, prevalence of food allergy especially. Finally, the mean dose of TCS explained almost 20% of the heterogeneity in the partial CR, 25% to 30% of the heterogeneity in the dilation rate, and 11% of the heterogeneity in the incidence of oral candidiasis.

#### Economic burden of EoE

**Fig 4:**
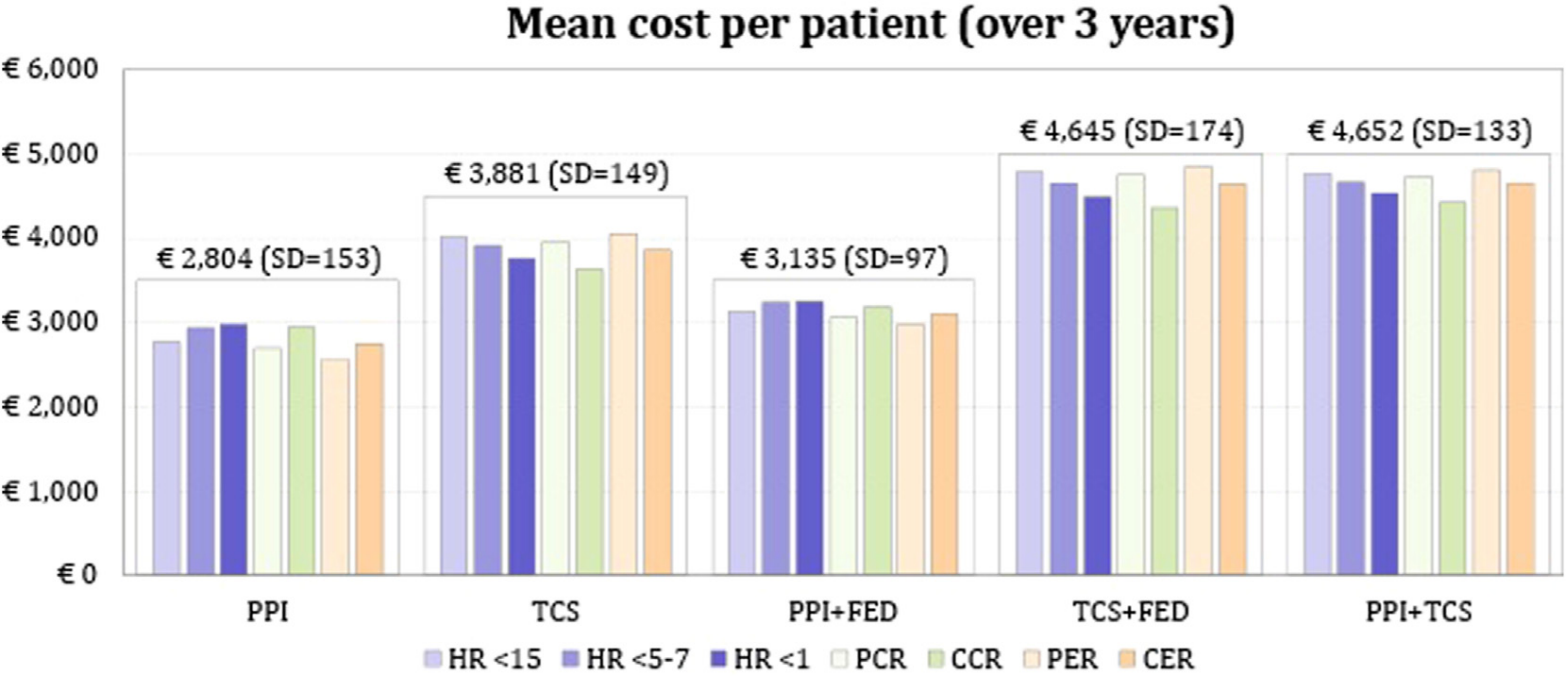
Mean cost per patient according to criteria used to assess the response at follow-up. *CCR*, Complete clinical response; *CER*, complete endoscopic response; *PCR*, partial clinical response; *PER*, partial endoscopic response.

On the basis of the annual incidence of EoE, 8546 new patients with EoE were expected to start a treatment in the next 3 years (2849 every year) in Italy. The mean cost per patient over the 3 years of analysis, according to the treatment started in first line, is shown in Fig 4. The mean cost is slightly affected by the criteria used to assess the response at follow-up (HR, partial or complete CR, and partial or complete ER). Over the 3 years of analysis, the mean cost of patients starting PPIs as first line was €2804, followed by PPI + FED (€3135), TCSs (€3881), PPI + TCS (€4652), and TCS + FED (€4645). The main drivers are the cost of dilation and relapse, followed by the cost of treatment and adverse event management. Detailed costs according to each response criterion are provided in Tables E2 to E8 (see this article’s Additional Material 6 section in the Online Repository).

**Fig 5:**
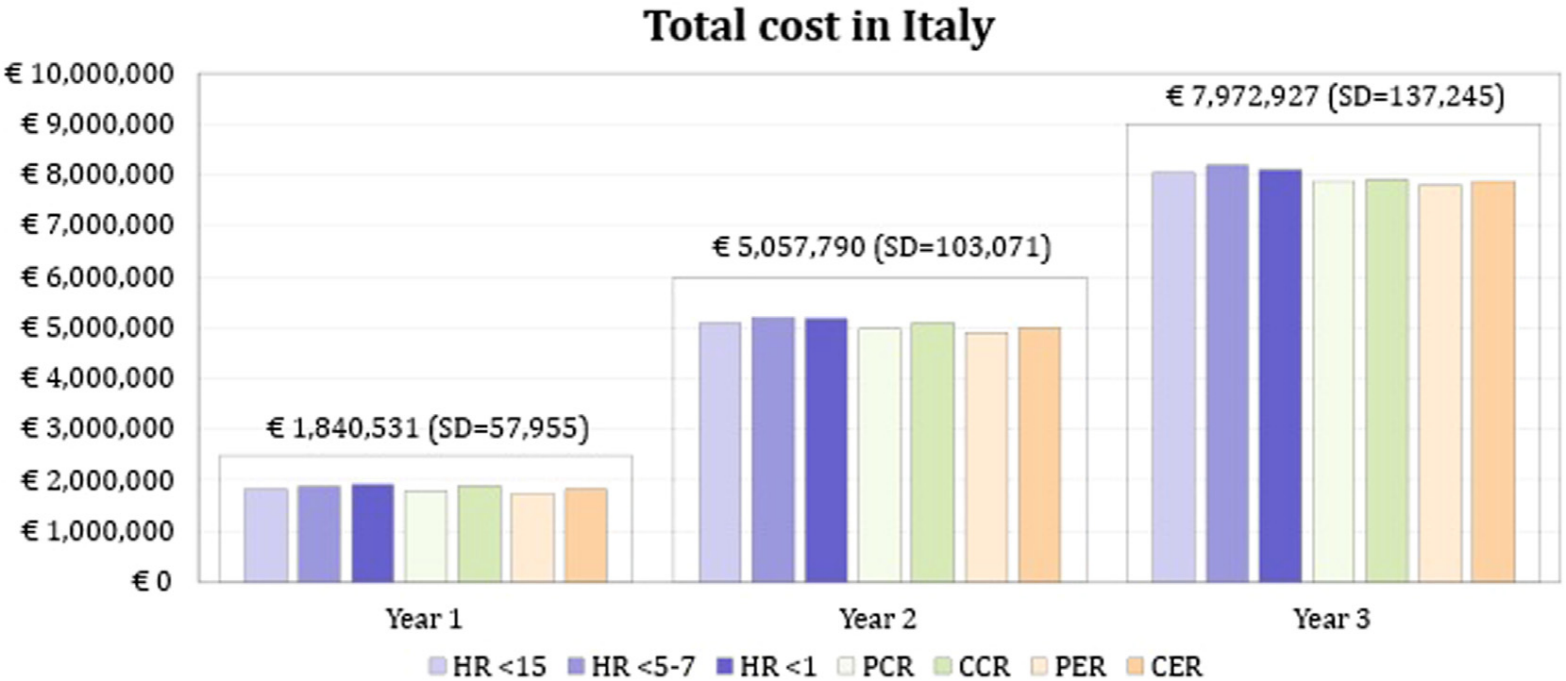
Total EoE cost in Italy for the next 3 years. *CCR*, Complete clinical response; *CER*, complete endoscopic response; *PCR*, partial clinical response; *PER*, partial endoscopic response.

Finally, the economic burden for the estimated patients with EoE treated in Italy in the next 3 years amounted to €14.9 million: €1.8 million in year 1, €5.1 million in year 2, and 8.0 million in year 3 (Fig 5).

## Discussion

This systematic review included 78 publications with a total of 9477 patients analyzed, providing a picture on the actual evidence about the efficacy, safety, and economic impact of TCS and PPI treatment in EoE management. Our study showed that the most used first-line therapy was PPIs (48.4%), followed by TCSs (33.1%) and FEDs (10.5%); less than 10% of patients were treated with a combination of PPIs, TCSs, or FEDs in first line. This distribution clearly reflected guidelines recommendations in which no clear preference between TCSs and PPIs as first-line therapy was stated.^34^, ^35^, ^36^ A different trend was instead observed in second-line treatments in which TCSs were at the top in usage distribution, followed by PPI + TCS, PPIs, PPI + TCS + FED, FEDs, and TCS + FED.

Efficacy was assessed using different measures among all studies, aside from ER. As for HR, the most prevalent threshold was less than or equal to 15 eos/hpf (65 studies), followed by less than or equal to 5-7 eos/hpf (6 studies) and lastly less than or equal to 1 eos/hpf (2 studies). The highest heterogeneity was found in CR wherein the most common validated score was the Dysphagia Severity Score (23.5%), followed by the Mayo Dysphagia Questionnaire (17.6%), the Dysphagia Symptom Questionnaire and Eosinophilic Esophagitis Activity Index (11.8%), the Watson Dysphagia Scale (8.8%), and the Physician Global Assessment (2.9%). In addition, 23.5% of the included studies evaluated CR with a nonvalidated but objective score. Ultimately, ER was the only outcome for which just 1 score (ie, EREFS) was used. Although for HR the threshold of less than or equal to 15 eos/hpf could prevail as the standard threshold in the near future, these findings might suggest the need of 1 consistent and validated score when assessing CR.

In terms of efficacy results, this analysis pointed out that TCSs were superior to PPIs in HR (all thresholds), ER (both partial and complete), and partial CR, whereas the converse was true just for complete CR. When differentiating the analysis between study type (RCTs vs observational), no significant differences were found except for HR and partial CR in TCSs. A significant point to highlight is the relapse rate after treatment discontinuation; in fact, our study showed that the relapse rate in patients interrupting versus continuing TCS therapy was 0.70 cases (median time, 7 months) versus 0.26 cases (median time, ≥1 year) per PY, suggesting that there could be a potential risk of suffering from an easier relapse as a result of early discontinuation. Yet, this result should be taken cautiously because according to the exploratory analysis on the association between treatment duration and TCS safety (see Table E1 in this article’s Additional Material 5 section in the Online Repository), most of the adverse events were more frequent in mid- to long-term follow-up studies (ie, patients treated for >12 weeks); proportion of patients experiencing any TEAEs was 57.6% in short-term follow-up studies, 61.0% in mid-term follow-up studies, and 73.1% in long-term follow-up studies (*P* < .001). The same trend was observed in the proportion of patients experiencing dilation, any infection, upper respiratory tract infection, and skin disorders. However, most of the TEAEs observed in the included studies were nonserious and the discontinuation rate, as well as hospitalization due to adverse events, was very low.

As expected, the rate of adverse events was higher in RCTs than in observational studies. This was probably because in the RCTs, reporting TEAEs was mandatory, whereas in the observational studies, particularly in retrospective studies, the information could not be available. Finally, dilations on treatment were moderately needed for both on and off TCS treatment (0.3 cases per PY), indicating that conventional therapies still have major issues in improving patient QoL.

Subgroup analysis highlighted that efficacy was higher, on average, in the studies in which EoE diagnosis was made according to the AGREE Guidelines,^10^ that is, those studies that did not exclude PPI-REE from the enrollment. Specifically, HR with PPIs and SMD in symptoms score with TCSs were significantly higher in this subgroup. This effect could be due to 2 reasons: (1) patients with PPI-REE were likely to respond to further therapy and (2) studies that have enrolled patients with PPI-REE were investigations that have been conducted mainly after 2018, and among them those that have evaluated the ODT formulation (more effective) are more common. For the same reason, efficacy was also higher in studies conducted in Europe with respect to those conducted in the United States where the ODT formulation was not available.

Evidence comparing the efficacy of different treatments in EoE is very heterogeneous in terms of results and nowadays there is still no actual recommendation on whether TCSs or PPIs should be used as standard care in everyday clinical practice.^34^, ^35^, ^36^ However, similar results wherein TCS treatment was associated with better clinical outcomes with respect to PPIs were found in a recent network MA by Visaggi et al.^37^

Moreover, the present study provided an estimate of the economic burden of EoE in Italy. About 3000 patients were expected to start a treatment based on PPIs or TCSs every year, and the total economic impact on the National Health Service for the next 3 years was estimated to be €14.9 million. Dilation and relapse management were the main cost drivers accounting for 44% and 29% of the total cost, respectively. Besides, the limited efficacy of PPIs (used off-label generally) and TCSs associated with high risk of relapse, infections, esophageal candidiasis, and upper respiratory tract infections could have a significant impact on patient QoL and on the onset of “adaptive behaviors.” Specifically, EoE has a significant negative impact on mental rather than physical health^38^^,^^39^; therefore, more effective therapeutic strategies and provision of sufficient mental care could allow the National Health Service spending to be managed differently with a positive impact on patient QoL.

In the present study, we observed that blood cortisol decrease was the only mild to moderate event that occurred, but treatment discontinuation was able to solve the issue without clinical complications. However, this implies the potential need of cortisol assessment to prevent any complications. Studies reported low serum cortisol values in a remarkable number of subjects treated with TCSs, testifying that systemic exposure to corticosteroids may occur even with these topical formulations.^40^ However, the clinical impact of such exposure was not clarified in either short-term or long-term studies. Specifically, it was unknown whether the low cortisol values might be associated with systemic side effects, such as infections, skeletal fragility, or neuroendocrine disorders (eg, tertiary hypoadrenalism, hypogonadism, and growth hormone deficiency) that may occur in subjects treated with TCSs.^16^^,^^41^^,^^42^ Future studies will clarify whether serum cortisol values might be used to guide monitoring of TCS safety in EoE, such as already proposed in other clinical settings.^43^

This study has some limitations that should be addressed. The first is the use of different thresholds to define histologic remission in included studies. However, the varying sizes of eos/hpf were standardized as much as possible in 3 cutoffs, which represent the main histologic outcomes of interest.^6^ Specifically, thresholds less than or equal to 5, 6, or 7 eos/hpf were considered as the same category. Similarly, although complete response was measured according to different scales and scores, it was chosen to use only an objective score, that is, by excluding articles that assessed the CR on the basis of clinical judgment only. Another limitation is that the partial ER was defined on the basis of more than 50% improvement in EREFS or EREFS less than or equal to 2 at follow-up. Recent publications suggested to use only the second definition^44^^,^^45^; however, the number of studies that reported partial ER was limited, and by combining the 2 definitions we increased the sample size for the analysis of this outcome. Moreover, short- to mid-term follow-up articles were more frequent among the included studies; hence, the TCS safety evaluation could lack long-term follow-up evidence. Again, although we performed subgroup analyses and MR, the heterogeneity remained high, suggesting that more homogeneous data are required. The last limitation is the representativeness of adolescents in the included studies. In our review, the mean proportion of adolescents was 5%, which is slightly lower than the proportion observed in real life both in the Sweden cohort from the study by Plate et al^46^ (9%) and in the UK cohort from the study by Xu et al^47^ (7.8%). However, it should be considered that RCTs are more likely to include only patients 18 years or older even if the target population could be younger. Moreover, on the basis of articles included in this review, age does not seem to be associated with drug response. Specifically, in the study by Dellon et al^48^ (18% adolescents), there is not a significant difference in the mean age (mean difference, 0.9 years; 95% CI, −3.7 to 5.4) or in the proportion of adolescents (odds ratio, 0.69; 95% CI, 0.21 to 2.25) between responders and nonresponders to budesonide. Furthermore, the MR performed on the variable “mean age” confirmed that there is no increase (or decrease) in the response (histologic, clinical, or endoscopic) or in the incidence of dilation or candidiasis because the effect of age varied between 0.95 and 1.03, proving an extremely low effect (see Table E3 in Additional Material 5 section in the Online Repository).

Some points of strength should also be highlighted. To our knowledge, the present analysis is the most updated (from any date up to December 31, 2023) and comprehensive literature review on the role of PPIs and TCSs in the management of patients with EoE aged 12 years or older. The inclusion of observational studies, rather than RCTs only, permitted to evaluate the efficacy and safety of treatments also in a real-life setting. Despite some difference in the magnitude of the estimated effects, RCTs and observational studies agreed for almost all outcomes. Finally, on the basis of the bias assessment, the overall quality of the included studies (both RCTs and observational studies) was high.

This systematic review provides valuable insights into the efficacy, safety, and economic impact of TCS and PPI treatment in EoE. TCS treatment emerged as the primary choice for achieving HR and ER, whereas PPI treatment showed efficacy in reducing symptoms. The study highlights the need for standardized treatment guidelines because of the heterogeneity in treatment efficacy across different studies and the existence of a significant unmet need that could be fulfilled by new biologic therapies as anti–IL-4 and IL-13.^49^ However, on the basis of the available studies, it must be acknowledged that heterogeneity in both eligibility criteria and outcome measures hampers the establishment of a solid therapeutic hierarchy in EoE. Because of the low representativeness of adolescents, generalization on patients aged 12 to 17 years could raise some concerns. Future research and the introduction of new therapies may offer opportunities to optimize treatment outcomes and health care costs in EoE management, ultimately improving patient QoL.

## Disclosure statement

10.13039/100022824Sanofi Italy is the sponsor and funder of this research. AdRes assumes the responsibility for designing the project’s protocol and analysis plan, performing the systematic review and MA, as well as report generation. The sponsor will be involved in the review and approval of relevant project documents.

Disclosure of potential conflict of interest: G. W. Canonica reports research or clinical trial grants paid to his institution from Menarini, 10.13039/100004325AstraZeneca, 10.13039/100004330GlaxoSmithKline, and Sanofi-Genzyme; and reports fees for lectures or advisory board participation from Menarini, AstraZeneca, Celltrion, Chiesi, Faes Farma, Firma, Genentech, Guidotti-Malesci, GlaxoSmithKline, HAL Allergy, Innovacaremd, Novartis, OM Pharma, Red Maple, Sanofi-Aventis, Sanofi-Genzyme, Stallergenes, and Uriach Pharma. G. Mazziotti received fees for consultancy and preceptorship from Amgen-UCB; and received fees for lectures from Theramex and Recordati. A. Repici received consultancy fees from Medtronic, Fuji, Olympus, and Erbe; and received research grants and speaker’s fees from Boston Scientific, Erbe, Alfasigma, and Norgine. E. V. Savarino has served as speaker for AbbVie, Abivax, Agave, AGPharma, Alfasigma, CaDiGroup, Celltrion, Dr Falk, EG Stada Group, Fenix Pharma, Galapagos, Johnson & Johnson, JB Pharmaceuticals, Innovamedica/Adacyte, Eli Lilly, Malesci, Mayoly Biohealth, Omega Pharma, Pfizer, Reckitt Benckiser, Sandoz, SILA, Sofar, Takeda, Tillots, and Unifarco; has served as consultant for AbbVie, Agave, Alfasigma, Biogen, Bristol-Myers Squibb, Celltrion, Dr Falk, Eli Lilly, Fenix Pharma, Johnson & Johnson, JB Pharmaceuticals, Merck & Co, Nestlé, Pfizer, Reckitt Benckiser, Regeneron, Sanofi, SILA, Sofar, Takeda, and Unifarco; and received research support from Bonollo, Difass, Pfizer, Reckitt Benckiser, SILA, Sofar, Unifarco, and Zeta Farmaceutici. L. Pradelli is co-owner and employee of AdRes, which has received project funding from Sanofi. L. Castello and M. Povero are employees of AdRes, which has received project funding from Sanofi. M. Dobreva, F. Fanelli, and J. P. Saab are employees of Sanofi and may hold shares and/or stock options in the company.
