# Supplementary material for: The clinical impact of conventional therapies for adults and adolescents suffering from eosinophilic esophagitis, a type 2 inflammatory chronic disease, and their economic consequences in Italy: Systematic literature review and meta-analysis
Source: J Allergy Clin Immunol Glob. 2024 Dec 11;4(1):100383. doi: 10.1016/j.jacig.2024.100383 (PMC11773236; doi:10.1016/j.jacig.2024.100383)
Supplement: Supplementary Figs and Tables [file mmc1.docx]

# Additional Material 1

Table E1. Search string used in MEDLINE

| MEDLINE |
| --- |
| Search:  ("eosinophilic esophagitis"[MeSH Terms] OR "eosinophilic esophagitis"[Title/Abstract])  AND  ("Adrenal Cortex Hormones"[MeSH Terms] OR "Corticosteroids"[Title/Abstract] OR "Corticosteroid"[Title/Abstract] OR "Corticoids"[Title/Abstract] OR "Corticoid"[Title/Abstract] OR "adrenal cortex hormone"[Title/Abstract] OR "Adrenal Cortex Hormones"[Title/Abstract] OR "Proton Pump Inhibitors"[MeSH Terms] OR "proton pump inhibitor"[Title/Abstract] OR "Proton Pump Inhibitors"[Title/Abstract])  AND  ("adolescent"[MeSH Terms] OR "adult"[MeSH Terms])  Filters: **Adolescent: 13-18 years, Adult: 19+ years** |

Table E2. Search string used in EMBASE

| EMBASE |
| --- |
| 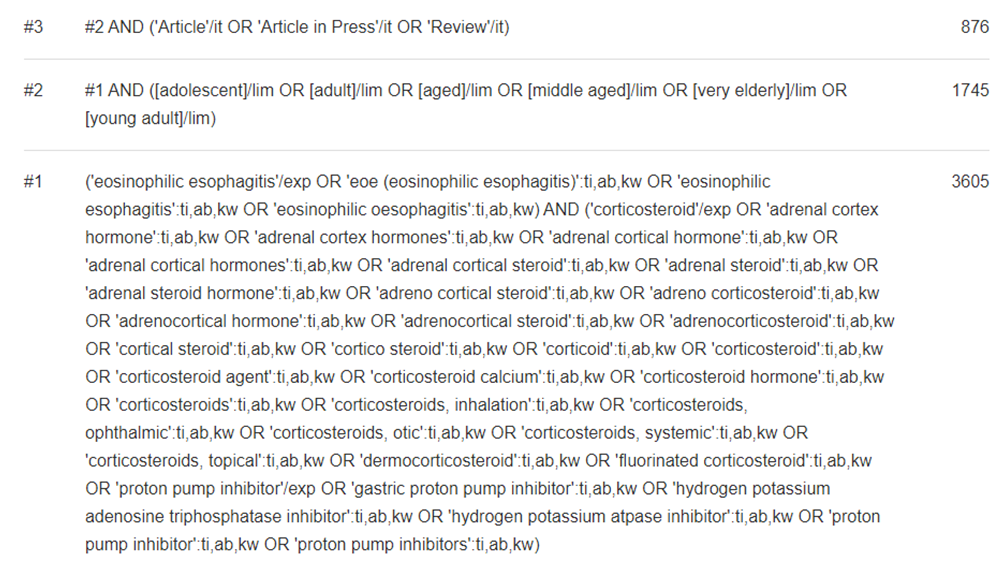 |

Table E3. Search string used in Cochrane Library

| Cochrane Library |
| --- |
| 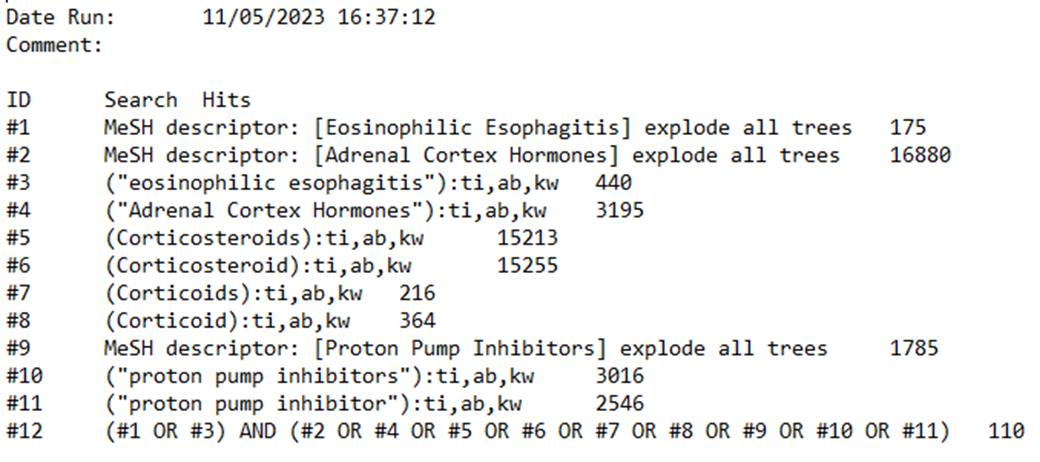 |

Table E4. Scores used to evaluate clinical response.

| Score/questionnaire | Description |
| --- | --- |
| Dysphagia Severity Score (DSS) | Measuring instrument developed by Alex Straumann and colleagues in 2010. DSS assesses frequency of dysphagia, ranging from none (0) to several times per day (5); the intensity of dysphagia, ranging from unhindered swallowing (1) to long-lasting complete obstruction requiring endoscopic intervention (5); and the duration of dysphagia, ranging from no attacks (0) to lasting up to endoscopic removal of the impacted food (5). Total scores range from 1 to 15. |
| Eosinophilic Esophagitis Activity Index (EEsAI) | 7-day recall period, ranges from 0 to 100 with a higher number indicating greater symptom severity |
| Mayo Dysphagia Questionnaire (MDQ) | The MDQ-2 weeks (17-item instrument) or MDQ-30 days (28-item instrument) assessing dysphagia, heartburn, and regurgitation symptoms. In each symptom domain, items address duration, frequency, and severity. |
| Dysphagia Symptom Questionnaire (DSQ) | The DSQ is a 3-question daily diary that has been validated for the measurement of dysphagia frequency and severity in patients with EoE. The questions ask whether solid food has been eaten; whether food has gone down slowly or become stuck; and what, if any, measures have been taken to achieve relief. DSQ scores are calculated on the basis of the responses to these questions over a 2-week period. The scores for questions 2 and 3 are summed, then divided by the number of days for which the diary has been completed and multiplied by 14. Scores can range from 0 to 84, with higher values indicating more frequent and severe dysphagia. |
| Watson Dysphagia Scale (WDS) | The WDS gives a score ranging from 0 (no dysphagia) to 45 (severe dysphagia) on a 9-item scale (from liquids to solid food). The presence of any dysphagia for each liquid or solid substance is determined and scored by the patient on a 3-point Likert scale (1 = always, 0.5 = sometimes, and 0 = never). This score is then multiplied by a factor for each substance, and the scores for all substances are summed. |
| Physician Global Assessment (PGA) | Visual analog scale 0–100 mm from no disease to worst possible disease. |

# Additional Material 2: Newcastle Ottawa quality assessment scale

**Selection**

1) Representativeness of the exposed cohort

1. truly representative of the average _______________ (describe) in the community **(1 point)**
2. somewhat representative of the average ______________ in the community **(1 point)**
3. selected group of users eg nurses, volunteers
4. no description of the derivation of the cohort

2) Selection of the non-exposed cohort

1. drawn from the same community as the exposed cohort **(1 point)**
2. drawn from a different source
3. no description of the derivation of the non-exposed cohort

3) Ascertainment of exposure

1. secure record (eg surgical records) **(1 point)**
2. structured interview **(1 point)**
3. written self-report
4. no description

4) Demonstration that outcome of interest was not present at start of study

1. yes **(1 point)**
2. no

**Comparability**

1) Comparability of cohorts on the basis of the design or analysis

1. study controls for _____________ (select the most important factor) **(1 point)**
2. study controls for any additional factor **(1 point)**

**Outcome**

1) Assessment of outcome

1. independent blind assessment **(1 point)**
2. record linkage **(1 point)**
3. self-report
4. no description

2) Was follow-up long enough for outcomes to occur

1. yes (select an adequate follow up period for outcome of interest) **(1 point)**
2. no

3) Adequacy of follow up of cohorts

1. complete follow up - all subjects accounted for **(1 point)**
2. subjects lost to follow up unlikely to introduce bias - small number lost - > ____ % (select an adequate %) follow up, or description provided of those lost) **(1 point)**
3. follow up rate < ____% (select an adequate %) and no description of those lost
4. no statement

# Additional Material 3

Table E1. Description of key characteristics in studies included in the MA.

| Characteristic | | Description |
| --- | --- | --- |
| 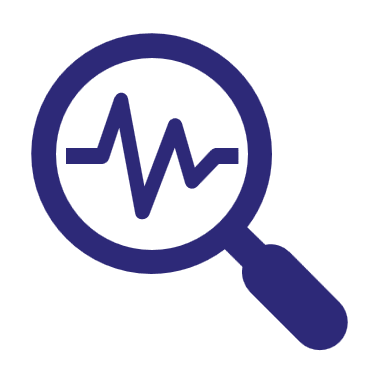 | Study type | 44.9% retrospective observational, 29.5% prospective observational, 19.2% RCT, 5.1% cross-sectional, 1.3% retrospective + prospective observational |
| 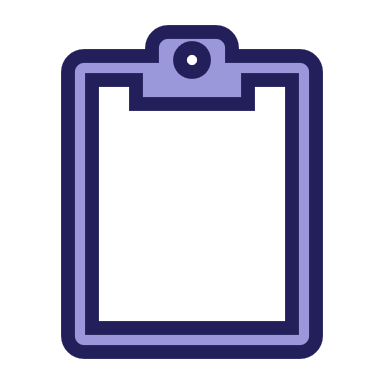 | Guidelines | 62.3% pre 2018 GL, 36.4% post 2018 GL and 1.3% both pre and post 2018 GL |
| 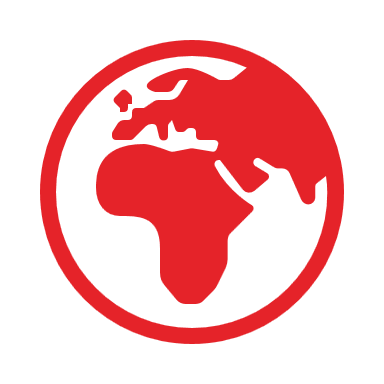 | Country | 50.6% US, 35.1% Europe, 10.4% Asia, 2.6% US and Europe and 1.3% Australia |
| 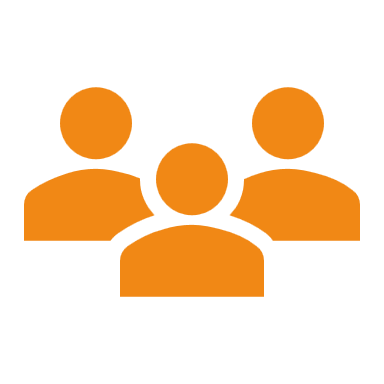 | Enrolled patients | Mean 122 (range 11-1044) |
| 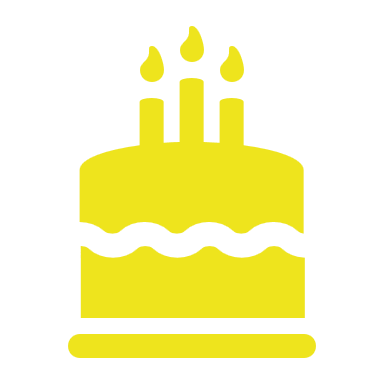 | Age at enrollment | Mean 37.8 years (range 14-51), 5% adolescents (age 12-18) |
| 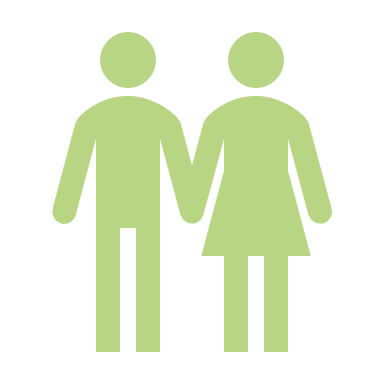 | Gender (% male) | Mean 71.0% (range 27-95%) |
| 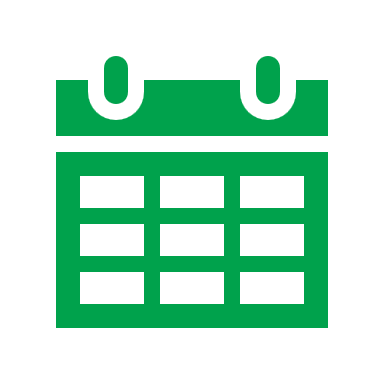 | Symptoms duration | Mean 7.5 years (range 1-13) |

Figure E1. Main symptoms observed at enrollment in studies included in the MA


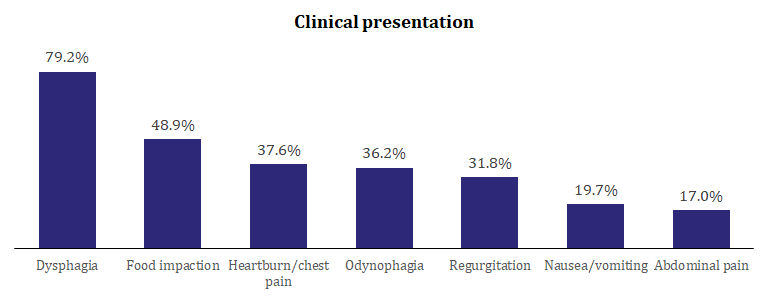


Figure E2. Main endoscopic findings observed at enrollment in studies included in the MA


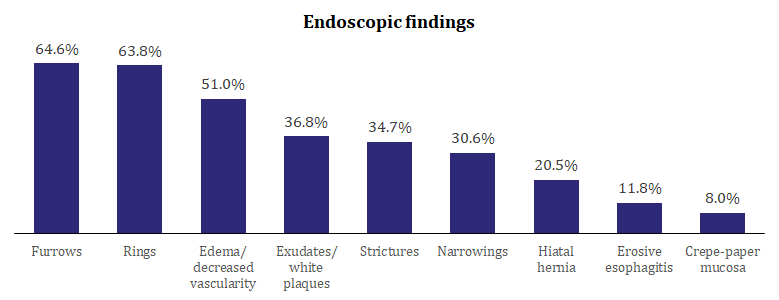


Figure E3. Main atopic comorbidities observed at enrollment in studies included in the MA


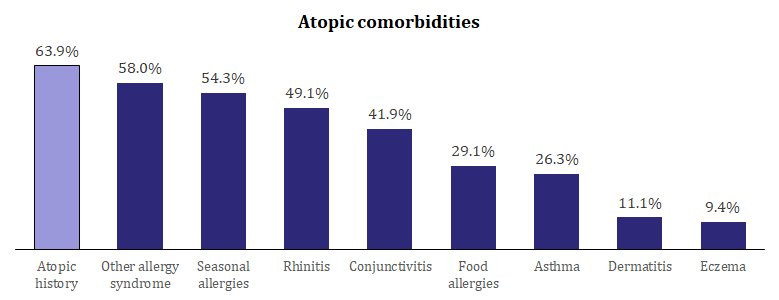


Figure E4. Bias assessment evaluation of RCTs included in the SLR according to Newcastle Ottawa scale


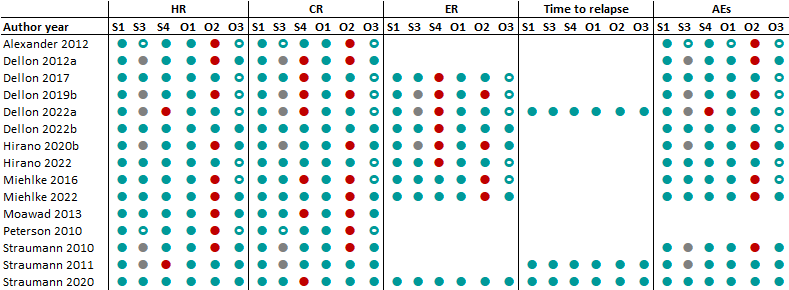


S1 Representativeness of the exposed cohort:  truly representative;  somewhat representative;  selected group;  no description

S3 Ascertainment of exposure:  secure record;  structured interview;  self-report;  no description

S4 Demonstration that outcome of interest was not present at start of study:  yes;  no

O1 Assessment of outcome:  independent blind;  record linkage;  self-report;  no description

O2 Follow-up long enough for outcomes to occur:  yes;  no

O3 Adequacy of follow up of cohorts:  complete follow-up;  unlikely bias;  likely bias;  no description

Figure E5. Bias assessment evaluation of observational studies included in the SLR according to Newcastle Ottawa scale


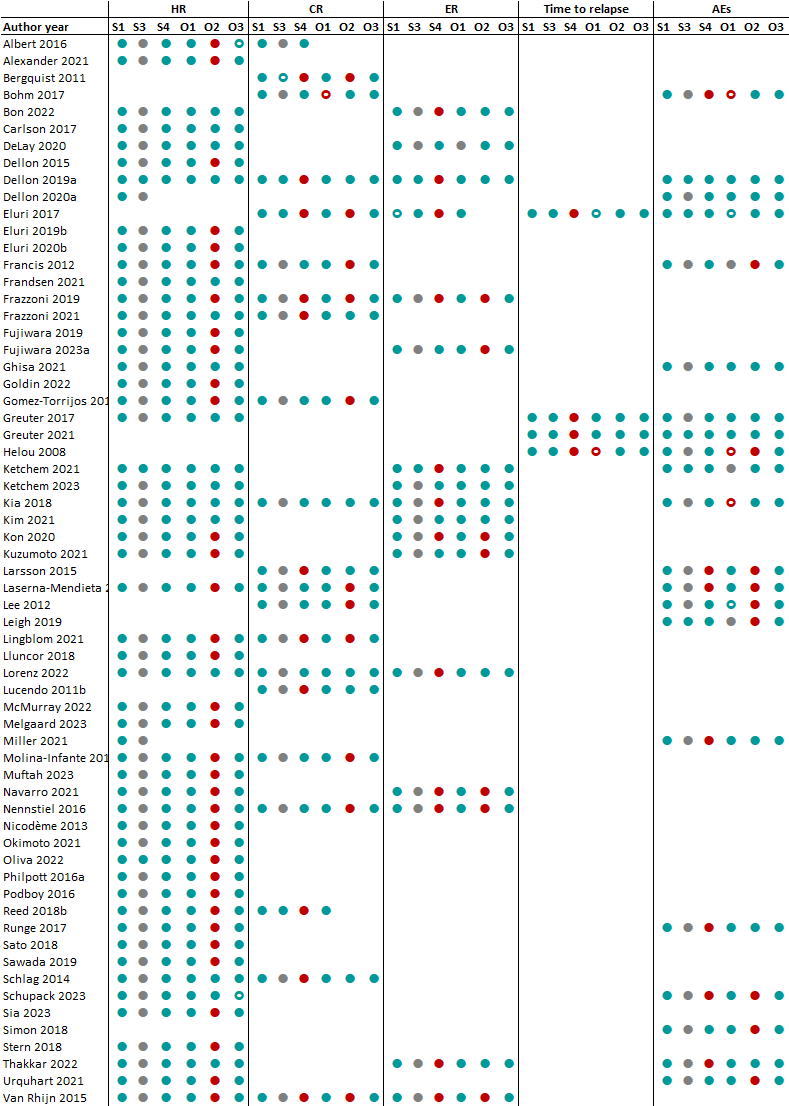


S1 Representativeness of the exposed cohort:  truly representative;  somewhat representative;  selected group;  no description

S3 Ascertainment of exposure:  secure record;  structured interview;  self-report;  no description

S4 Demonstration that outcome of interest was not present at start of study:  yes;  no

O1 Assessment of outcome:  independent blind;  record linkage;  self-report;  no description

O2 Follow-up long enough for outcomes to occur:  yes;  no

O3 Adequacy of follow up of cohorts:  complete follow-up;  unlikely bias;  likely bias;  no description

Figure E6. Proportion of studies included divided by study type and outcome


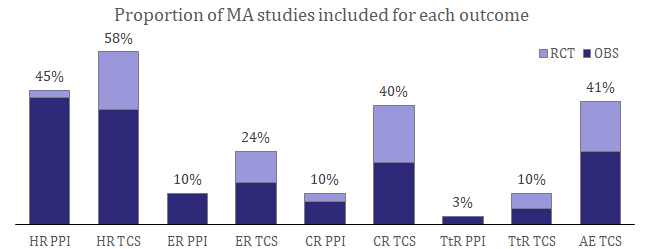


# Additional Material 4

Table E1. Main characteristics of included Randomized Controlled Trials in patients with active EoE

| Study and year | Country and period | Population (Mean age; % male) and definition of active EoE | Treatments included (No. of patients) and duration of therapy | Histological endpoint (proportion of patients achieving eos/hpf threshold) | Endoscopic endpoint | Symptomatic endpoint | Response duration | Reported adverse events or number of dilations |
| --- | --- | --- | --- | --- | --- | --- | --- | --- |
| **Alexander 2012** | US 2005-2009 | 37.0 years  85.7%  Pre 2018 GL with confirmed cases of EoE having ≥20 eos/hpf | aFLU (n=21) 880mcg BID for 6 weeks | >50% (partial response) or >90% (complete response) reduction in mean eos/hpf count from baseline^1^ | Not assessed | Proportion of patients  achieving complete or partial  symptomatic response based on MDQ^2^ | Not assessed | Side effects assessed with questionnaire, number of dilations |
| **Dellon 2012a** | US 2010-2011 | 35.0 years  60.0%  Pre 2018 GL | nBUD or vBUD (n=22) 1mg BID for 8 weeks | <1/7/15 eos/hpf | Not assessed | Symptoms response  assessed with MDQ (score 0-100) with no specified threshold | Not assessed | Any serious and non-serious TEAE, number of dilations |
| **Dellon 2017** | US 2012-2014 | 22.3 years  69.0%  Pre 2018 GL | vBUD (n=51) 2mg BID for 12 weeks | <6 eos/hpf | Change in EREFS score at FU^3^ | Proportion of patients with  ≥30% reduction in DSQ  score compared with  baseline | Not assessed | Any serious and non-serious TEAE |
| **Dellon 2019b** | US 2014-2018 | 37.6 years  64.1%  Pre 2018 GL | aFLU 880mcg or vBUD 1mg BID (n=129) for 8 weeks | <1/5/15 eos/hpf | Change in EREFS score at FU^3^ | Improvement in DSQ (score 0-84) with no specified threshold | Not assessed | Any serious and non-serious TEAE |
| **Dellon 2022a** | US 2016-2019 | 34.1 years  61.1%  Pre 2018 GL | BUD ODT (n=154) 2mg BID for 36 weeks | <1/6/15 eos/hpf | Change in EREFS score at FU | Proportion of patients with  ≥30% reduction in DSQ  score compared with  baseline | Number of relapses at 36 weeks | Any serious and non-serious TEAE |
| **Dellon 2022b** | US and Europe 2017-2018 | 39.3 years  68.0%  Pre 2018 GL | FLU ODT 1.5mg QD/BID or FLU ODT 3mg QD/BID (n=84) for 52 weeks | <1/6/15 eos/hpf | Change in EREFS score at FU | Change in  EEsAI at FU | Not assessed | Any serious and non-serious TEAE |
| **Hirano 2020b** | US 2011-2012 | 24.0 years  62.5%  Pre 2018 GL with confirmed cases of EoE having ≥24 eos/hpf | FLU ODT (n=8) 1.5mg BID or FLU ODT (n=8) 3mg QD for 8 weeks | 0/<15 eos/hpf | Change in EREFS score at FU | Change in EEsAI and PGA at FU | Not assessed | Any serious and non-serious TEAE |
| **Hirano 2022** | US 2015-2019 | 33.8 years  60.6%  Post 2018 GL | BUD (n=213) 2mg BID for 12 weeks | <1/6/15 eos/hpf | Change in EREFS score at FU | Proportion of patients with  ≥30% reduction in DSQ  score compared with  baseline | Not assessed | Any serious and non-serious TEAE |
| **Miehlke 2016** | Europe  Period not reported | 40.8 years  82.47%  Pre 2018 GL | BUD (n=19) 2mg QD for 2 weeks  BUD (n=19) 2mg BID for 2 weeks  vBUD (n=19) 2mg QD for 2 weeks | <5 eos/hpf | Improvement in endoscopic abnormalities at FU assessed by an endoscopic intensity score | Change in dysphagia intensity symptoms at FU | Not assessed | Any serious and non-serious TEAE |
| **Miehlke 2022** | Europe 2016-2018 | 36.0 years  80.7%  Post 2018 GL | BUD ODT (n=181) 1mg BID for 6 weeks | 0/<5 eos/hpf | Change in EREFS and proportions of patients in deep endoscopic remission at FU^3^ | Change in EEsAI and proportion of patients with EEsAI ≤20 at FU | Not assessed | Any serious and non-serious TEAE |
| **Moawad 2013** | US 2008-2010 | 38 years  90.5%  Post 2018 GL | High dose PPI (n=21) or FLU 440mcg (n=21) BID for 8 weeks | <7 eos/hpf | Not assessed | Change in dysphagia symptoms at FU assessed with MDQ | Not assessed | Not assessed |
| **Peterson 2010** | US 2005-2006 | 37 years  76.7%  Post 2018 GL | High dose PPI (n=15) and aFLU (n=15) 440mcg BID for 8 weeks | <5/<15 eos/hpf | Not assessed | Reduction of at least 2 points in the dysphagia scale score (0-7) at FU | Not assessed | Not assessed |
| **Straumann 2010** | Europe 2006-2007 | 33.1 years  94.4%  Post 2018 GL with confirmed cases of EoE having ≥20 eos/hpf | BUD (n=18) 1mg BID for 2 weeks | <5/<20 eos/hpf | Not assessed | Reduction in dysphagia score (0-9) of at least 3 points at FU | Not assessed | Infection and infestation TEAE, number of dilations |
| **Straumann 2011** | Europe 2006-2007 | 38.0 years  92.9%  Post 2018 GL with confirmed cases of EoE having ≥20 eos/hpf | BUD (n=14) 0.5mg BID for 50 weeks | <5/<20 eos/hpf | Not assessed | Reduction in dysphagia score (0-9) of at least 3 points at FU | Number of relapses at 50 weeks | Infection and infestation TEAE |
| **Straumann 2020** | Europe 2016-2018 | 36.5 years  83.8%  Post 2018 GL | BUD ODT 0.5 mg BID (n=68) and BUD ODT 1mg BID (n=68) for 48 weeks | <5 eos/hpf | Deep endoscopic remission assessed with EREFS at FU (total score=0) | Change in EEsAI score at FU | Number of relapses at 48 weeks | Number of dilations |

^1^ Based on the pretreatment mean eos/hpf count, a reduction in more than 90% and 50% from the baseline value is the same as setting a <5 and <15 eos/hpf threshold.

^2^ A complete symptom response was defined as an answer of “no” to the question, “In the past 2 weeks, have you had trouble swallowing, not associated with other cold symptoms (such as strep throat or mononucleosis)?”. A partial symptom response was defined as an answer of “yes” to the earlier-described question and a decrease in severity of at least 2 levels (or to a level of “Doesn’t bother me at all”), or a decrease in frequency of at least 1 level.

^3^ Also available for each individual EREFS subscore.

**Abbreviations:** **aFLU**, aerosolized fluticasone; **aMF**, aerosolized mometasone furoate; **aMF**, aerosolized mometasone furoate; **BID**, bis in die; **BUD ODT**, budesonide orally disintegrated tablet; **BUD**, budesonide; **cFLU**, compounded fluticasone; **CR**, clinical response; **DSS**, Dysphagia Symptoms Score; **EoE**, eosinophilic oesophagitis; **Eos/hpf**, eosinophils/high-power field; **EREFS**, EoE Endoscopic Reference Score; **ESO**, esomeprazole; **FED**, Food Elimination Diet; **FLU ODT**, Fluticasone orally disintegrated tablet; **FLU**, fluticasone; **FU**, follow-up; **GL**, guidelines; **MDQ**, Mayo Dysphagia Questionnaire; **nBUD**, nebulized budesonide; **OME**, omeprazole; **PGA**, Patient Global Assessment; **PPI**, Proton-pump inhibitor; **QD**, quaque die; **RABE**, rabeprazole; **SDI**, Straumann Dysphagia Index; **TCS**, topical corticosteroids; **TEAE**, treatment emergent adverse event; **US**, United States; **VAS**, Visual Analogue Scale; **vBUD**, viscous budesonide; **WDS,** Watson Dysphagia Scale.

Table E2. Main characteristics of included observational studies in patients with active EoE

| Study and year | Country and period | Population (Mean age; % male) and definition of active EoE | Treatments included (No. of patients) and duration of therapy | Histological endpoint (proportion of patients achieving eos/hpf threshold) | Endoscopic  endpoint | Symptomatic endpoint | Response duration | Reported adverse events or number of dilations |
| --- | --- | --- | --- | --- | --- | --- | --- | --- |
| **Albert 2016** | US 2008-2013 | 38.6 years  88.0%  Pre 2018 GL | aFLU 440mcg or vBUD/BUD 1mg (n=56) BID for 8 weeks | <15 eos/hpf | Not assessed | Not assessed | Not assessed | Not assessed |
| **Alexander 2021** | US 2011-2018 | 40.6 years  60.5%  Pre 2018 GL | OME 20mg (n=223) BID for 8 weeks | <15 eos/hpf | Not assessed | Not assessed | Not assessed | Not assessed |
| **Bergquist 2011** | Europe 2009 | 45.0 years  74.2%  Pre 2018 GL | MF 200mcg (n=31) QID for 8 weeks | Not assessed | Not assessed | Change in dysphagia score at FU assessed with WDS | Not assessed | Not assessed |
| **Bohm 2017** | US  Period not reported | 21.0 years  64.0%  Pre 2018 GL | PPI (n=14  and TCS therapy (n=7)  Posology and treatment duration not reported | Not assessed | Not assessed | Symptoms resolution (score ≤15) assessed with MDQ | Not assessed | Number of dilations |
| **Bon 2022** | Europe  Period not reported | 38.9 years  77.4%  Pre 2018 GL | TCS 1mg (n=159) BID until CR then 0.25mg BID during maintenance up to 260 weeks | <15 eos/hpf | Change in EREFS at FU | Not assessed | Not assessed | Not assessed |
| **Carlson 2017** | US 2013-2016 | 30.0 years  77.8%  Pre 2018 GL | PPI (n=12) and TCS therapy (n=8) for 14.6 weeks  Posology not reported | <15 eos/hpf | Not assessed | Not assessed | Not assessed | Not assessed |
| **DeLay 2020** | US 2013-2017 | 27.2 years  66.1%  Pre 2018 GL | PPI therapy (n=769) for 124 weeks  No posology reported | <15 eos/hpf | Documented endoscopic benefit with no specific definition or threshold | Not assessed | Not assessed | Not assessed |
| **Dellon 2015** | US 2011-2013 | 38.8 years  58.0%  Pre 2018 GL | aFLU 880mcg or BUD 1mg (n=61) BID for 8 weeks | <1/15 eos/hpf | Not assessed | Not assessed | Not assessed | Not assessed |
| **Dellon 2019a** | US 2012-2014 | 21.8 years  70.7%  Pre 2018 GL | 12 weeks PBO + 12 weeks vBOS 2 mg BID (n=37) and 12 weeks vBOS 2 mg QID + 12 weeks vBOS 2 mg BID (n=45) | ≤1/6/15 eos/hpf | Change in EREFS at FU | Change in PGA score at FU | Not assessed | Any serious and non-serious TEAE |
| **Dellon 2020a** | US 2014-2018 | 41.7 years  64.0%  Pre 2018 GL | aFLU 880mcg or vBUD 1mg BID (n=58) for 1 year | Not assessed | Not assessed | Not assessed | Number of relapses within 1 year | Number of dilations |
| **Eluri 2017** | US 2006-2015 | 39.9 years  67.0%  Pre 2018 GL | BUD 0.5-1mg or FLU 440-880mcg BID (n=55) for 8 weeks | Not assessed | Change in EREFS at FU | Not assessed | Number of relapses at 29.6 months | Any infection or infestation TEAE |
| **Eluri 2019b** | US 2009-2015 | 36.3 years  60.0%  Pre 2018 GL | aFLU 880mcg or vBUD 1mg BID (n=97) for 8 weeks | <1/15 eos/hpf | Not assessed | Not assessed | Not assessed | Not assessed |
| **Eluri 2020b** | US 2002-2017 | 37.1 years  64.6%  Pre 2018 GL | BUD 0.5-1mg or FLU 440-880mcg (n=113) BID for 8 weeks | <15 eos/hpf | Not assessed | Not assessed | Not assessed | Not assessed |
| **Francis 2012** | US 2009-2010 | 39.0 years  61.0%  Pre 2018 GL | ESO 40mg BID (n=18) or BUD 1mg BID (n=28) for 6 weeks | <5 eos/hpf | Not assessed | Symptoms improvement defined as a reduction in the score of >2 levels at FU assessed by MDQ | Not assessed | Skin disorders TEAE |
| **Frandsen 2021** | Europe 2007-2017 | 47.0 years  75.4%  Pre 2018 GL | High dose PPI therapy (n=118) for 11 weeks and low dose PPI therapy (n=98) for 17 weeks | <15 eos/hpf | Not assessed | Not assessed | Not assessed | Not assessed |
| **Frazzoni 2019** | Europe 2016-2018 | 39 years  76.7%  Post 2018 GL | PPI therapy (n=60) BID for 8 weeks | <15 eos/hpf | Change in EREFS at FU | Change in dysphagia symptoms score at FU assessed by a four grade Likert-type scale | Not assessed | Not assessed |
| **Frazzoni 2021** | Europe 2018-2020 | 38.0 years  85.0%  Post 2018 GL | OME 20mg (n=80) BID for 12 weeks | <15 eos/hpf | Not assessed | Change in dysphagia symptoms score at FU assessed by a four grade Likert-type scale | Not assessed | Not assessed |
| **Fujiwara 2019** | Asia 2010-2017 | 41.8 years  59.1%  Pre 2018 GL | PPI therapy (n=99) for 8 weeks | <15 eos/hpf | Not assessed | Not assessed | Not assessed | Not assessed |
| **Fujiwara 2023a** | Asia 2010-2022 | 45.0 years  66.9%  Post 2018 GL | ESO or RABE (n=159) for 8 weeks  Posology not reported | <1/15 eos/hpf | Complete response defined as EREFS=0 at FU and response defined as EREFS ≤2 at FU | Not assessed | Not assessed | Not assessed |
| **Ghisa 2021** | Europe 2012-2019 | 34.0 years  75.0%  Pre 2018 GL | PPI therapy (n=109) BID for 8 weeks or BUD/FLU 2mg QD for 120 weeks | <1/15 eos/hpf | Not assessed | Not assessed | Not assessed | Number of dilations |
| **Goldin 2022** | US 2006-2015 | 41.6 years  53.9%  Post 2018 GL | OME 40mg (n=104) BID for 8 weeks | <15 eos/hpf | Not assessed | Not assessed | Not assessed | Not assessed |
| **Gomez-Torrijos 2015** | Europe 2013-2015 | 41.0 years  62.5%  Pre 2018 GL | OME 40mg (n=120) BID for 8 weeks then gradually tapered to 20mg QD every other day | <5/15 eos/hpf | Not assessed | Clinical remission defined as symptoms reduction >50% at FU assessed by VAS-EoE score (0-10 points) | Not assessed | Not assessed |
| **Greuter 2017** | Europe 2007-2016 | 46.9 years  75.5%  Pre 2018 GL | BUD or FLU 1mg (n=351) BID then 0.25mg BID up to 61.4 weeks | <5 eos/hpf | Not assessed | Not assessed | Number of relapses (median FU 127 weeks)) | Infection and infestation TEAE |
| **Greuter 2021** | US and Europe  Period not reported | 37.2 years  70.7%  Pre 2018 GL | BUD or FLU median dose 0.5mg daily (n=82) for 114.4 weeks | Not assessed | Not assessed | Not assessed | Number of relapses (median FU 2.2 years) | Infection and infestation TEAE |
| **Helou 2008** | US 1999-2003 | 34.9 years  59.0%  Post 2018 GL | FLU 880mg (n=32) BID for 6 weeks | Not assessed | Not assessed | Not assessed | Number of relapses (median FU 3.3 years) | Infection and infestation TEAE |
| **Ketchem 2021** | US 2014-2019 | 33.1 years  66.7%  Pre 2018 GL | cFLU 2.5mg (n=27) QD for 22 weeks | <15 eos/hpf | Change in EREFS score at FU^1^ | Not assessed | Not assessed | Infection and infestation TEAE, number of dilations |
| **Ketchem 2023** | US  Period not reported | 36.6 years  63.2%  Pre 2018 GL | aFLU/cFLU 880/440mcg BID or vBUD 0.5/1 mg (n=296) BID up to 12 weeks | <1/6/15 eos/hpf | Change in EREFS score at FU and endoscopic response (improvement compared with baseline) assessed by endoscopist | Not assessed | Not assessed | Not assessed |
| **Kia 2018** | US  Period not reported | 36.3 years  77.5%  Pre 2018 GL | aFLU 50-1000 mcg (n=40) QID or BID for 17 weeks | <5/10/15 eos/hpf | Change in EREFS score at FU | Change in patient reported symptoms score and symptoms improvement (yes/no) assessed by a questionnaire | Not assessed | Infection and infestation TEAE |
| **Kim 2021** | Asia 2009-2019 | 50.7 years  78.6%  Post 2018 GL | PPI therapy (n=27) for 25 weeks and TCS therapy (n=7) for 14 weeks  Posology not reported | <15 eos/hpf | EREFS score <2 at FU | Not assessed | Not assessed | Not assessed |
| **Kon 2020** | Asia 2011-2018 | 49.0 years  85.2%  Post 2018 GL | PPI therapy (n=21) for 8 weeks | <15 eos/hpf | Change in EREFS at FU^1^ | Not assessed | Not assessed | Not assessed |
| **Kuzumoto 2021** | Asia 2010-2019 | 44.5 years  61.7%  Post 2018 GL | RABE 10mg (n=22) and 20mg (n=34) QID or ESO 20mg (n=25) QID for 8 weeks | <1/15 eos/hpf | EREFS score =0 at FU^1^ | Not assessed | Not assessed | Not assessed |
| **Larsson 2015** | Europe 2009-2011 | 49.0 years  79.0%  Pre 2018 GL | aMF 200mcg (n=47) QID for 16 weeks | Not assessed | Not assessed | Change in WDS score at FU | Not assessed | Number of dilations |
| **Laserna-Mendieta 2023** | Europe 2015-2021 | 38.2 years  76.8%  Post 2018 GL | PPI (n=764) and TCS (n=66) therapy  Treatment duration not reported | <15 eos/hpf | Not assessed | Symptoms reduction <50% (remission) or ≤50% (response) at FU assessed by DSS | Not assessed | Number of dilations |
| **Lee 2012** | US  Period not reported | 40.0 years  27.0%  Pre 2018 GL | aFLU 880mcg BID or BUD 3mg (BID week 1 + QID weeks 2-6) (n=11) for 6 weeks | Not assessed | Not assessed | Proportion of patients with complete (answer “no”) or partial response (decrease in dysphagia severity >1 level, dysphagia frequency ≥1 level) assessed by MDQ | Not assessed | Number of dilations |
| **Leigh 2019** | US  Period not reported | 39.0 years  64.0%  Pre 2018 GL | PPI (n=580) and TCS (n=133) therapy  Posology and treatment duration not reported | Not assessed | Not assessed | Not assessed | Not assessed | Infection and infestation TEAE |
| **Lingblom 2021** | Europe period not reported | 41.0 years  70.0%  Pre 2018 GL | MF 200mcg (n=20) QID for 8 weeks | <15 eos/hpf | Not assessed | Change in WDS score at FU | Not assessed | Not assessed |
| **Lluncor 2018** | Europe 2000-2014 | 34.6 years  75.4%  Pre 2018 GL | PPI (n=61) therapy  Treatment duration not reported | <15 eos/hpf | Not assessed | Not assessed | Not assessed | Not assessed |
| **Lorenz 2022** | Europe 2006-2020 | 48.0 years  62.0%  Post 2018 GL | BUD ODT/vBUD/ cFLU (n=29) for 96 weeks  Posology not reported | <15 eos/hpf | Change in EREFS at FU | Change in SDI and proportion of patients with >3 reduction in SDI at FU | Not assessed | Not assessed |
| **Lucendo 2011b** | Europe  Period not reported | 34.9 years  81.8%  Pre 2018 GL | FLU 400mcg (n=11) BID for 24 weeks | Not assessed | Not assessed | Change in symptoms at FU assessed by a dysphagia score (0-18) | Not assessed | Not assessed |
| **McMurray 2022** | US 2012-2021 | 33.0 years  67.4%  Post 2018 GL | PPI (n=11) and TCS (N=29) therapy  Posology and treatment duration not reported | <15 eos/hpf | Not assessed | Not assessed | Not assessed | Not assessed |
| **Melgaard 2023** | Europe 2018-2021 | 41.0 years  67.7%  Post 2018 GL | PPI (n=283) therapy for 9.7 weeks  Posology not reported | <15 eos/hpf | Not assessed | Not assessed | Not assessed | Not assessed |
| **Miller 2021** | US 2005-2017 | 44.0 years  52.4%  Pre 2018 GL | TCS therapy (N=10) for a median FU of 4 years | Not assessed | Not assessed | Not assessed | Not assessed | Time to first and second dilation |
| **Molina-Infante 2014** | Europe 2010-2013 | 31.0 years  77.0%  Post 2018 GL | OME 40mg (n=53) BID and FLU 400mcg (n=15) BID for 8 weeks | <5/15 eos/hpf | Not assessed | Clinical remission defined as a reduction in the score ≥50% at FU assessed by DSS | Not assessed | Not assessed |
| **Muftah 2023** | US 2012-2016 | 41.6 years  57.9%  Post 2018 GL | PPI therapy (n=183) BID for 8 weeks | <15 eos/hpf | Not assessed | Not assessed | Not assessed | Not assessed |
| **Navarro 2021** | Europe 2016-2020 | 36.0 years  77.6%  Post 2018 GL | PPI therapy (n=166) QD for 10 weeks and TCS therapy (N=79) for 8 weeks | <5/15 eos/hpf | Change in EREFS score at FU | Not assessed | Not assessed | Not assessed |
| **Nennstiel 2016** | Europe 2013-2015 | 34.0 years  95.0%  Post 2018 GL | Liquid BUD suspension 1mg (n=20) BID for 8 weeks | <15 eos/hpf | Change in EREFS at FU^1^ | Change in SDI and proportion of patients with >3 reduction (complete response) or ≤3 reduction (partial response) in SDI at FU | Not assessed | Not assessed |
| **Nicodème 2013** | US 2009-2012 | 38.0 years  71.0%  Pre 2018 GL | PPI (n=70) and TCS (N=7) therapy Posology and treatment duration not reported | <15 eos/hpf | Not assessed | Not assessed | Not assessed | Not assessed |
| **Okimoto 2021** | Asia 2011-2019 | 47.5 years  82.3%  Pre 2018 GL | PPI therapy (n=149) and TCS therapy (n=23) for 52 weeks  Posology not reported | <15 eos/hpf | Not assessed | Not assessed | Not assessed | Not assessed |
| **Oliva 2022** | Europe 2015-2017 | 13.8 years  60.0%  Pre 2018 GL | PPI therapy (n=296) 2mg/kg QD and TCS therapy (n=102) for 8 weeks  TCS posology not reported | <15 eos/hpf | Not assessed | Not assessed | Not assessed | Not assessed |
| **Philpott 2016a** | Australia 2013-2015 | 36.3 years  81.3%  Pre 2018 GL | ESO 40mg BID(n=107)/ESO 40mg BID+FED (n=56) for 8 weeks and BUD 1mg (n=50) BID for 6 weeks | <5 eos/hpf | Not assessed | Not assessed | Not assessed | Not assessed |
| **Podboy 2016** | US 2010-2015 | 38.9 years  58.5%  Pre 2018 GL | PPI therapy (n=94) for 8 weeks  Posology not reported | <15 eos/hpf | Not assessed | Not assessed | Not assessed | Not assessed |
| **Reed 2018b** | US 2009-2014 | 38.0 years  55.0%  Pre 2018 GL | FLU 880mcg BID or vBUD 1mg BID (n=59) for 8 weeks | <15 eos/hpf | Change in ESS at FU and proportion of patients with 50% improvement in ESS | Change in VAS at FU and proportion of patients with 50% improvement in VAS | Not assessed | Not assessed |
| **Runge 2017** | US 2001-2014 | 35.4 years  62.0%  Pre 2018 GL | FLU 880mcg BID or BUD 1mg BID (n=55) for 8 weeks | <15 eos/hpf | Not assessed | Not assessed | Not assessed | Number of dilations |
| **Sato 2018** | Asia 2016-2018 | 44.0 years  94.1%  Pre 2018 GL | PPI therapy (n=17) for 8 weeks  Posology not reported | <15 eos/hpf | Not assessed | Not assessed | Not assessed | Not assessed |
| **Sawada 2019** | Asia 2010-2018 | 46.0 years  65%  Post 2018 GL | PPI (n=101) and TCS (n=4) therapy for 8 weeks  Posology not reported | <15 eos/hpf | Not assessed | Not assessed | Not assessed | Not assessed |
| **Schlag**  **2014** | Europe 2010-2012 | 35.0 years  93.0%  Pre 2018 GL | FLU 500mcg (n=15) BID for 12 weeks | <1/6/15 eos/hpf (patient-level data) | Not assessed | Change in dysphagia symptoms at FU assessed by questionnaire (score 0-10) | Not assessed | Not assessed |
| **Schupack 2023** | US 2003-2019 | 40.4 years  53.4%  Post 2018 GL | BUD gel 3mg BID (n=62) for 19.4 weeks and BUD capsule 3mg BID (n=41) for 11.8 weeks | <7/15 eos/hpf | Not assessed | Not assessed | Not assessed | Number of dilations |
| **Sia 2023** | US 2013-2021 | Post 2018 GL  Age and %male not reported | PPI therapy (n=176) for 8 weeks  Posology not reported | <10 eos/hpf | Not assessed | Not assessed | Not assessed | Not assessed |
| **Simon 2018** | Europe  Period not reported | 43.0 years  77.8%  Pre 2018 GL | TCS therapy (n=45)  Posology and treatment duration not reported | Not assessed | Not assessed | Not assessed | Not assessed | Infection and infestation TEAE |
| **Stern 2018** | US 2013-2014 | 37.2 years  63.0%  Pre 2018 GL | Not reported | <15 eos/hpf | Not assessed | Not assessed | Not assessed | Not assessed |
| **Thakkar 2022** | US  Period not reported | 45.2 years  58.0%  Post 2018 GL | PPI 40/80mg (n=50) QD for 8 weeks | <5/15 eos/hpf | Change in EREFS score at FU | Not assessed | Not assessed | Number of dilations |
| **Urquhart 2021** | US 2000-2019 | 39.0 years  68.1%  Pre 2018 GL | PPI (n=54) and TCS (n=45) therapy  Posology and treatment duration not reported | <15 eos/hpf | Not assessed | Not assessed | Not assessed | Any side effects leading to discontinuation, number of dilations |
| **Van Rhijn 2015** | Europe 2011-2014 | 43.0 years  66.7%  Pre 2018 GL | FLU 500mcg BID (n=15) for 8 weeks | <15 eos/hpf | Change in EREFS score at FU | Change in dysphagia symptoms assessed with a six-graded Likert scale at FU | Not assessed | Not assessed |

**^1^** Also available for each individual EREFS subscore.

**Abbreviations: aFLU**, aerosolized fluticasone; **aMF**, aerosolized mometasone furoate; **aMF**, aerosolized mometasone furoate; **BID**, bis in die; **BUD ODT**, budesonide orally disintegrated tablet; **BUD**, budesonide; **cFLU**, compounded fluticasone; **CR**, clinical response; **DSS**, Dysphagia Symptoms Score; **EoE**, eosinophilic oesophagitis; **Eos/hpf**, eosinophils/high-power field; **EREFS**, EoE Endoscopic Reference Score; **ESO**, esomeprazole; **FED**, Food Elimination Diet; **FLU ODT**, Fluticasone orally disintegrated tablet; **FLU**, fluticasone; **FU**, follow-up; **GL**, guidelines; **MDQ**, Mayo Dysphagia Questionnaire; **nBUD**, nebulized budesonide; **OME**, omeprazole; **PGA**, Patient Global Assessment; **PPI**, Proton-pump inhibitor; **QD**, quaque die; **RABE**, rabeprazole; **SDI**, Straumann Dysphagia Index; **TCS**, topical corticosteroids; **TEAE**, treatment emergent adverse event; **US**, United States; **VAS**, Visual Analogue Scale; **vBUD**, viscous budesonide; **WDS,** Watson Dysphagia Scale.

Table E3. Main characteristics of observational studies (included in the SLR only) in patients with active EoE

| Study and year | Country and period | Population and definition of active  EoE (Mean age; % male) | Disease management and quality of life |
| --- | --- | --- | --- |
| **Chang 2021a** | US 2019 | 38.7 years  Pre 2018 GL  %male not reported | SDM questionnaire about treatment options and related resource consumption for the therapy |
| **De Rooij 2022** | Europe 2019-2020 | 43.0 years  61.0%  Post 2018 GL | General HRQOL evaluated with the SF-36 in patients treated with both PPI and TCS |
| **Desai 2005** | US 2000-2003 | 55.1 years  80.6%  Post 2018 GL | First and second-line treatment distribution and number of dilations performed during FU in patients treated with both PPI and TCS |
| **Eluri 2020a** | US 2018 | Pre 2018 GL  Mean age and %male not reported | Survey to assess EoE practice patterns of gastroenterologists (i.e. EoE treatments based on practice setting) |
| **Greenberg 2022** | US 2019 | 39.1 years  66.8%  Pre 2018 GL | Response to dilation therapy and post-dilation TEAE |
| **Hewett 2017** | Europe 2013-2014 | 42 years  75.0%  Pre 2018 GL | QoL assessed by SF-36 and EoE-QoL-A in patients treated with both PPI and TCS |
| **Kahn 2015** | Europe  Period not reported | 24.0 years  50.0%  Pre 2018 GL | First-line treatment distribution |
| **Kim 2018** | Asia 2006-2014 | 44.0 years  78.4%  Post 2018 GL | First and second-line treatment distribution |
| **King 2010** | US 2006-2007 | Pre 2018 GL  Mean age and %male not reported | First-line treatment distributions assessed by surveys administered to pediatric and adult gastroenterologists |
| **Kozon 2023** | Europe 2007-2017 | Pre 2018 GL  Mean age and %male not reported | Gender-based differences in complications among patients with EoE |
| **Lin 2018** | US  Period not reported | 42.0 years  50.9%  Pre 2018 GL | First and second-line treatment distributions assessed by telephone survey with patients |
| **Lingblom 2014** | Europe  Period not reported | 43.0 years  85.7%  Pre 2018 GL | First-line treatment distribution |
| **Lucendo 2013** | Europe 2010-2012 | 31.2 years  80.4%  GL not reported | Clinical patterns and overall treatments distribution assessed by surveys administered to adult and pediatric gastroenterologists and allergists |
| **Lucendo 2018** | Europe 2014 | 33.5 years  73.5%  Pre 2018 GL | QoL assessed with EoE-QoL-A in both PPI and TCS patients |
| **Maradey-Romero 2015** | US 2011-2014 | 62.6%  Pre 2018 GL  Mean age not reported | Overall treatments distribution |
| **Menard-Katcher 2013** | US 2010-2011 | 20.5 years  75.0%  Post 2018 GL | QoL assessed by PAGI in both PPI and TCS patients |
| **Murray 2016** | Australia 2012-2013 | 38.0 years  Post 2018 GL  %male not reported | First-line treatment distribution |
| **Nagarajan 2023** | Asia 2019-2021 | 38.0 years  75.0%  Post 2018 GL | First and second line treatment distribution |
| **Ocampo 2023** | US 2020 | 38.2 years  70.7%  Pre and post 2018 (based on diagnosis time) | Overall treatment distribution |
| **Podboy 2019** | US 2003-2006 | 55.0 years  64.8%  Post 2018 GL | First-line treatment distribution, number of dilations and QoL assessed by Likert scale (1-4 score) |
| **Safroneeva 2015** | US and Europe 2013-2014 | 39.1 years  64.3%  Pre 2018 GL | QoL assessed by EoE-QoL-A in patients treated with TCS |
| **Safroneeva 2018** | Europe 2016-2017 | 40.0 years  76.9%  Pre 2018 GL | Overall treatment distribution |
| **Safroneeva 2020** | Europe 2016 | 46.9 years  78.7%  Pre 2018 GL | QoL assessed by EoE-QoL-A in patients treated with both TCS and PPI |
| **Saligram 2014** | US  Period not reported | 33.0 years  90.0%  Pre 2018 GL | Number of dilations |
| **Savarino 2020** | Europe 2020 | 36.6 years  66.7%  Pre 2018 GL | Overall treatment distribution |
| **Schreiner 2022** | Europe  Period not reported | 35.8 years  77.1%  Post 2018 GL | QoL assessed by EoE-QoL-A in patients treated with both TCS and PPI |
| **Schreiner 2023** | Europe 2022 | Pre 2018 GL  Mean age and %male not reported | First-line treatment distribution |
| **Schupack 2021** | US 2000-2017 | 41.6 years  59.7%  Post 2018 GL | Dilations rate at 1,2,5 years and Kaplan-Meier curves for dilation free time during maintenance |
| **Tomomatsu 2013** | Asia 2010-2011 | 48.0 years  70.0%  Post 2018 GL | First and second-line treatment distribution |
| **Tourlamain 2020** | Europe and UAE 2017-2018 | Post 2018 GL  Mean age and %male not reported | First and second-line treatment distribution |
| **Ukleja 2014** | US 2004-2010 | 42.0 years  72.1%  Pre 2018 GL | Post-dilation TEAE |
| **Vermeulen 2018** | Europe 2004-2014 | 38.0 years  79.8%  Post 2018 GL | First-line treatment distribution |

**Abbreviations**: **EoE**, eosinophilic esophagitis; **EoE-QoL-A**, EoE Adult Quality of Life Questionnaire; **FU**, follow-up; **GL**, guidelines; **HRQOL**, health-related quality of life; **PAGI**, Patient Assessment of Upper Gastrointestinal Disorders; **PPI**, proton-pump inhibitor; **QoL**, Quality of Life; **SDM**, Shared Decision Making; **SF-36**, Short Form-36 Health Survey; **TCS**, topical corticosteroid; **TEAE**, treatment emergent adverse event; **UAE**, United Arab Emirates; **US**, United States.

Table E4. Characteristics of patients relapsed after achieving remission post TCS induction

| Relapse assessed | Study | Treatment description | Patients | Relapse | Time to relapse | Complication? |
| --- | --- | --- | --- | --- | --- | --- |
| **After TCS cessation** | Dellon 2022a | Subjects with a histologic response (<15 eos/hpf) after 8 weeks of BUD (1 mg BID) or FLU (880 mcg BID) entered an observation phase in which treatment was discontinued and symptoms were monitored. | 58 (33 BUD + 25 FLU) | Symptoms recurrence in 33 patients (57%) | Median 244 days (35 weeks)  BUD 263 vs. FLU 224 days (p=0.91) | 78% of subjects had histologic relapse (>15 eos/hpf) at the time of symptom recurrence or at the 1 year follow-up endoscopy. During the observation phase, there were no food impactions, and no subjects had esophageal candidiasis on their final endoscopy |
|  | Greuter 2017 | Patients who achieved long-lasting (≥6 months) clinical, endoscopic, and histological (=deep) remission with BUD or FLU (1 mg BID for 2-4 weeks, then then 0.25 mg BID), treatment was stopped after a median time of 104.7 weeks (IQR 65.5─176.6) | 33 | Reappearance of EoE-attributed symptoms in 26 patients (78.8%) | Median 22.4 weeks (95% CI 5.1 to 39.7) | In 8 of the 26 patients with a clinical relapse (30.8%), EGD was carried out. Histologically active disease was confirmed in 6 of these 8 patients (biopsies were not available for 1 patient; another patient showed only 8 eos/hpf despite experiencing clinical and endoscopic relapse). In all patients with EoE relapse, treatment with TCS was reinitiated with a re-achievement of disease remission. No severe complications, such as food impaction or strictures, were reported |
|  | Helou 2008 | Patients with initial histologic remission (<15 eos/hpf) after 6 weeks of FLU (880 mcg), who were contacted by email after a medina follow-up of 3.3 years | 32 | 29 patients (91%) reported recurrent dysphagia occurring at a mean of 8.8 months after the treatment was complete | Mean time 8.8 months (38 weeks) | 22 patients (69%) required a repeated treatment course with swallowed FLU. 12 patients (38%) required 2+ repeated treatments, and 4 patients (13%) required four 4+ repeated treatments. 15 (47%) patients reported just as needed use, usually until the symptoms improved, not taking another 6-wk course. Six patients (19%) required a course of oral steroids. No patients reported regular daily maintenance steroid use.  9 patients (28%) experienced subsequent food impactions at a mean of 2.3 times (range 1–5 times) during the 3-yr-follow-up period. Seven patients (22%) required endoscopic dilation at a mean of 1.8 times (range 1–3). |
| **After TCS dose reduction** | Eluri 2017 | Patients with initial histologic response (<15 eos/hpf) after an 8-week course of  TCS (BUD 1-2 mg BID, FLU 880 mcg BID), and who were subsequently maintained on steroid therapy for ≥75% of the follow-up time (dose reduction up to 50-70%). | 33 (25 BUD + 8 FLU) with 2+ subsequent endoscopies during follow-up | Histologic relapse (>15 eos/hpf) in 26 patients with at least 2 subsequent endoscopies (79%) | Median 18.5 months (80 weeks) | Patients who were maintained on a higher dose (>1 g BUD or >880 mcg FLU) had lower risk of loss of treatment response compared to those with dose reduced (≤1 g BUD or ≤880 mcg FLU) OR: 0.10 (95% CI: 0.01 to 0.90) |
|  | Greuter 2021 | Patients with initial histologic response (<15 eos/hpf) after TCS (mean treatment duration 1 year, mean daily dose 1.55 mg), and who were subsequently maintained on steroid therapy with a mean daily dose of 0.78 mg^1^ Patients were stratified in low-dose (<0.5 mg per day) and high-dose (>0.5 mg per day) | 82 (22 BUD + 60 FLU)  58 low-dose + 24 high-dose | Histologic relapse (>15 eos/hpf) in 55 patients (67%)  42 (72%) low-dose and 13 (54%) high-dose (p=0.178) | Median 1.1 years (57.2 weeks)  1.0 years low-dose and 1.8 years high-dose (p=0.030) | Bolus impactions occurred in 3 patients in the low-dose group. Overall need for endoscopic bolus removal under TCS was significantly reduced compared with before treatment  (4% vs 13%, P<0.025). Rates of and time to stricture formation were not significantly different between the 2 groups (43% vs 63%, p=NS). |
|  | Strauman 2011 | Patients with initial histologic remission (<5 eos/hpf) after 2 weeks of high-dose BUD (2 mg), who were subsequently maintained on BUD 0.5 g daily | 14 | Clinical relapse in 5 patients (35.7%) at week 50 | Median not reached | NA |
|  | Strauman 2020 | Patients with initial histologic (<15 eos/hpf) and clinical remission after 6 weeks of BUD ODT (2 mg), who were subsequently maintained on BUD ODT 0.5 g BID (arm 1) | 68 | Histologic relapse (>15 eos/hpf) in 9 patients (13.2%) at week 48 | Median not reached | No bolus impactions occurred |
| **Same dose used to achieve the first remission** | Dellon 2022a | Patients with initial histologic (<6 eos/hpf) and clinical remission after 12 weeks of BUD (2 mg BID), who were subsequently maintained on the same dose | 25 | Histologic relapse (>15 eos/hpf) in 6 patients (24%) after 36 weeks | Not reported | 7.7% of patients were hospitalized due to TEAE |
|  | Strauman 2020 | Patients with initial histologic (<15 eos/hpf) and clinical remission after 6 weeks of BUD ODT (2 mg), who were subsequently maintained on the same dose (arm 2) | 68 | Histologic relapse (>15 eos/hpf) in 7 patients (10.3%) at week 48 | Median not reached | No bolus impactions occurred |

**Abbreviations**: **BUD**: budesonide, **EoE**, eosinophilic esophagitis; **FLU**: fluticasone; **TCS**, topical corticosteroid; **TEAE**, treatment emergent adverse event;

1 Estimated using Wan et al. formulas based on median dose at baseline visit 500 mcg (IQR 500-2000, range 220-6000) and median dose during follow-up 500 mcg (IQR 500-880, range 220-2250);

# References

1. Albert D, Heifert TA, Min SB et al. Comparisons of Fluticasone to Budesonide in the Treatment of Eosinophilic Esophagitis. Dig Dis Sci 2016. 10.1007/s10620-016-4110-9
2. Alexander JA, Jung KW, Arora AS, et al. Swallowed Fluticasone Improves Histologic but Not Symptomatic Response of Adults With Eosinophilic Esophagitis. Clin Gastroenterol Hepatol 2012. 10.1016/j.cgh.2012.03.018
3. Alexander R, Alexander JA, Akambase J, et al. Proton Pump Inhibitor Therapy in Eosinophilic Esophagitis: Predictors of Nonresponse. Digestive diseases and sciences 2021. 10.1007/s10620-020-06633-4
4. Bergquist H, Larsson H, Johansson L, et al. Dysphagia and quality of life may improve with mometasone treatment in patients with eosinophilic esophagitis: A pilot study. Otolaryngol Head Neck Surg 2011. 10.1177/0194599811409857
5. Bohm M, Jacobs JW, Gupta A, et al. Most children with eosinophilic esophagitis have a favorable outcome as young adults. Diseases of the esophagus 2017. 10.1111/dote.12454
6. Bon L, Safroneeva E, Bussmann C, et al. Close follow-up is associated with fewer stricture formation and results in earlier detection of histological relapse in the long-term management of eosinophilic esophagitis. United European gastroenterology journal 2022. 10.1002/ueg2.12216
7. Carlson DA, Hirano I, Zalewski A, et al. Improvement in Esophageal Distensibility in Response to Medical and Diet Therapy in Eosinophilic Esophagitis. Clin Transl Gastroenterol 2017. 10.1038/ctg.2017.47
8. Chang JW, Rubenstein JH, Mellinger JL, et al. Motivations, Barriers, and Outcomes of Patient-Reported Shared Decision Making in Eosinophilic Esophagitis. Dig Dis Sci 2021. 10.1007/s10620-020-06438-5
9. De Rooij WE, Evertsz' FB, Lei A, et al. General Well-being and Coping Strategies in Adult Eosinophilic Esophagitis Patients. J Neurogastroenterology Motil 2022. 10.5056/jnm21143
10. DeLay K, Tappata M, Huang KZ, et al. Long-term continued proton pump inhibitor use is common in patients diagnosed with eosinophilic oesophagitis despite failure of histologic response: Data from a two-centre study. GastroHep 2020. 10.1002/ygh2.432
11. Dellon ES, Sheikh A, Speck O, et al. Viscous topical is more effective than nebulized steroid therapy for patients with eosinophilic esophagitis. Gastroenterology 2012. 10.1053/j.gastro.2012.04.049
12. Dellon ES, Rusin S, Gebhart JH, et al. Utility of a Noninvasive Serum Biomarker Panel for Diagnosis and Monitoring of Eosinophilic Esophagitis: A Prospective Study. The American journal of gastroenterology 2015. 10.1038/ajg.2015.57
13. Dellon ES, Katzka DA, Collins MH, et al. Budesonide Oral Suspension Improves Symptomatic, Endoscopic, and Histologic Parameters Compared With Placebo in Patients With Eosinophilic Esophagitis. Gastroenterology 2017. 10.1053/j.gastro.2016.11.021
14. Dellon ES, Katzka DA, Collins MH, et al. Safety and Efficacy of Budesonide Oral Suspension Maintenance Therapy in Patients With Eosinophilic Esophagitis. Clinical gastroenterology and hepatology Association 2019. 10.1016/j.cgh.2018.05.051
15. Dellon ES, Woosley JT, Arrington A, et al. Efficacy of Budesonide vs Fluticasone for Initial Treatment of Eosinophilic Esophagitis in a Randomized Controlled Trial. Gastroenterology 2019. 10.1053/j.gastro.2019.03.014
16. Dellon ES, Woosley JT, Arrington A, et al. Rapid Recurrence of Eosinophilic Esophagitis Activity After Successful Treatment in the Observation Phase of a Randomized, Double-Blind, Double-Dummy Trial. Clin Gastroenterol Hepatol 2020. 10.1016/j.cgh.2019.08.050
17. Dellon ES, Collins MH, Katzka DA, et al. Long-Term Treatment of Eosinophilic Esophagitis With Budesonide Oral Suspension. Clin Gastroenterol Hepatol 2022. 10.1016/j.cgh.2021.06.020
18. Dellon ES, Lucendo AJ, Schlag C, et al. Fluticasone Propionate Orally Disintegrating Tablet (APT-1011) for Eosinophilic Esophagitis: Randomized Controlled Trial. Clin Gastroenterol Hepatol 2022. 10.1016/j.cgh.2022.02.013
19. Desai TK, Stecevic V, Chang CH, et al. Association of eosinophilic inflammation with esophageal food impaction in adults. Gastrointest Endosc 2005. 10.1016/S0016-5107(05)00313-5
20. Eluri S, Runge TM, Hansen J, et al. Diminishing Effectiveness of Long-Term Maintenance Topical Steroid Therapy in PPI Non-Responsive Eosinophilic Esophagitis. Clin Transl Gastroenterol . 10.1038/ctg.2017.27
21. Eluri S, Selitsky SR, Perjar I, et al. Clinical and Molecular Factors Associated With Histologic Response to Topical Steroid Treatment in Patients With Eosinophilic Esophagitis. Clinical gastroenterology and hepatology 2019. 10.1016/j.cgh.2018.09.005
22. Eluri S, Iglesia EGA, Massaro M, et al. Practice patterns and adherence to clinical guidelines for diagnosis and management of eosinophilic esophagitis among gastroenterologists. Dis Esophagus 2020. 10.1093/dote/doaa025
23. Eluri S, Tappata M, Huang KZ, et al. Distal esophagus is the most commonly involved site for strictures in patients with eosinophilic esophagitis. Dis Esophagus 2020. 10.1093/dote/doz088
24. Francis DL, Foxx-Orenstein A, Arora AS, et al. Results of ambulatory pH monitoring do not reliably predict response to therapy in patients with eosinophilic oesophagitis. Aliment Pharmacol Ther 2012. 10.1111/j.1365-2036.2011.04922.x
25. Frandsen LT, Westmark S, Melgaard D, et al. Effectiveness of PPI treatment and guideline adherence in 236 patients with eosinophilic oesophagitis-Results from the population-based DanEoE cohort shows a low complication rate. United European gastroenterology journal 2021. 10.1002/ueg2.12146
26. Frazzoni M, Penagini R, Frazzoni L, et al. Role of Reflux in the Pathogenesis of Eosinophilic Esophagitis: Comprehensive Appraisal With Off- and On PPI Impedance-pH Monitoring. The American journal of gastroenterology 2019. 10.14309/ajg.0000000000000379
27. Frazzoni M, Frazzoni L, De Bortoli N, et al. Response of eosinophilic oesophagitis to proton pump inhibitors is associated with impedance-pH parameters implying anti-reflux mechanism of action. Aliment Pharmacol Ther 2021. 10.1111/apt.16371
28. Fujiwara Y, Hashimoto A, Uemura R, et al. Optimal Biopsy Protocol to Evaluate Histological Effectiveness of Proton Pump Inhibitor Therapy in Patients with Eosinophilic Esophagitis. Digestion 2019. 10.1159/000494253
29. Yasuhiro Fujiwara, Akinari Sawada, Masaki Ominami, Shusei Fukunaga, Koji Otani, Shuhei Hosomi, Yasuaki Nagami, Koichi Taira, and Fumio Tanaka. Responses of Proton Pump Inhibitors and Potassium-Competitive Acid Blockers According to Outcomes of Symptom, Endoscopy, and Histology in Patients With Eosinophilic Esophagitis. J Clin Gastroenterol 2023. 10.1097/MCG.0000000000001869
30. Ghisa M, Laserra G, Marabotto E, et al. Achalasia and Obstructive Motor Disorders Are Not Uncommon in Patients With Eosinophilic Esophagitis. Clin Gastroenterol Hepatol 2021. 10.1016/j.cgh.2020.07.056
31. Goldin AH, Muftah M, Mangla S, et al. Assessment of the clinical and allergy profiles of PPI responsive and non-responsive eosinophilic esophagitis. Dis Esophagus 2022. 10.1093/dote/doac098
32. Gomez-Torrijos E, García-Rodríguez R, Castro-Jiménez A, et al. The efficacy of step-down therapy in adult patients with proton pump inhibitor-responsive oesophageal eosinophilia. Aliment Pharmacol Ther . 10.1111/apt.13496
33. Greenberg S, Chang NC, Corder ZR, et al. Dilation-predominant approach versus routine care in patients with difficult-to-treat eosinophilic esophagitis: A retrospective comparison. Endoscopy 2022. 10.1055/a-1493-5627
34. Greuter T, Bussmann C, Safroneeva E, et al. Long-Term Treatment of Eosinophilic Esophagitis With Swallowed Topical Corticosteroids: Development and Evaluation of a Therapeutic Concept. The American journal of gastroenterology 2017. 10.1038/ajg.2017.202
35. Greuter T, Godat A, Ringel A, et al. Effectiveness and Safety of High- vs Low-Dose Swallowed Topical Steroids for Maintenance Treatment of Eosinophilic Esophagitis: A Multicenter Observational Study. Clin Gastroenterol Hepatol 2021. 10.1016/j.cgh.2020.08.027
36. Helou EF, Simonson J, Arora AS. 3-Yr-follow-up of topical corticosteroid treatment for eosinophilic esophagitis in adults. Am J Gastroenterol 2008. 10.1111/j.1572-0241.2008.01989.x
37. Hewett R, Alexakis C, Farmer AD, et al. Effects of eosinophilic oesophagitis on quality of life in an adult UK population: A case control study. Dis Esophagus 2017. 10.1111/dote.12524
38. Hirano I, Safroneeva E, Roumet MC, et al. Randomised clinical trial: the safety and tolerability of fluticasone propionate orally disintegrating tablets versus placebo for eosinophilic oesophagitis. Aliment Pharmacol Ther 2020. 10.1111/apt.15670
39. Hirano I, Collins MH, Katzka DA, et al. Budesonide Oral Suspension Improves Outcomes in Patients With Eosinophilic Esophagitis: Results from a Phase 3 Trial. Clin Gastroenterol Hepatol 2022. 10.1016/j.cgh.2021.04.022
40. Kahn J, Bussmann C, Beglinger C, et al. Exercise-induced chest pain: an atypical manifestation of eosinophilic esophagitis. The American journal of medicine 2015. 10.1016/j.amjmed.2014.08.007
41. Ketchem CJ, Reed CC, Stefanadis Z, et al. Treatment with compounded fluticasone suspension improves the clinical, endoscopic, and histologic features of eosinophilic esophagitis. Diseases of the esophagus 2021. 10.1093/dote/doaa120
42. Ketchem CJ, Ocampo AA, Xue Z, et al. Higher Body Mass Index Is Associated With Decreased Treatment Response to Topical Steroids in Eosinophilic Esophagitis. Clin Gastroenterol Hepatol 2023. 10.1016/j.cgh.2022.11.004
43. Kia L, Nelson M, Zalewski A, et al. Oral delivery of fluticasone powder improves esophageal eosinophilic inflammation and symptoms in adults with eosinophilic esophagitis. Dis Esophagus 2018. 10.1093/dote/doy098
44. Kim GH, Jung KW, Jung HY, et al. Diagnostic trends and clinical characteristics of eosinophilic esophagitis: A Korean, single-center database study. J Neurogastroenterology Motil 2018. 10.5056/jnm17047
45. Kim SJ, Park MI, Kim GH, et al. Practice patterns for eosinophilic esophagitis patients in busan and gyeongnam: A korean multicenter database study. J Neurogastroenterology Motil 2021. 10.5056/JNM20069
46. King J, Khan S. Eosinophilic esophagitis: perspectives of adult and pediatric gastroenterologists. Digestive diseases and sciences 2010. 10.1007/s10620-009-0801-9
47. Kon T, Abe Y, Sasaki Y, et al. Clinical features of esophageal eosinophilia according to endoscopic phenotypes. Intern Med 2020. 10.2169/internalmedicine.4447-20
48. Kozon I, Frandsen LT, Izgi B, et al. No gender differences in patients with eosinophilic oesophagitis. Dan Med J 2023. 17;70(2):A06220393
49. Kuzumoto T, Tanaka F, Sawada A, et al. Vonoprazan shows efficacy similar to that of proton pump inhibitors with respect to symptomatic, endoscopic, and histological responses in patients with eosinophilic esophagitis. Esophagus 2021. 10.1007/s10388-020-00783-0
50. Larsson H, Bergman K, Finizia C, et al. Dysphagia and health-related quality of life in patients with eosinophilic esophagitis: a long-term follow-up. European archives of oto-rhino-laryngology 2015. 10.1007/s00405-015-3696-4
51. Laserna-Mendieta EJ, Navarro P, Casabona-Francés S, et al. Differences between childhood- and adulthood-onset eosinophilic esophagitis: An analysis from the EoE connect registry. Dig Liver Dis 2023. 10.1016/j.dld.2022.09.020
52. Lee J, Huprich J, Kujath C, et al. Esophageal diameter is decreased in some patients with eosinophilic esophagitis and might increase with topical corticosteroid therapy. Clinical gastroenterology and hepatology 2012. 10.1016/j.cgh.2011.12.042
53. Leigh LY, Spergel JM. An in-depth characterization of a large cohort of adult patients with eosinophilic esophagitis. Ann Allergy Asthma Immunol 2019. 10.1016/j.anai.2018.09.452
54. Lin SK, Zhang S, Kalra N, et al. Challenges in managing patients referred for eosinophilic esophagitis: A telephone survey and retrospective review. Allergy and asthma proceedings 2018. 10.2500/aap.2018.39.4163
55. Lingblom C, Bergquist H, Johnsson M, et al. Topical corticosteroids do not revert the activated phenotype of eosinophils in eosinophilic esophagitis but decrease surface levels of CD18 resulting in diminished adherence to ICAM-1, ICAM-2, and endothelial cells. Inflammation 2014. 10.1007/s10753-014-9926-x
56. Lingblom C, Albinsson S, Johansson L, et al. Patient-Reported Outcomes and Blood-Based Parameters Identify Response to Treatment in Eosinophilic Esophagitis. Digestive diseases and sciences 2021. 10.1007/s10620-020-06368-2
57. Lluncor M, Pedrosa M, Cancelliere N, et al. Molecular Sensitization Profile According to Proton Pump Inhibitor Response in Patients With Esophageal Eosinophilia. Journal of investigational allergology & clinical immunology 2018. 10.18176/jiaci.0289
58. Lorenz NJ, Link A, Czapiewski P,et al. Eosinophilic esophagitis: Comparison of clinical, endoscopic and histological scoring systems. Z Gastroenterol 2022. 10.1055/a-1855-1974
59. Lucendo AJ, De Rezende LC, Jiménez-Contreras S, et al. Montelukast was inefficient in maintaining steroid-induced remission in adult eosinophilic esophagitis. Digestive diseases and sciences 2011. 10.1007/s10620-011-1775-y
60. Lucendo AJ, Arias A, Molina-Infante J, et al. Diagnostic and therapeutic management of eosinophilic oesophagitis in children and adults: results from a Spanish registry of clinical practice. Digestive and liver disease 2013. 10.1016/j.dld.2013.01.013
61. Lucendo AJ, Arias-González L, Molina-Infante J, et al. Determinant factors of quality of life in adult patients with eosinophilic esophagitis. United Eur Gastroenterol J 2018. 10.1177/2050640617707095
62. Maradey-Romero C, Prakash R, Lewis S, et al. The 2011-2014 prevalence of eosinophilic oesophagitis in the elderly amongst 10 million patients in the United States. Alimentary pharmacology & therapeutics 2015. 10.1111/apt.13171
63. McMurray JC, St Clair B, Spriet SW, et al. Outcomes of eosinophilic esophagitis in patients managed in a multidisciplinary clinic. Allergy Asthma Proc 2022. 10.2500/aap.2022.43.210102
64. Melgaard D, Byrholdt Hansen A, Pedersen C, Sandholm E, Bech Hansen T, Tegtmeier Frandsen L, Lund Krarup A. An improved guideline adherence and PPI efficacy has been accompanied by a decrease in diagnostic delay, and strictures before diagnosis of eosinophilic esophagitis in the North Denmark Region - retrospective registry study of the DanEoE cohorts. Clinics and Research in Hepatology and Gastroenterology 2023. 10.1016/j.clinre.2023.102159
65. Menard-Katcher P, Marks KL, Liacouras CA, et al. The natural history of eosinophilic oesophagitis in the transition from childhood to adulthood. Alimentary pharmacology & therapeutics 2013. 10.1111/apt.12119
66. Miehlke S, Hruz P, Vieth M, et al. A randomised, double-blind trial comparing budesonide formulations and dosages for short-term treatment of eosinophilic oesophagitis. Gut 2016. 10.1136/gutjnl-2014-308815
67. Miehlke S, Schlag C, Lucendo AJ, et al. Budesonide orodispersible tablets for induction of remission in patients with active eosinophilic oesophagitis: A 6-week open-label trial of the EOS-2 Programme. United Eur Gastroenterol J 2022. 10.1002/ueg2.12220
68. Miller D, Mago S, Birk JW, et al. Obesity reduces the requirement for subsequent esophageal stricture dilation in adults with eosinophilic esophagitis. Esophagus 2021. 10.1007/s10388-021-00838-w
69. Moawad FJ, Veerappan GR, Dias JA, et al. Randomized controlled trial comparing aerosolized swallowed fluticasone to esomeprazole for esophageal eosinophilia. The American journal of gastroenterology 2013. 10.1038/ajg.2012.443
70. Molina-Infante J, Rivas MD, Hernandez-Alonso M, et al. Proton pump inhibitor-responsive oesophageal eosinophilia correlates with downregulation of eotaxin-3 and Th2 cytokines overexpression. Alimentary pharmacology & therapeutics 2014. 10.1111/apt.12914
71. Muftah M, Barshop K, Redd WD, et al. Baseline Peripheral Eosinophil Count Independently Predicts Proton Pump Inhibitor Response in Eosinophilic Esophagitis. J Clin Gastroenterol 2023. 10.1097/MCG.0000000000001845
72. Murray I, Joyce S, Palmer J, et al. Incidence and features of eosinophilic esophagitis in dysphagia: A prospective observational study. Scand J Gastroenterol 2016. 10.3109/00365521.2015.1093166
73. Nagarajan KV, Krishnamurthy AN, Yelsangikar A, et al. Does eosinophilic esophagitis exist in India? Indian J Gastroenterol 2023. 10.1007/s12664-022-01313-9
74. Navarro P, Laserna-Mendieta EJ, Guagnozzi D, et al. Proton pump inhibitor therapy reverses endoscopic features of fibrosis in eosinophilic esophagitis. Digestive and liver disease 2021. 10.1016/j.dld.2021.05.025
75. Nennstiel S, Bajbouj M, Becker V, et al. High-resolution manometry in patients with eosinophilic esophagitis under topical steroid therapy-a prospective observational study (HIMEOS-study). Neurogastroenterol Motil 2016. 10.1111/nmo.12753
76. Nicodème F, Hirano I, Chen J, et al. Esophageal distensibility as a measure of disease severity in patients with eosinophilic esophagitis. Clinical gastroenterology and hepatology 2013. 10.1016/j.cgh.2013.03.020
77. Ocampo AA, Dellon ES. Worsened Fibrostenotic Outcomes in Eosinophilic Esophagitis Patients Due to COVID-19-Related Endoscopy Cancellations. Dig Dis Sci 2023. 10.1007/s10620-022-07610-9
78. Okimoto E, Ishimura N, Ishihara S. Clinical Characteristics and Treatment Outcomes of Patients with Eosinophilic Esophagitis and Eosinophilic Gastroenteritis. Digestion 2021. 10.1159/000511588
79. Oliva S Dias JA, Rea F, Malamisura M, et al. Characterization of Eosinophilic Esophagitis From the European Pediatric Eosinophilic Esophagitis Registry (pEEr) of ESPGHAN. Journal of pediatric gastroenterology and nutrition 2022. 10.1097/MPG.0000000000003530
80. Peterson KA, Thomas KL, Hilden K, et al. Comparison of esomeprazole to aerosolized, swallowed fluticasone for eosinophilic esophagitis. Digestive diseases and sciences 2010. 10.1007/s10620-009-0859-4
81. Philpott H, Nandurkar S, Royce SG, et al. A prospective open clinical trial of a proton pump inhibitor, elimination diet and/or budesonide for eosinophilic oesophagitis. Aliment Pharmacol Ther 2016. 10.1111/apt.13576
82. Podboy A, Katzka DA, Enders F, et al. Oesophageal narrowing on barium oesophagram is more common in adult patients with eosinophilic oesophagitis than PPI-responsive oesophageal eosinophilia. Alimentary pharmacology & therapeutics 2016. 10.1111/apt.13601
83. Podboy AJ, Lavey C, Mara K, et al. Eosinophilic Esophagitis Is Rarely Continually Symptomatic 10 Years After an Initial Treatment Course in Adults. Digestive diseases and sciences 2019. 10.1007/s10620-019-05636-0
84. Reed C.C. and Fan C. and Koutlas N. and Stefanadis Z. and Eluri S. and Shaheen N.J. and Dellon E.S.. Compounded Oral Viscous Budesonide is Effective and Provides a Durable Response in Eosinophilic Esophagitis. J. Gastroenterol. Hepatol. Res. 2018. 10.17554/j.issn.2224-3992.2018.07.750
85. Runge TM, Eluri S, Woosley JT, et al. Control of inflammation decreases the need for subsequent esophageal dilation in patients with eosinophilic esophagitis. Dis Esophagus 2017. 10.1093/dote/dox042
86. Safroneeva E, Coslovsky M, Kuehni CE, et al. Eosinophilic oesophagitis: Relationship of quality of life with clinical, endoscopic and histological activity. Aliment Pharmacol Ther 2015. 10.1111/apt.13370
87. Safroneeva E, Saner C, Rossel JB, et al. Cohort Profile: The Swiss Eosinophilic Esophagitis Cohort Study (SEECS). Inflamm Intest Dis 2018. 10.1159/000486131
88. Safroneeva E, Hafner D, Kuehni CE, et al. Systematic Assessment of Adult Patients' Satisfaction with Various Eosinophilic Esophagitis Therapies. International archives of allergy and immunology 2020. 10.1159/000504846
89. Saligram S, McGrath K. The safety of a strict wire-guided dilation protocol for eosinophilic esophagitis. Eur J Gastroenterol Hepatol 2014. 10.1097/MEG.0000000000000103
90. Sato H, Honma T, Nozawa Y, et al. Eosinophilic esophagitis in Japanese patients: A mild and slow-progressing disorder. PloS one 2018. 10.1371/journal.pone.0206621
91. Savarino EV, Iovino P, Santonicola A, et al. Clinical and psychological impact of COVID-19 infection in adult patients with eosinophilic gastrointestinal disorders during the SARS-CoV-2 outbreak. J Clin Med 2020. 10.3390/JCM9062011
92. Sawada A, Hashimoto A, Uemura R, et al. Association between endoscopic findings of eosinophilic esophagitis and responsiveness to proton pump inhibitors. Endosc Int Open 2019. 10.1055/a-0859-7276
93. Schlag C, Pfefferkorn S, Brockow K, et al. Serum eosinophil cationic protein is superior to mast cell tryptase as marker for response to topical corticosteroid therapy in eosinophilic esophagitis. Journal of clinical gastroenterology 2014. 0.1097/01.mcg.0000436439.67768.8d
94. Schreiner P, Safroneeva E, Rossel JB, et al. Sex Impacts Disease Activity But Not Symptoms or Quality of Life in Adults With Eosinophilic Esophagitis. Clin Gastroenterol Hepatol 2022. 10.1016/j.cgh.2021.11.009
95. Schreiner P, Balcar L, Schlager H, et al. Management of suspected and known eosinophilic esophagitis—a nationwide survey in Austria. Wien Klin Wochenschr 2023. 10.1007/s00508-023-02198-0
96. Schupack DA, Ravi K, Geno DM, et al. Effect of Maintenance Therapy for Eosinophilic Esophagitis on Need for Recurrent Dilation. Digestive diseases and sciences 2021. 10.1007/s10620-020-06192-8
97. Schupack DA, Johnson K, Akambase JA, et al. Histologic response to steroids in eosinophilic esophagitis is dependent on delivery compound. Dis Esophagus 2023. 10.1093/dote/doac040
98. Sia T, Cunningham E, Miller M, et al. Food elimination diet is a viable alternative therapy for eosinophilic esophagitis responsive to proton pump inhibitors. BMC Gastroenterol 2023. 10.1186/s12876-023-02703-9
99. Simon D, Page B, Vogel M, et al. Evidence of an abnormal epithelial barrier in active, untreated and corticosteroid-treated eosinophilic esophagitis. Allergy 2018. 10.1111/all.13244
100. Stern E, Taft T, Zalewski A, et al. Prospective assessment of disease-specific quality of life in adults with eosinophilic esophagitis. Diseases of the esophagus 2018. 10.1093/dote/dox128
101. Straumann A, Conus S, Degen L, et al. Budesonide is effective in adolescent and adult patients with active eosinophilic esophagitis. Gastroenterology 2010. 10.1053/j.gastro.2010.07.048
102. Straumann A, Conus S, Degen L, et al. Long-term budesonide maintenance treatment is partially effective for patients with eosinophilic esophagitis. Clinical gastroenterology and hepatology 2011. 10.1016/j.cgh.2011.01.017
103. Straumann A, Lucendo AJ, Miehlke S, et al. Budesonide Orodispersible Tablets Maintain Remission in a Randomized, Placebo-Controlled Trial of Patients With Eosinophilic Esophagitis. Gastroenterology 2020. 10.1053/j.gastro.2020.07.039
104. Thakkar KP, Fowler M, Keene S, et al. Long-term efficacy of proton pump inhibitors as a treatment modality for eosinophilic esophagitis. Digestive and liver disease 2022. 10.1016/j.dld.2022.03.006
105. Tomomatsu Y, Yoshino J, Inui K, et al. Clinical features of eosinophilic esophagitis: ten Japanese cases. Digestive endoscopy 2013. 10.1111/j.1443-1661.2012.01340.x
106. Tourlamain G, Garcia-Puig R, Gutiérrez-Junquera C, et al. Differences in Management of Eosinophilic Esophagitis in Europe: An Assessment of Current Practice. Journal of pediatric gastroenterology and nutrition 2020. 10.1097/MPG.0000000000002672
107. Ukleja A, Shiroky J, Agarwal A, et al. Esophageal dilations in eosinophilic esophagitis: A single center experience. World J Gastroenterol 2014. 10.3748/wjg.v20.i28.9549
108. Urquhart SA, Quinn KP, Ravi K, et al. The Clinical Characteristics and Treatment Outcomes of Concomitant Eosinophilic Esophagitis and Inflammatory Bowel Disease. Crohn's Colitis 360 2021. 10.1093/crocol/otab018
109. Van Rhijn BD, Verheij J, Van Den Bergh Weerman MA, et al. Histological Response to Fluticasone Propionate in Patients with Eosinophilic Esophagitis is Associated with Improved Functional Esophageal Mucosal Integrity. Am J Gastroenterol 2015. 10.1038/ajg.2015.247
110. Vermeulen BD, Bogte A, Verhagen MA, et al. Management of eosinophilic esophagitis in daily clinical practice. Diseases of the esophagus 2018. 10.1093/dote/dox119

# Additional Material 5

Figure E1. Forest plot of histologic response (≤15 eos/hpf) in patients treated with PPI


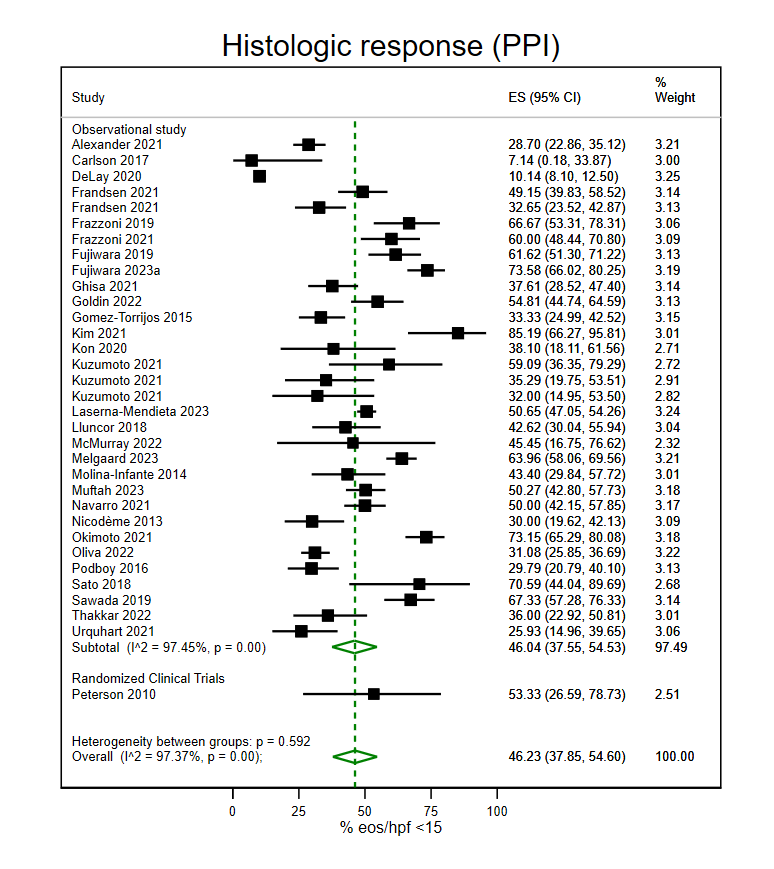


Figure E2. Forest plot of histologic response (≤5-7 eos/hpf) in patients treated with PPI


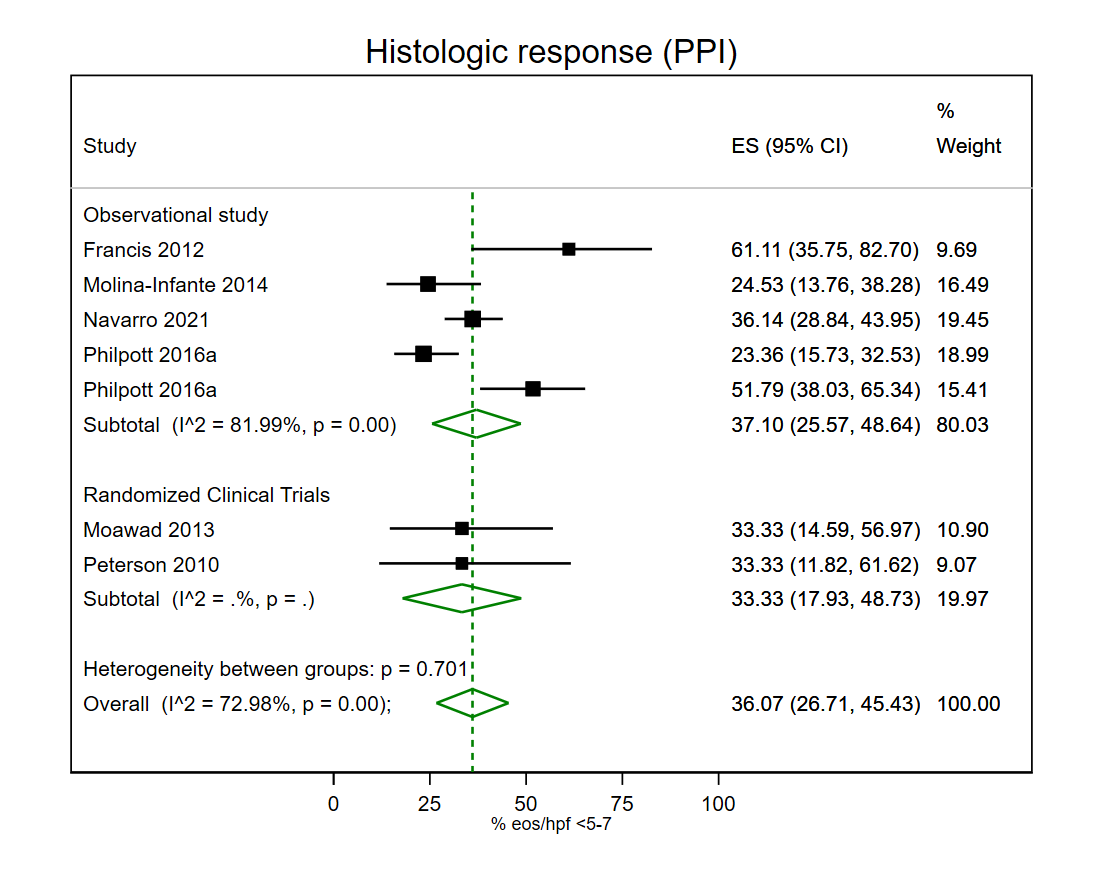


Figure E3. Forest plot of histologic response (≤1 eos/hpf) in patients treated with PPI


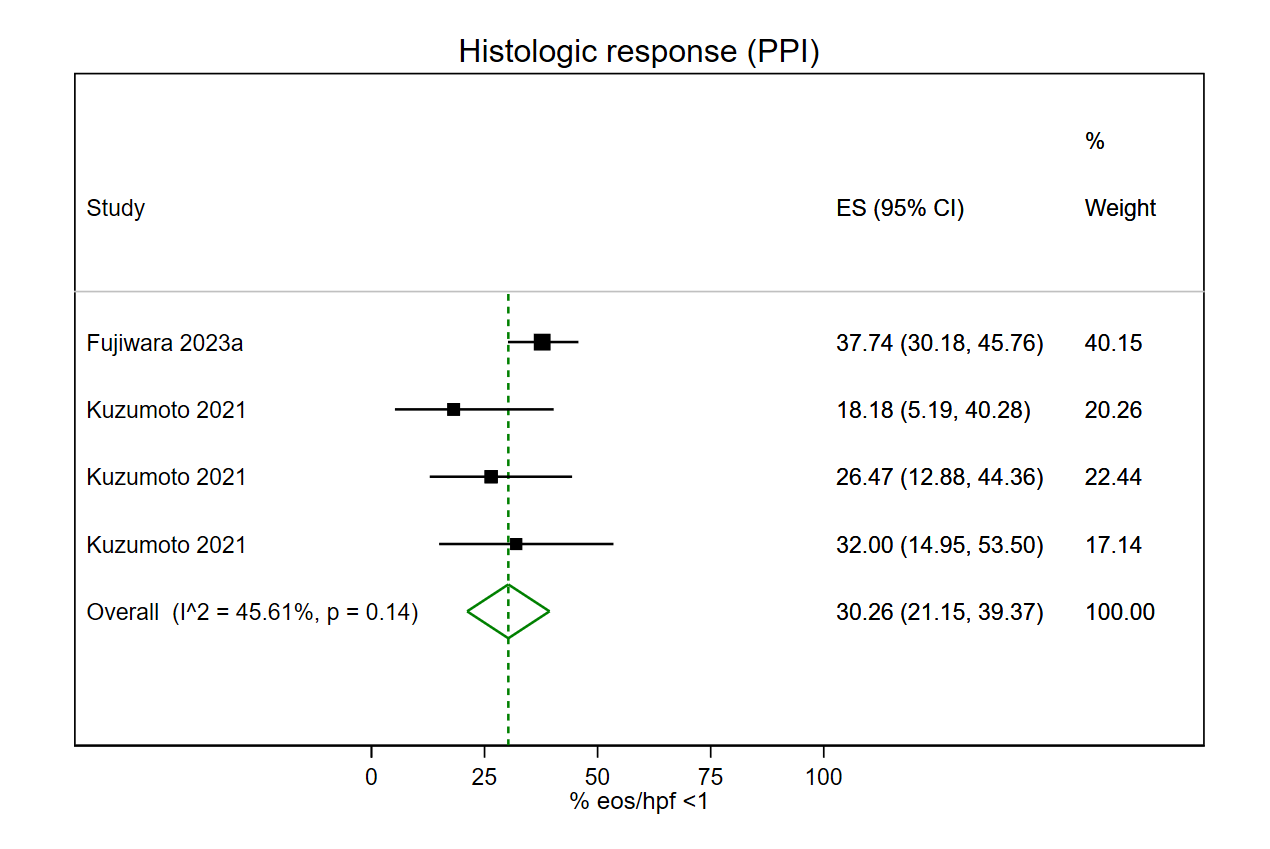


Figure E4. Forest plot of histologic response (≤15 eos/hpf) in patients treated with TCS


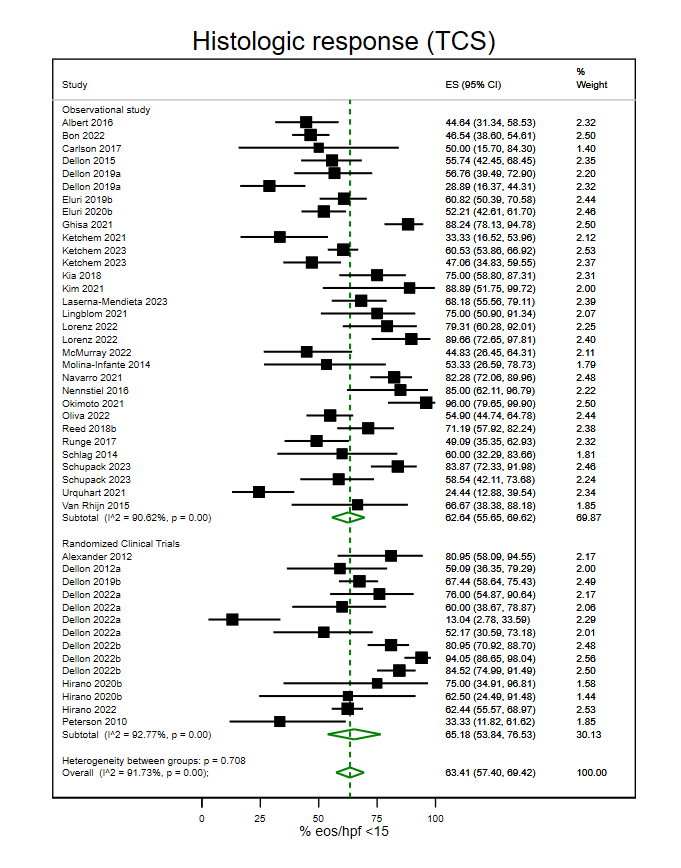


Figure E5. Forest plot of histologic response (≤5-7 eos/hpf) in patients treated withTCS


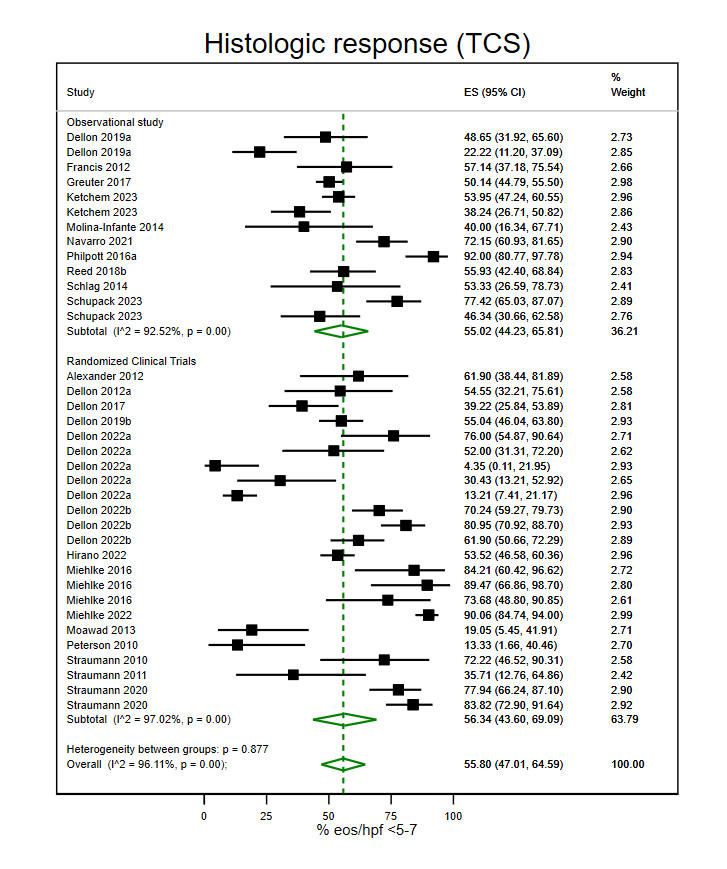


Figure E6. Forest plot of histologic response (≤1 eos/hpf) in patients treated withTCS


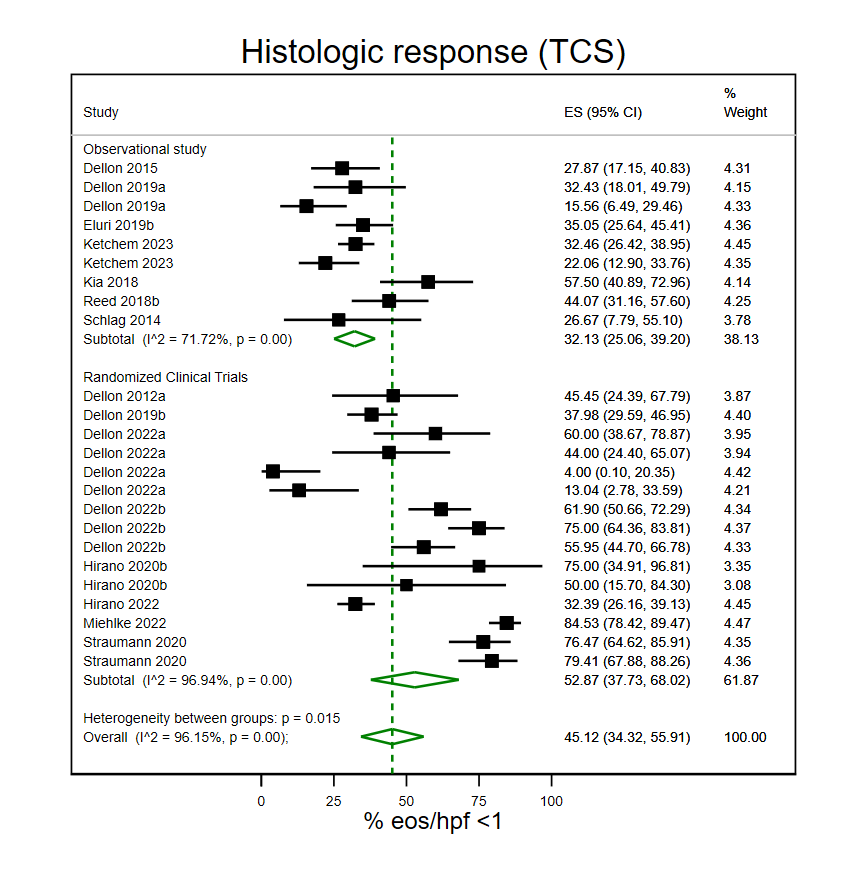


Figure E7. Forest plot of partial clinical response in patients treated with PPI


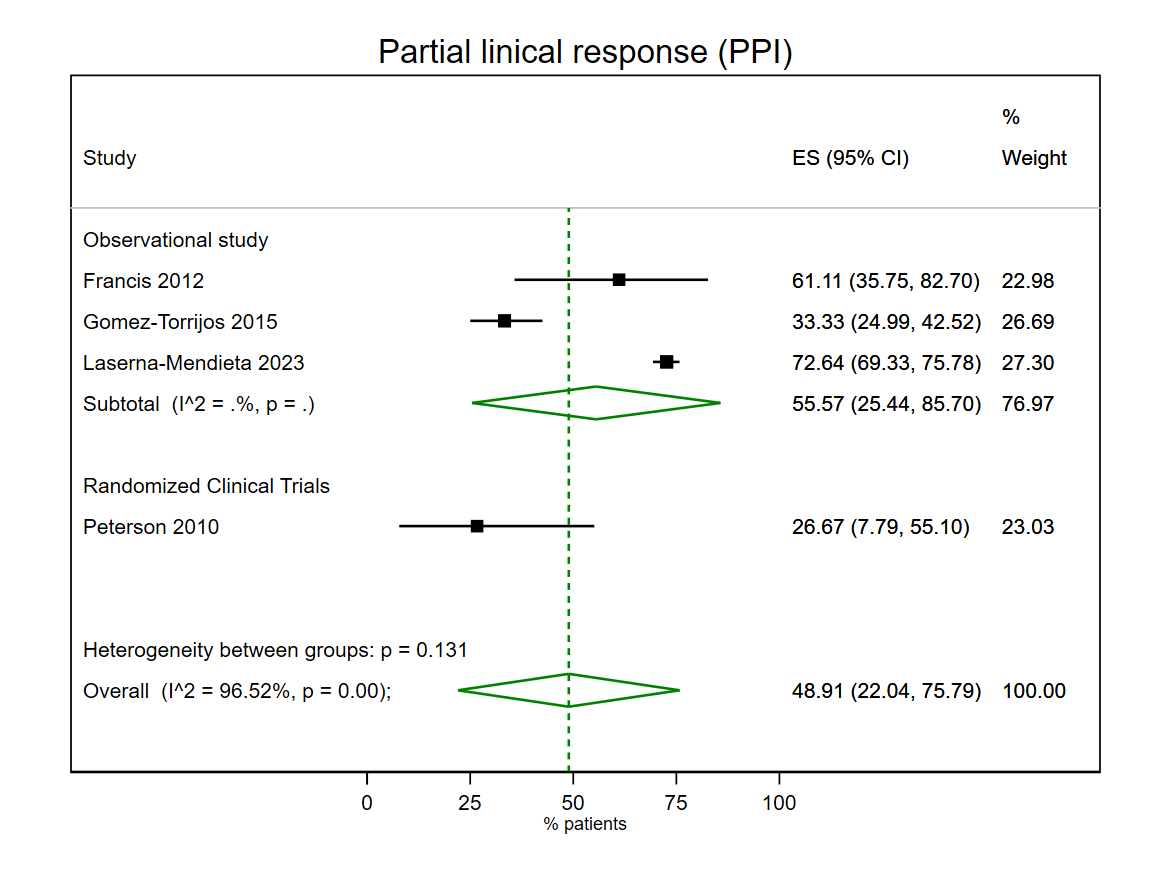


Figure E8. Forest plot of change in symptom score from baseline in patients treated with PPI


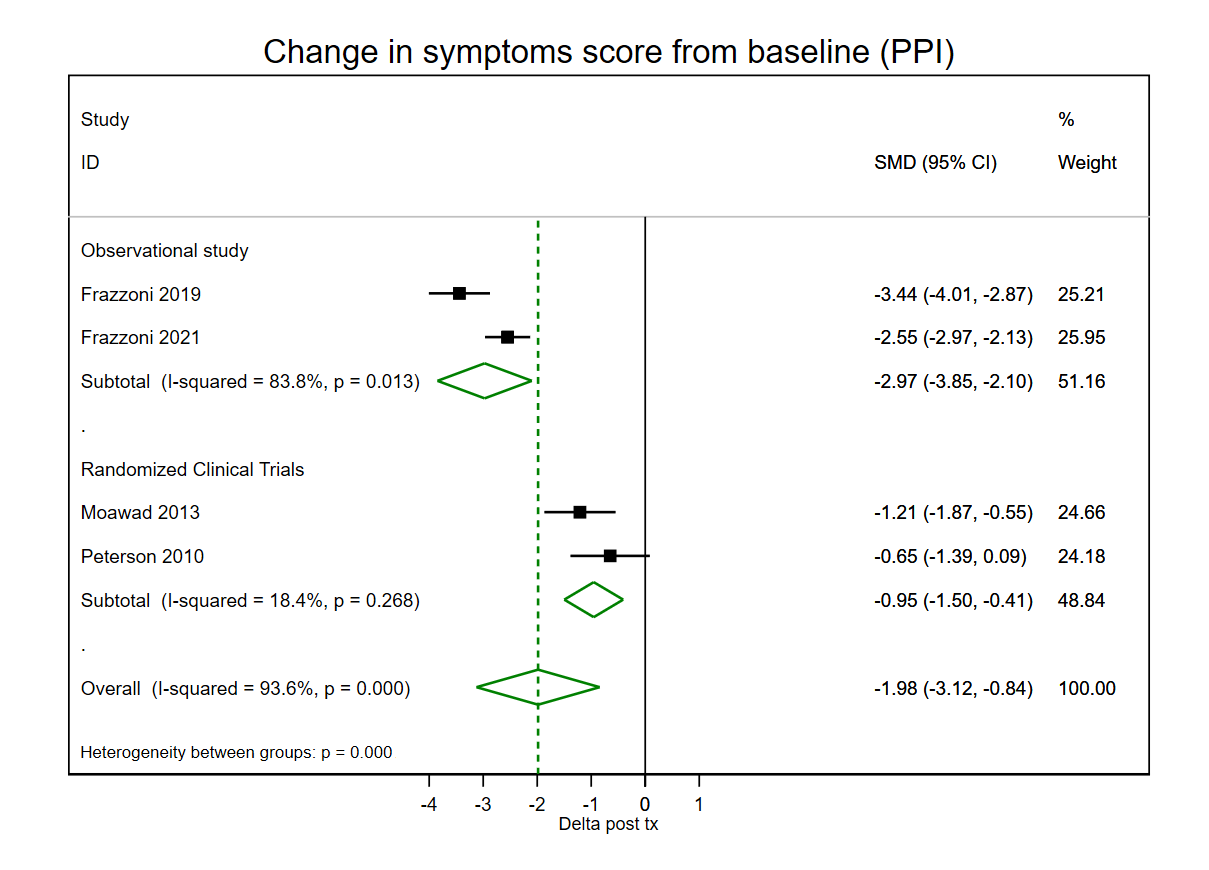


Figure E9. Forest plot of complete clinical response in patients treated with TCS


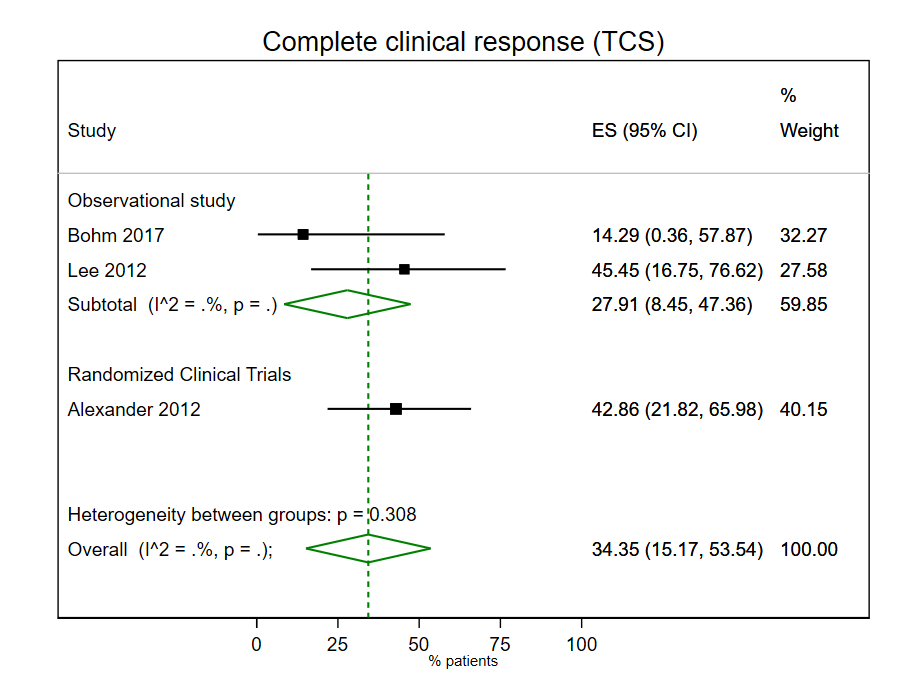


Figure E10. Forest plot of partial clinical response in patients treated with TCS


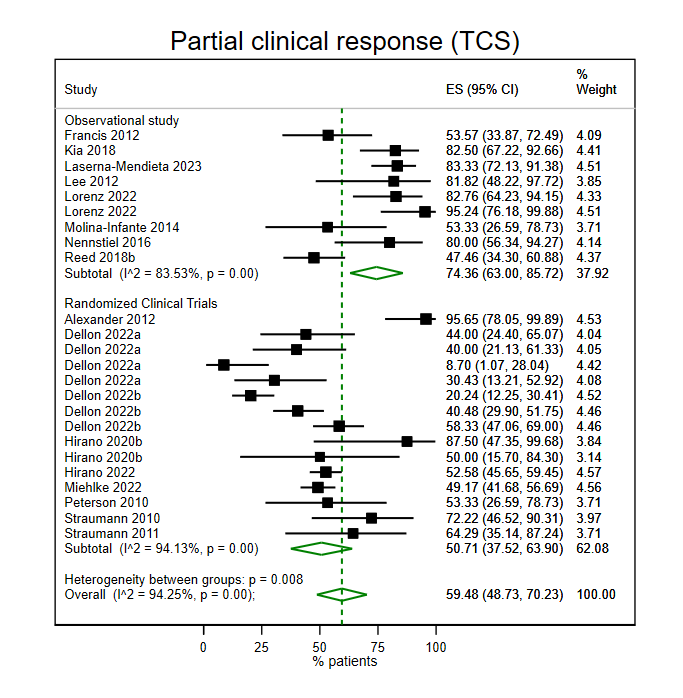


Figure E11. Forest plot of change in symptom score from baseline in patients treated with TCS


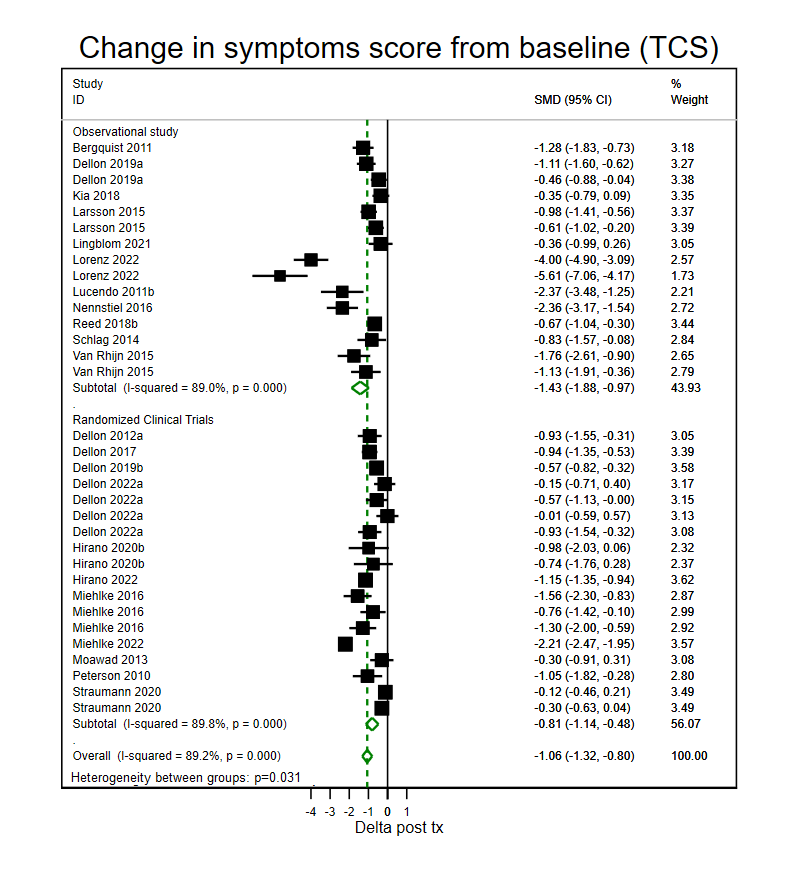


Figure E12. Forest plot of complete endoscopic response (% of patients with EREFS=0 post treatment) in patients treated with PPI


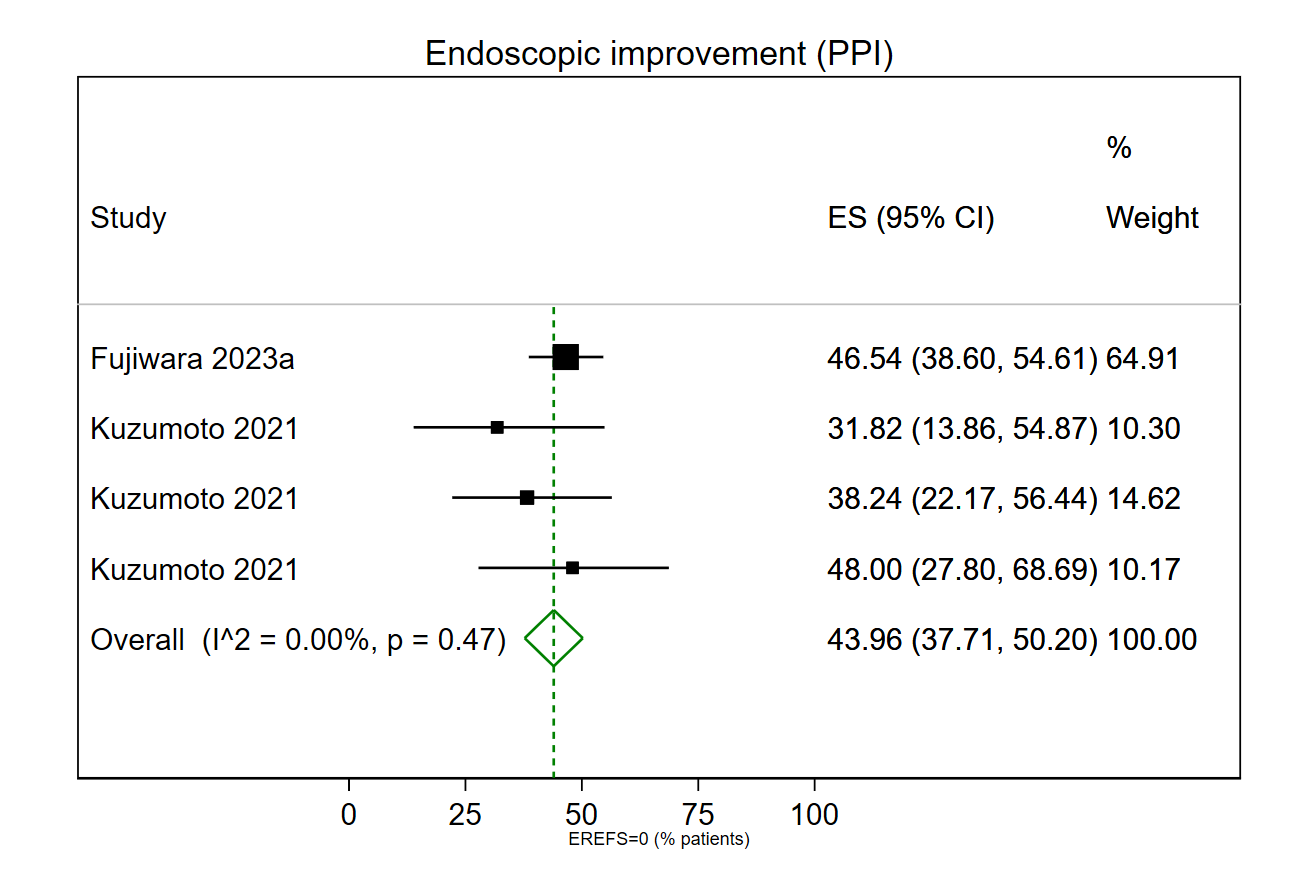


Figure E13. Forest plot of partial endoscopic response (% of patients with improvement or EREFS≤2 post treatment or documented global improvement in EREFS compared with baseline) in patients treated with PPI


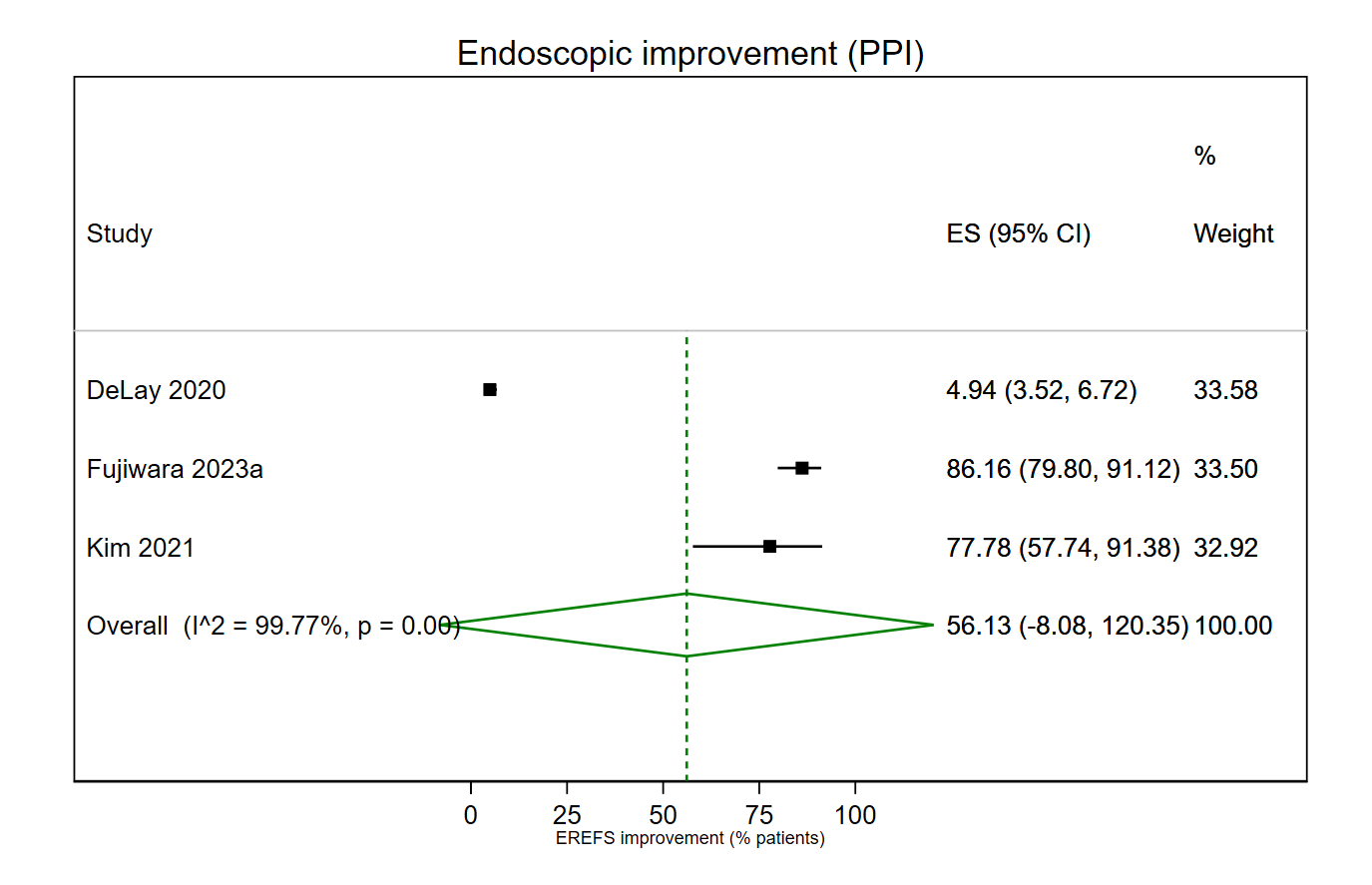


Figure E14. Forest plot of change in EREFS from baseline in patients treated with PPI


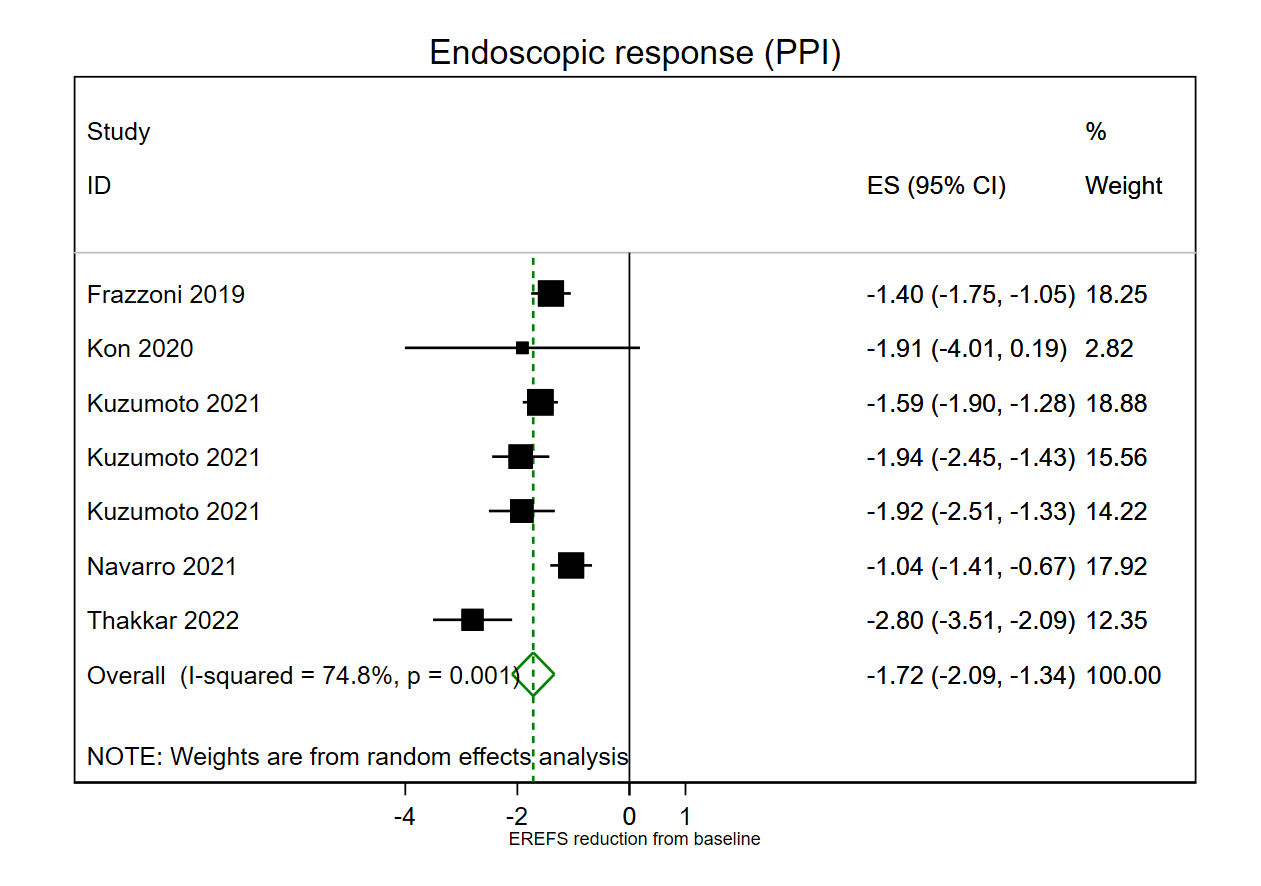


Figure E15. Forest plot of complete endoscopic response (% of patients with EREFS=0 post treatment) in patients treated with TCS


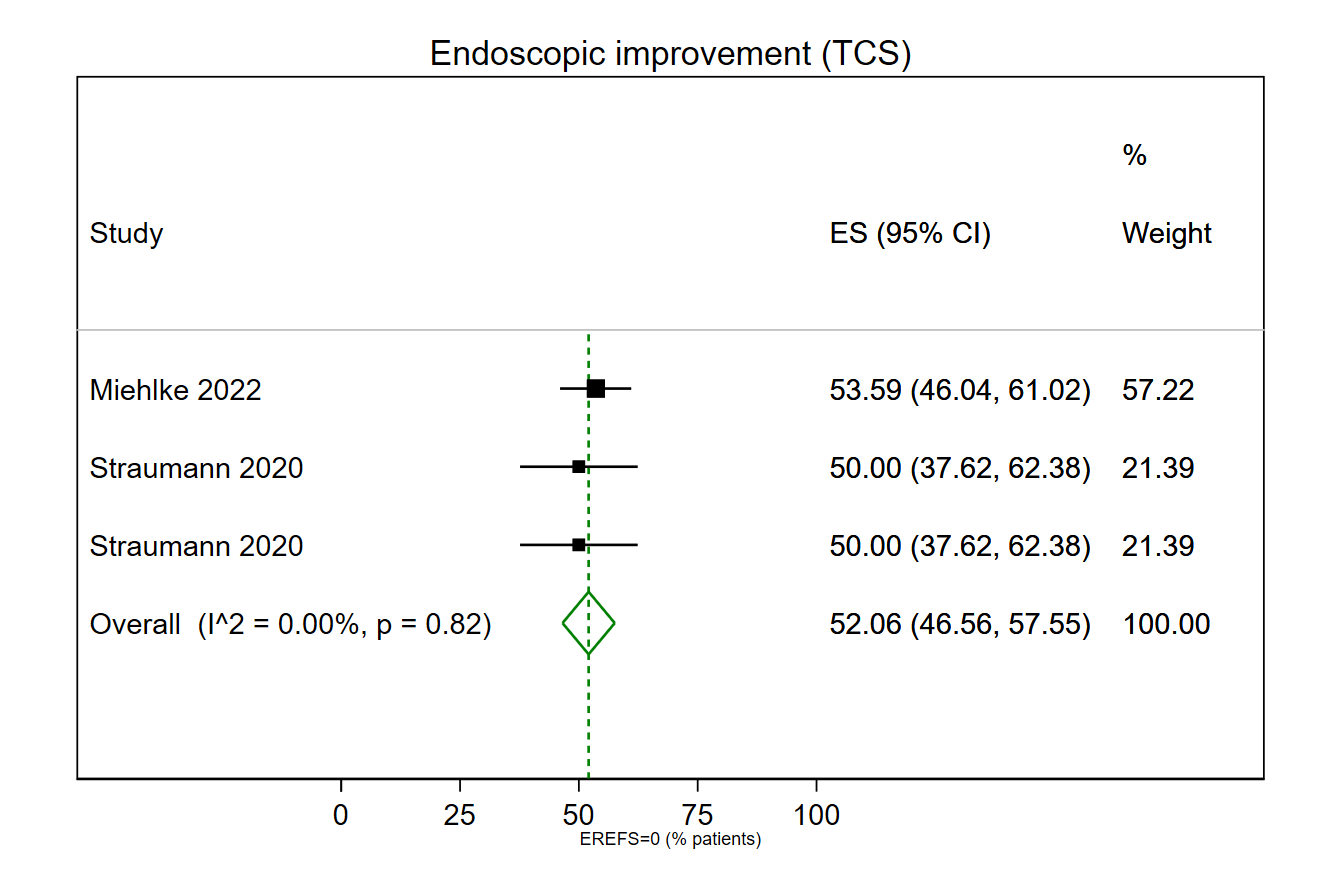


Figure E16. Forest plot of partial endoscopic response (% of patients with improvement or EREFS≤2 post treatment or documented global improvement in EREFS compared with baseline) in patients treated with TCS


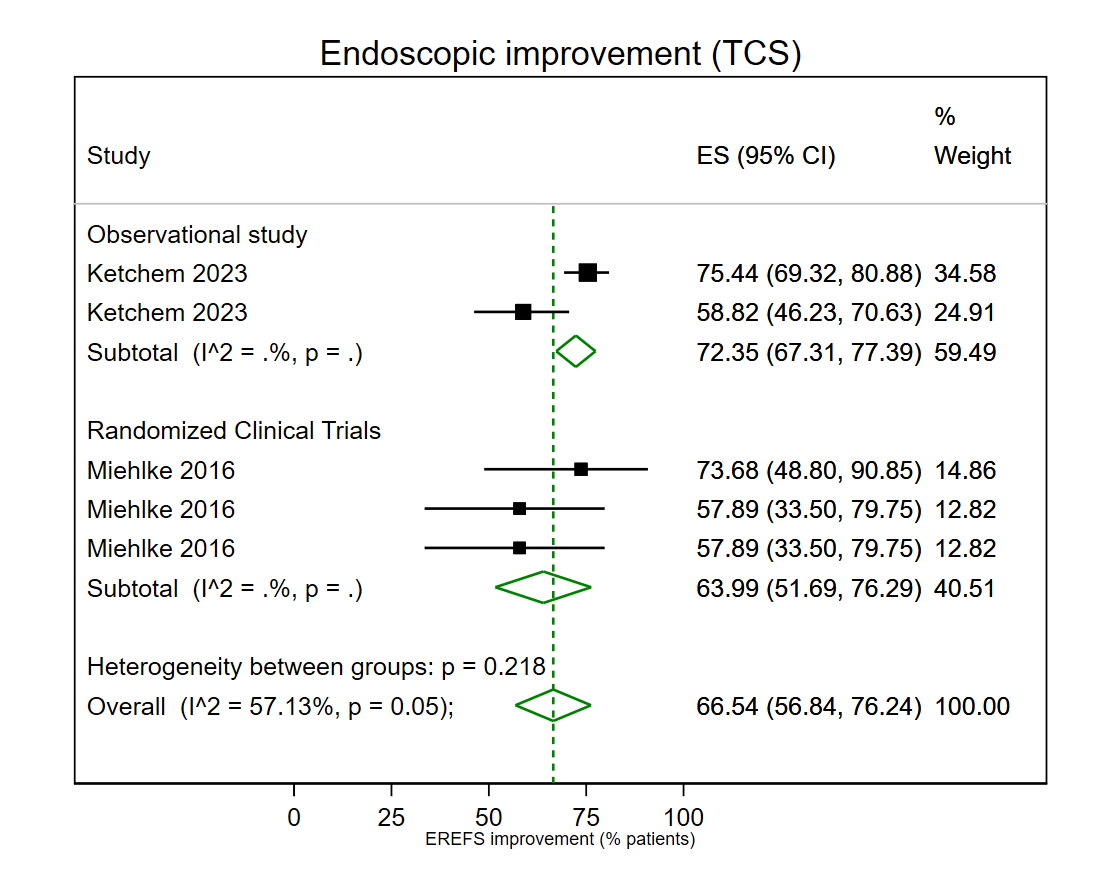


Figure E17. Forest plot of change in EREFS from baseline in patients treated with TCS


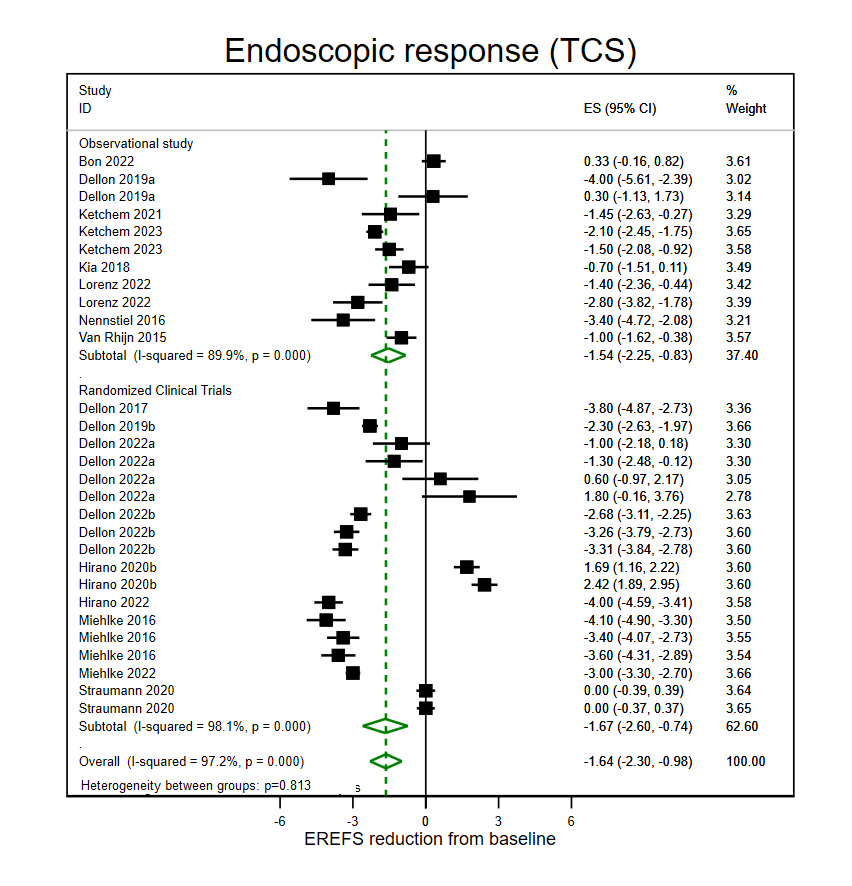


Figure E18. Forest plot of change in EREFS (Edema domain) from baseline in patients treated with TCS


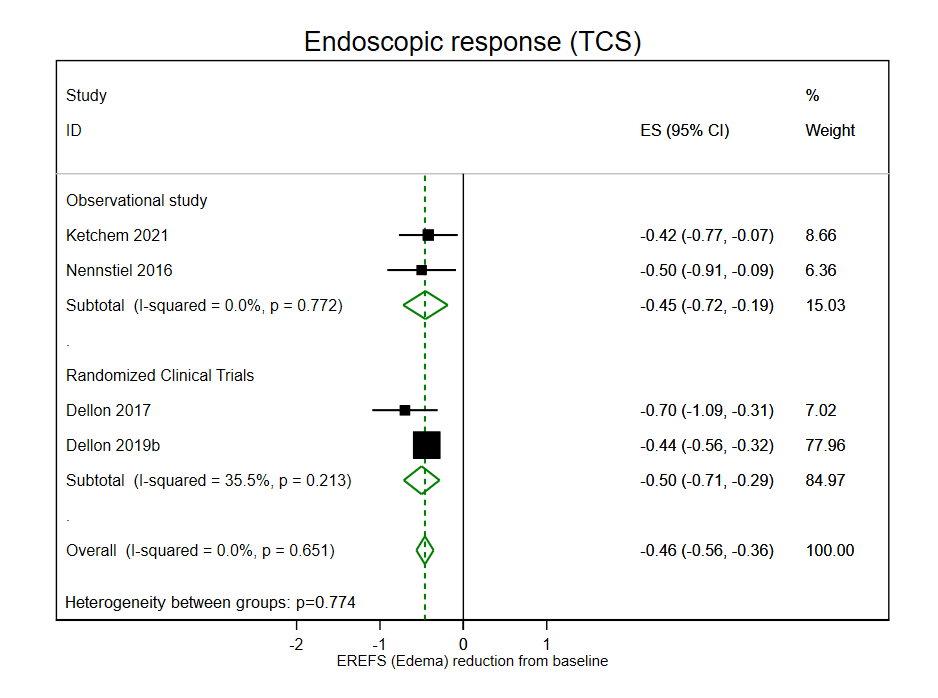


Figure E19. Forest plot of change in EREFS (Rings domain) from baseline in patients treated with TCS


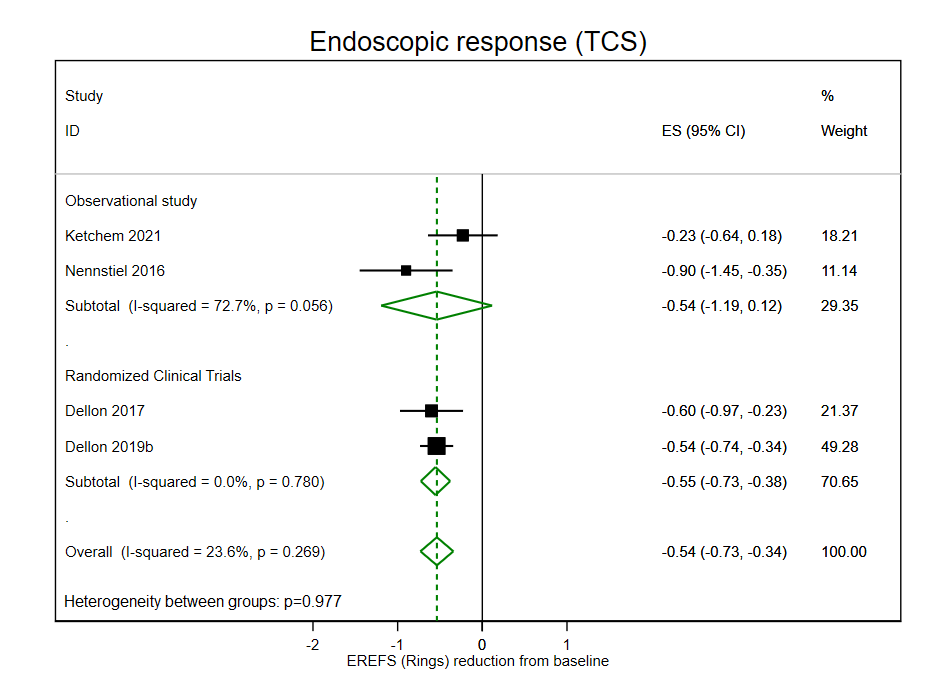


Figure E20. Forest plot of change in EREFS (Exudates domain) from baseline in patients treated with TCS


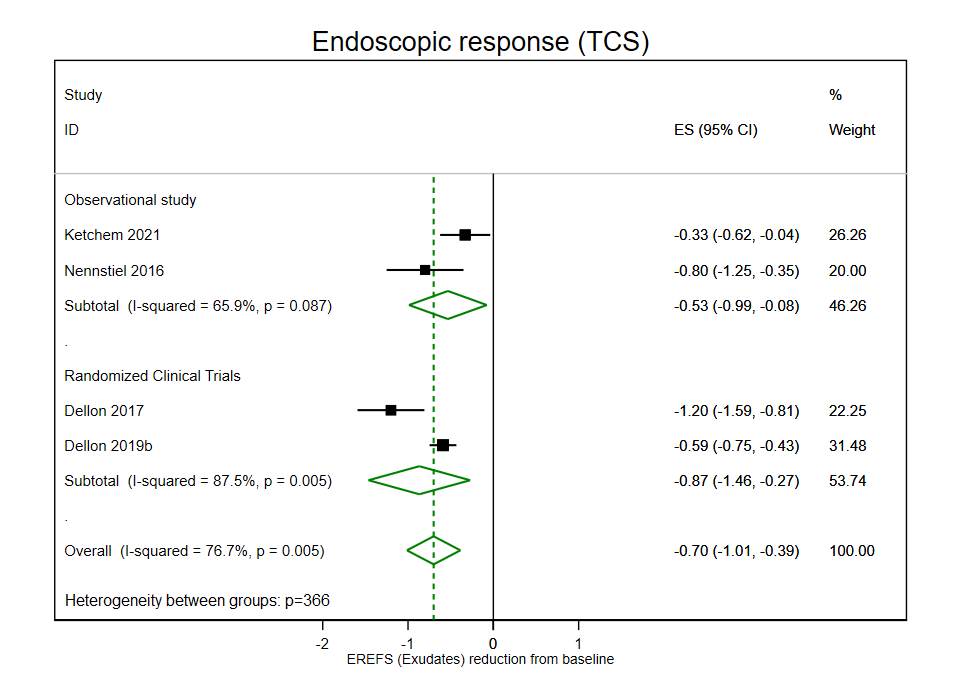


Figure E21. Forest plot of change in EREFS (Furrows domain) from baseline in patients treated with TCS


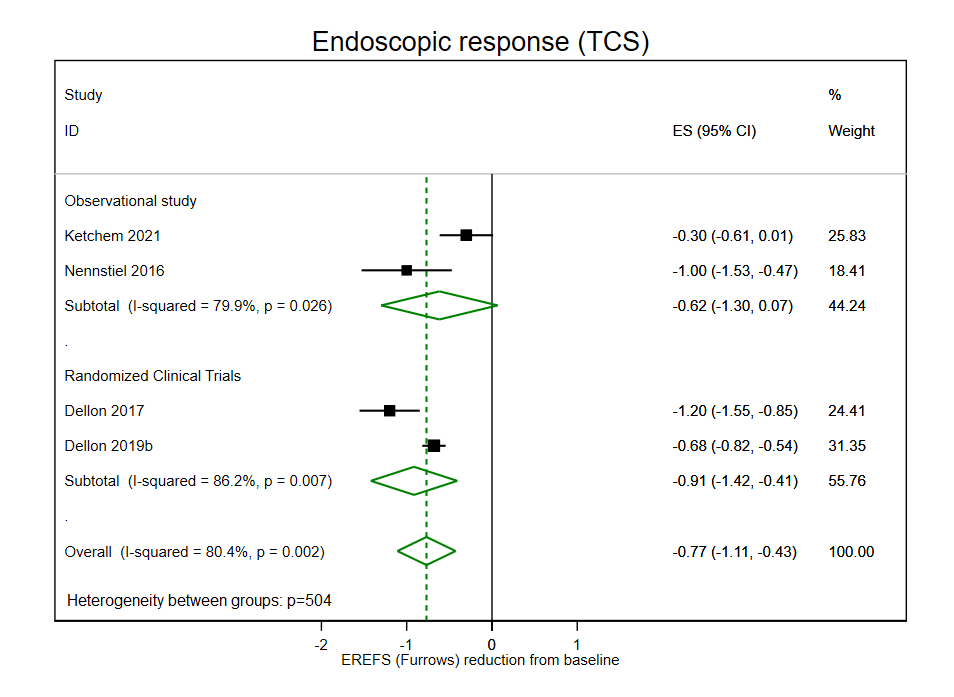


Figure E22. Forest plot of change in EREFS (strictures domain) from baseline in patients treated with TCS


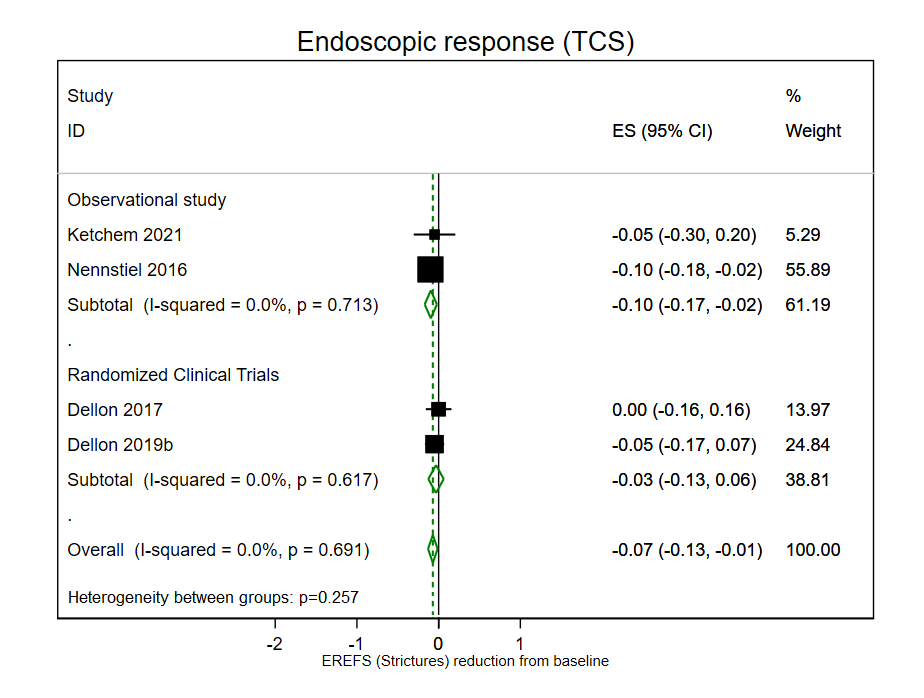


Figure E23. Forest plot of annual relapse rate (events per person-year) in patients treated with TCS


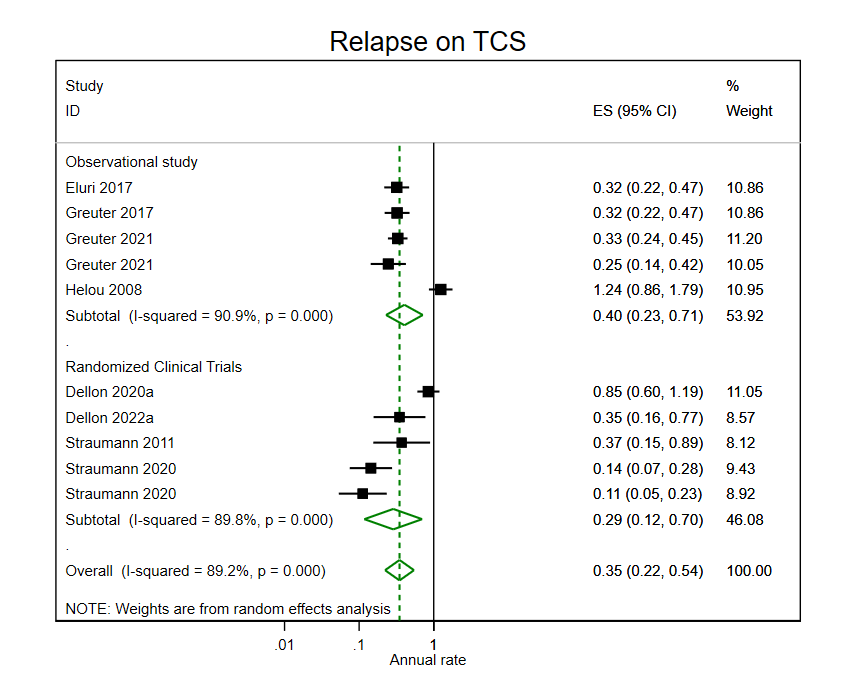


Figure E24. Forest plot of annual rate of treatment emerging adverse events (events per person-year) in patients treated with TCS


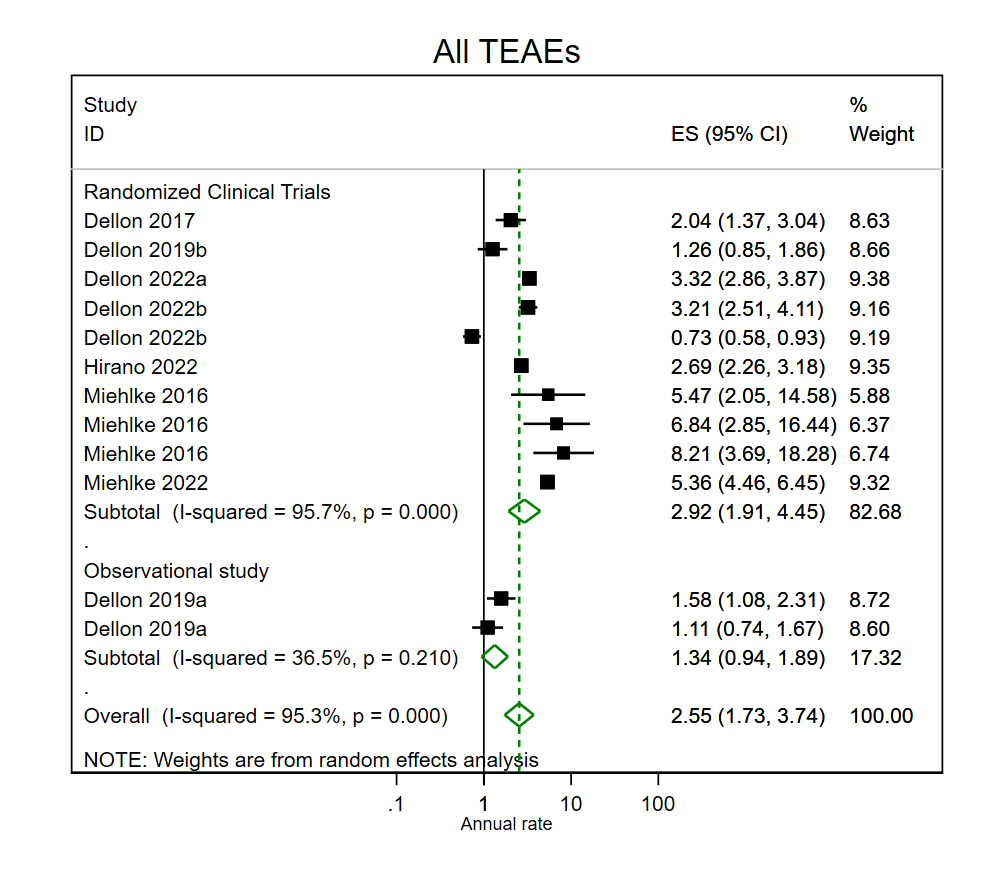


Figure E25. Forest plot of annual rate of serious treatment emerging adverse events (events per person-year) in patients treated with TCS


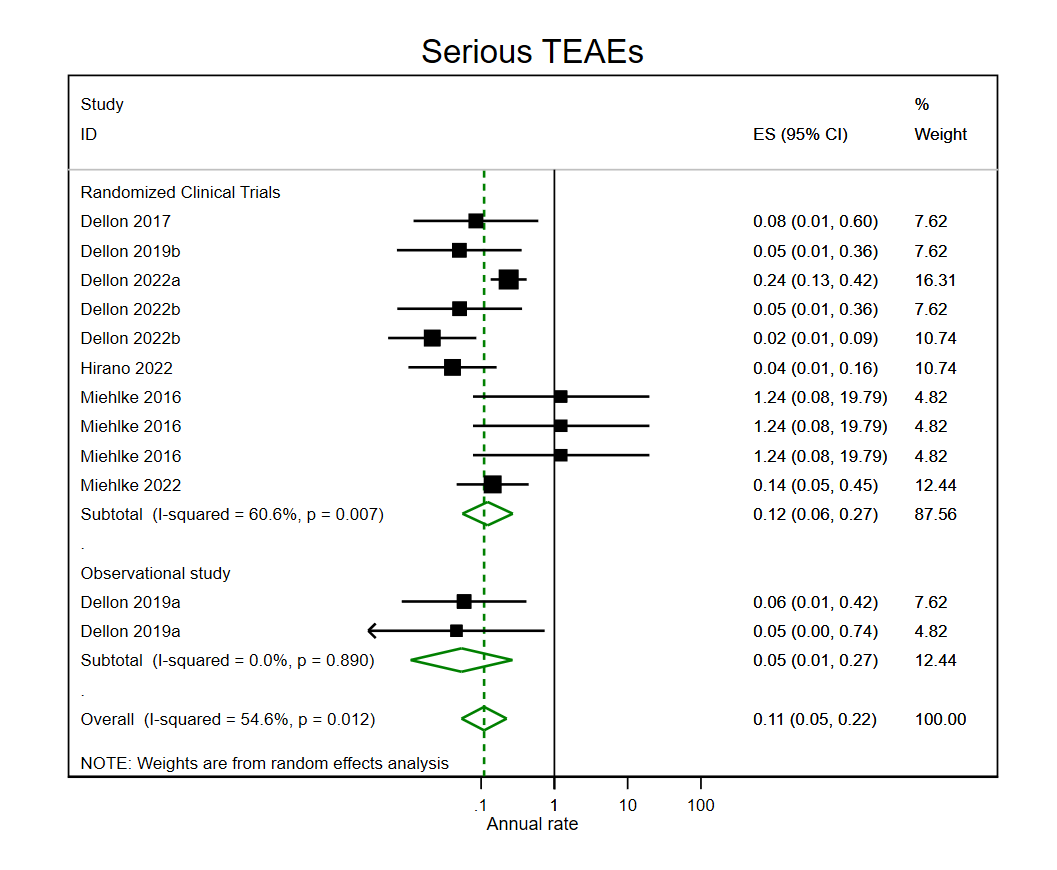


Figure E26. Forest plot of annual rate of non-serious treatment emerging adverse events (events per person-year) in patients treated with TCS


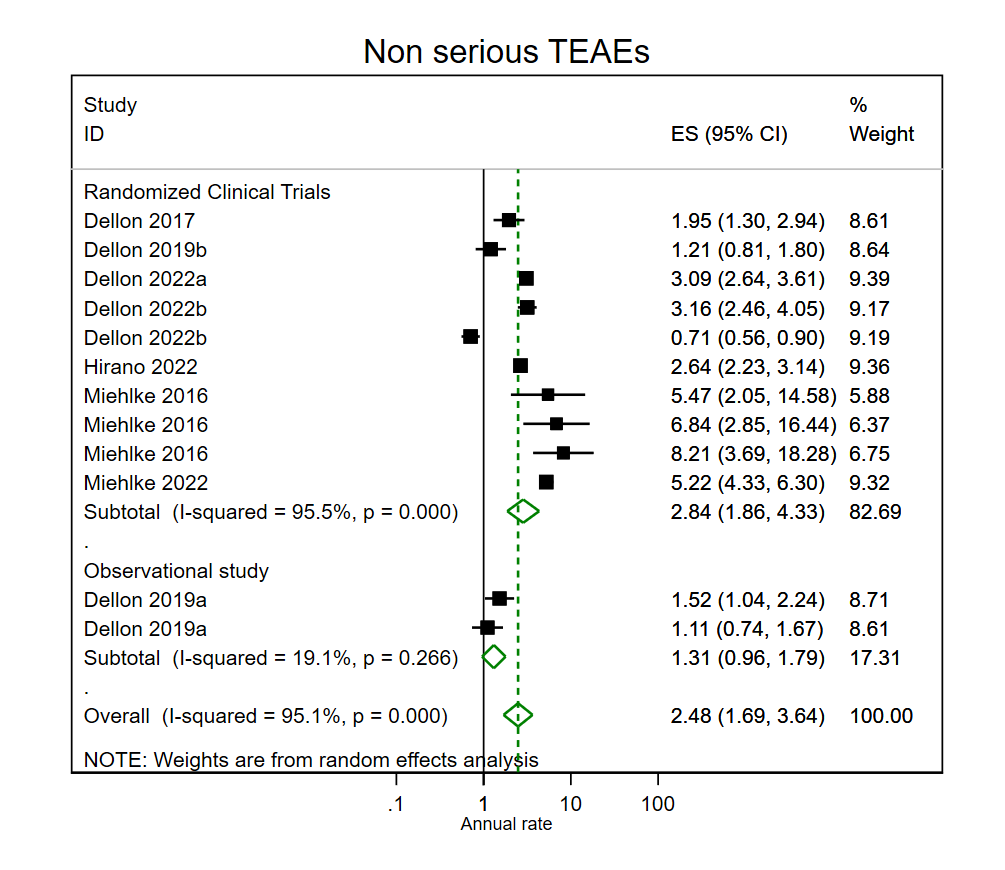


Figure E27. Forest plot of annual rate of treatment emerging adverse events leading to hospitalization (events per person-year) in patients treated with TCS


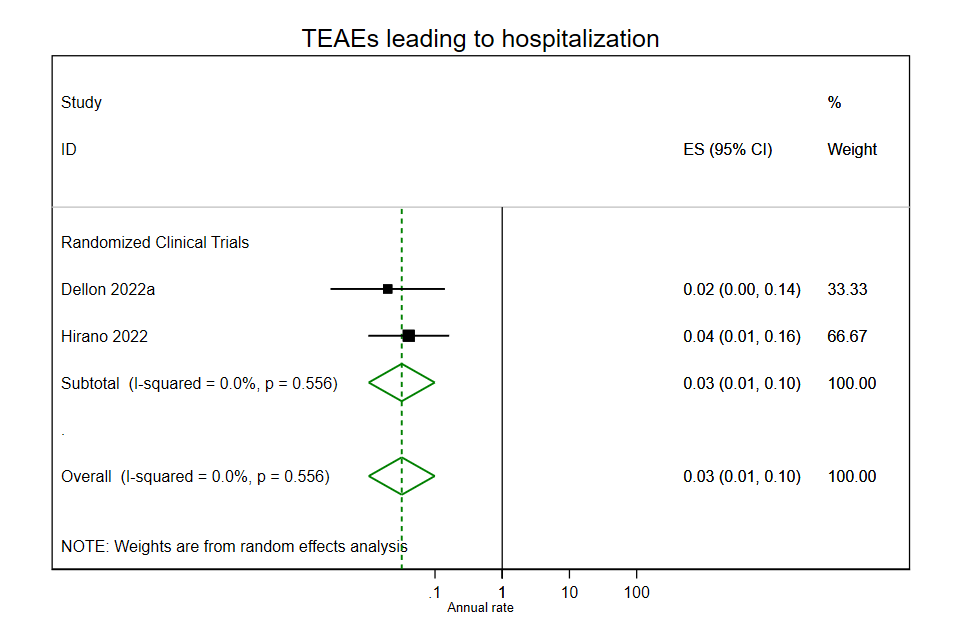


Figure E28. Forest plot of annual rate of treatment emerging adverse events leading to discontinuation (events per person-year) in patients treated with TCS


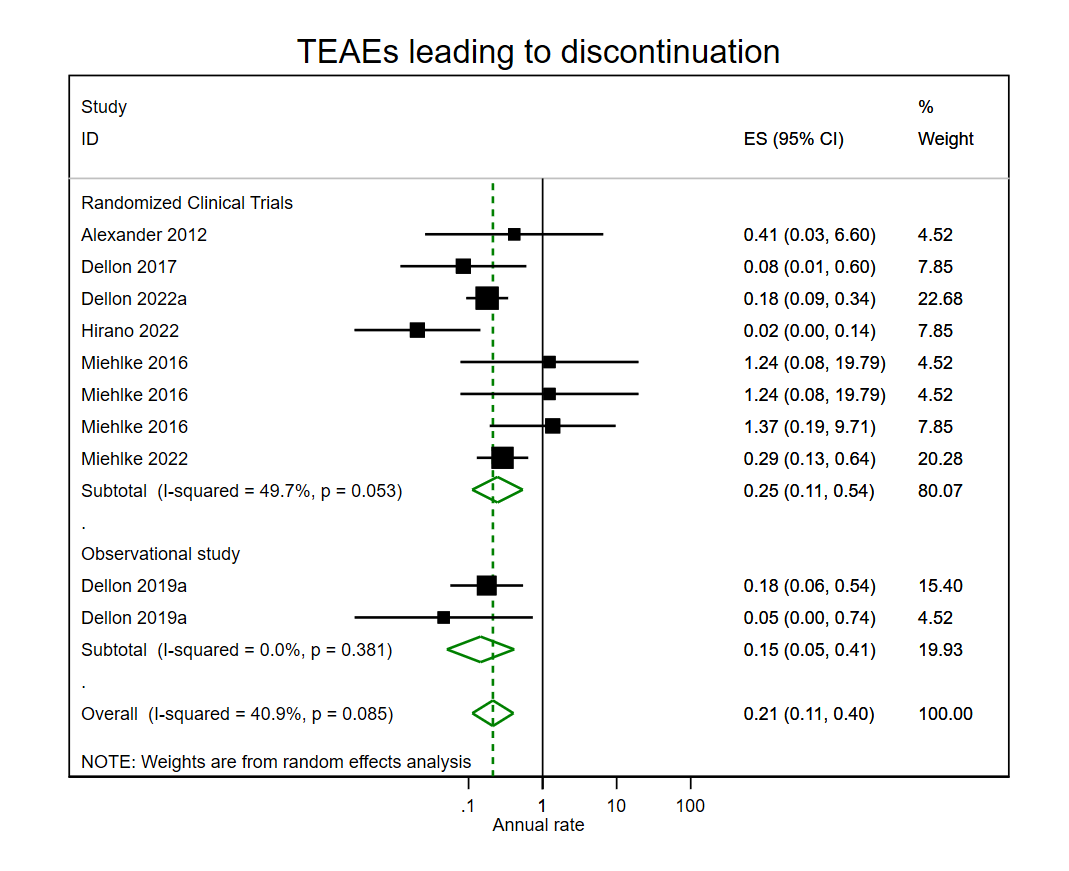


Figure E29. Forest plot of annual dilation rate (events per person-year) in patients treated with TCS (any time)


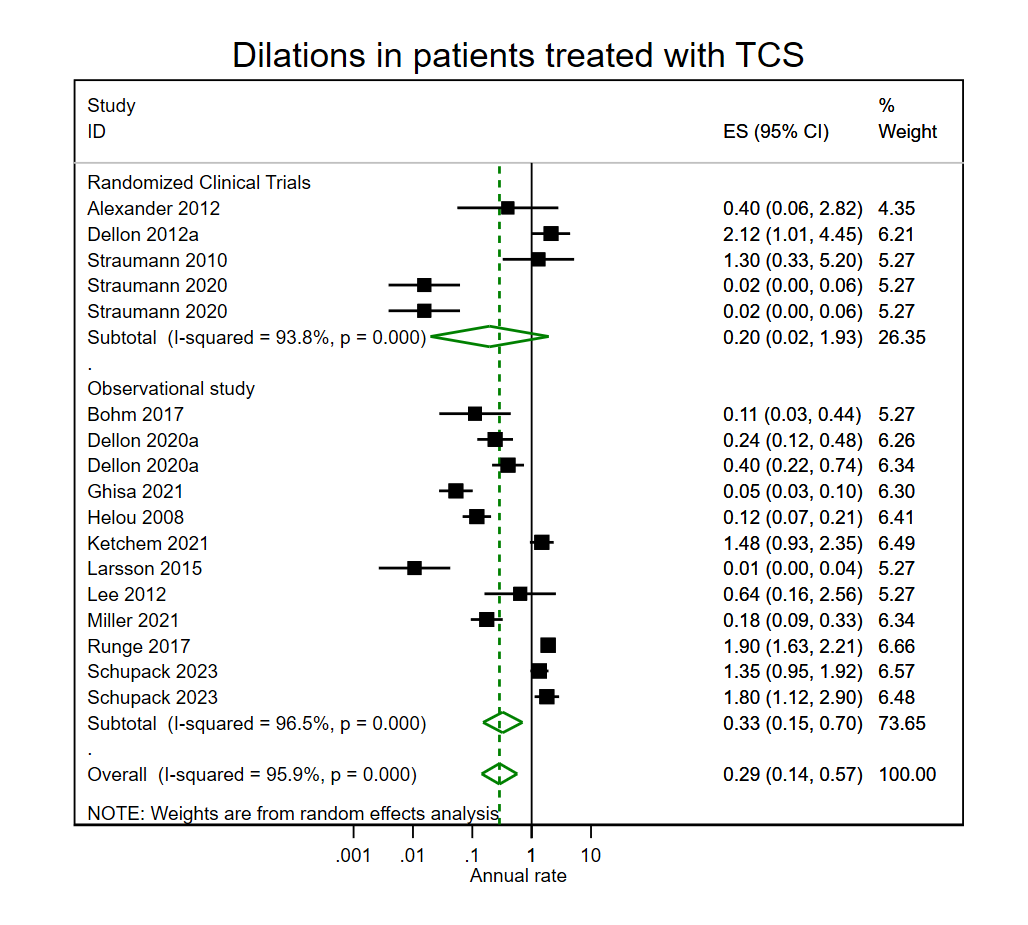


Figure E30. Forest plot of annual dilation rate (events per person-year) in patients treated with TCS (during treatment)


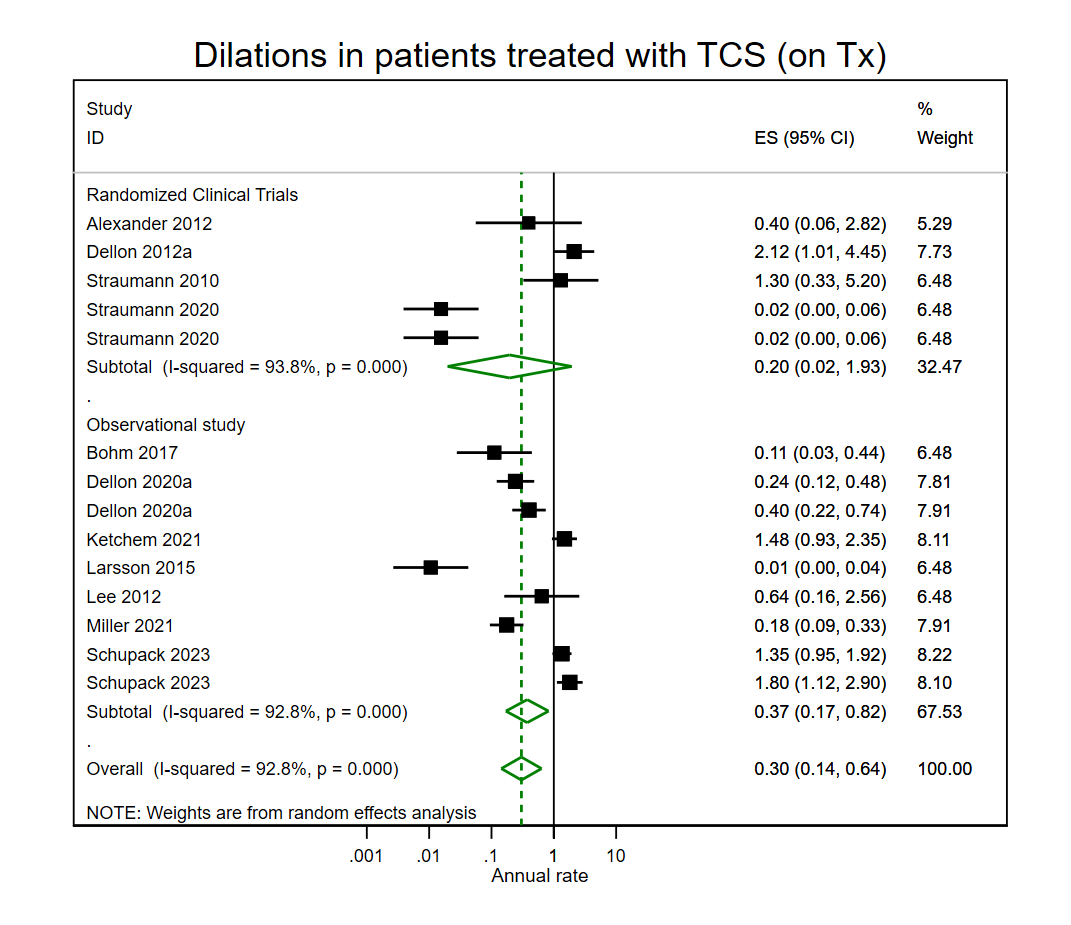


Figure E31. Forest plot of annual rate of infections and infestations (events per person-year) in patients treated with TCS


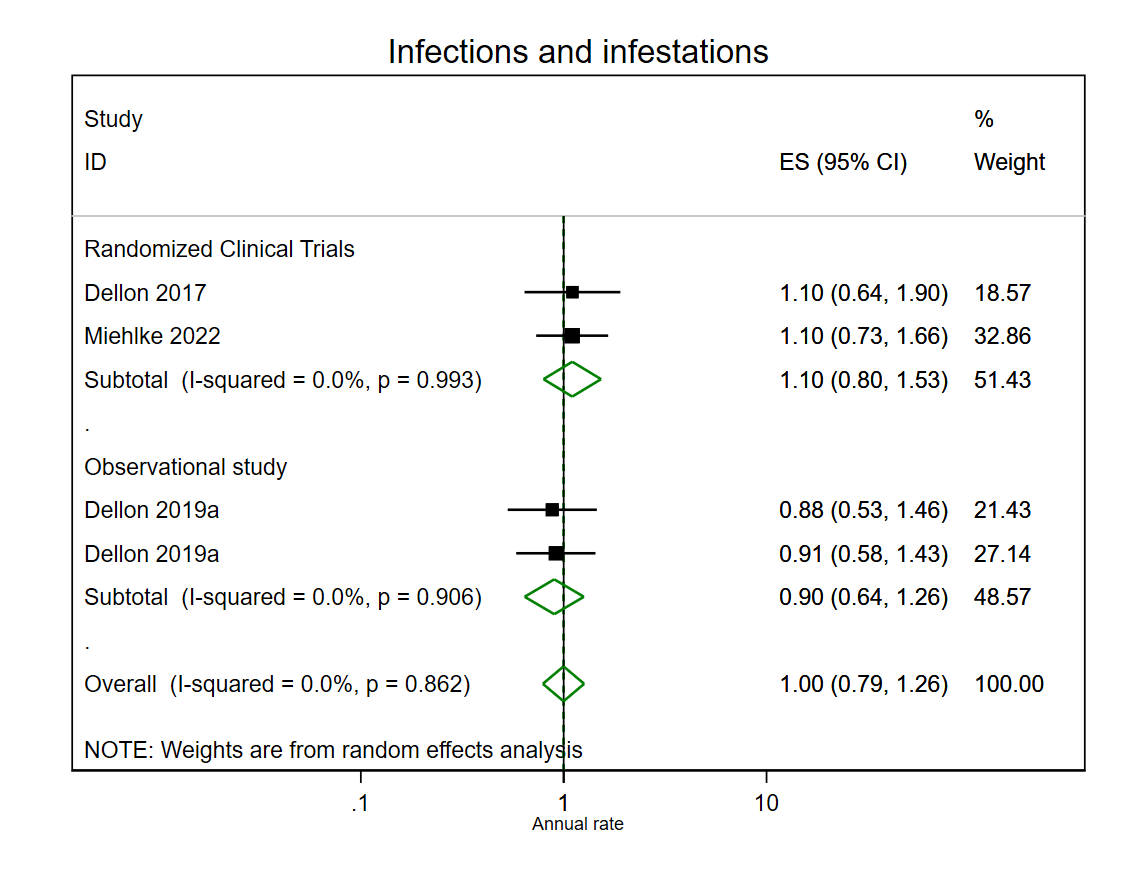


Figure E32. Forest plot of annual rate of oral candidiasis (events per person-year) in patients treated with TCS


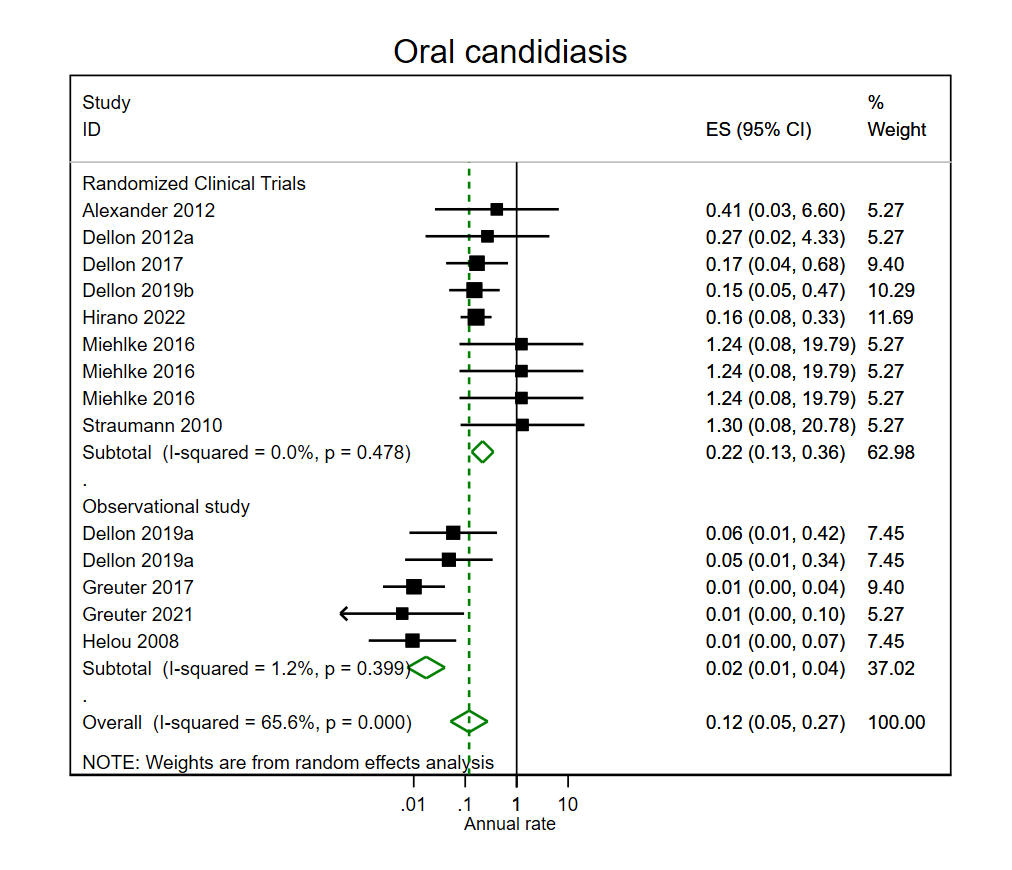


Figure E33. Forest plot of annual rate of esophageal (events per person-year) in patients treated with TCS


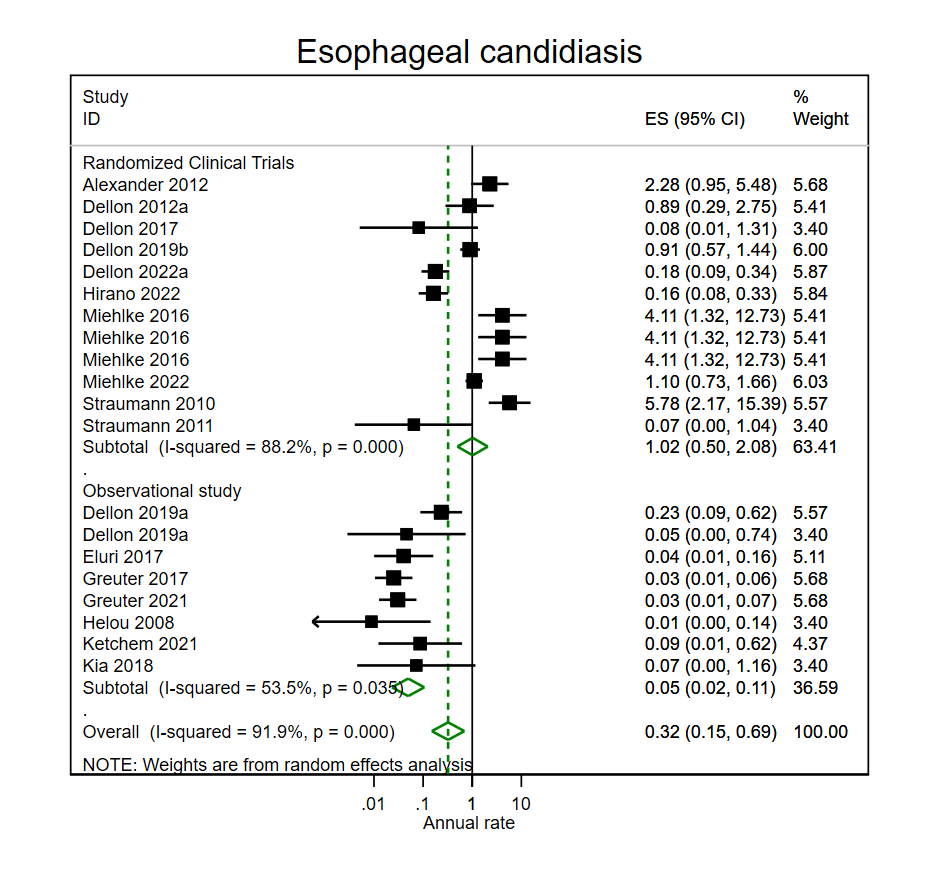


Figure E34. Forest plot of annual rate of upper tract infections (events per person-year) in patients treated with TCS


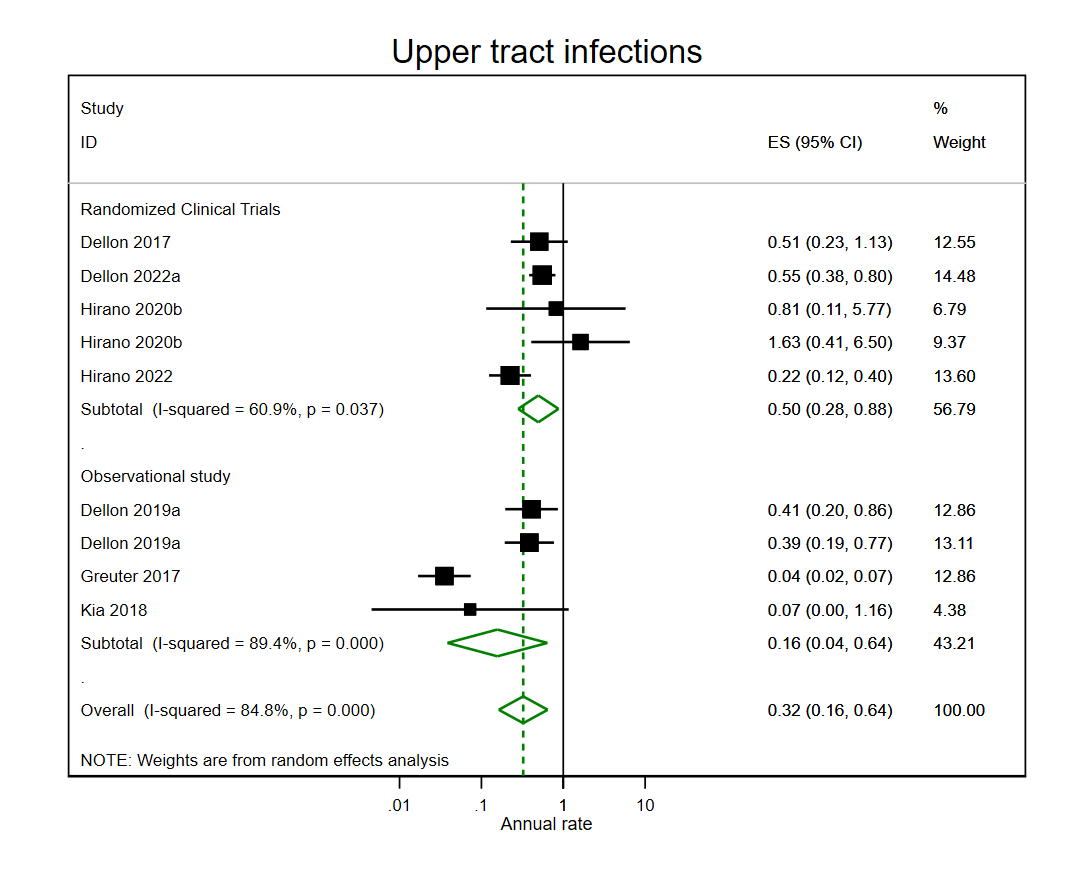


Figure E35. Forest plot of annual rate of sinusitis (events per person-year) in patients treated with TCS


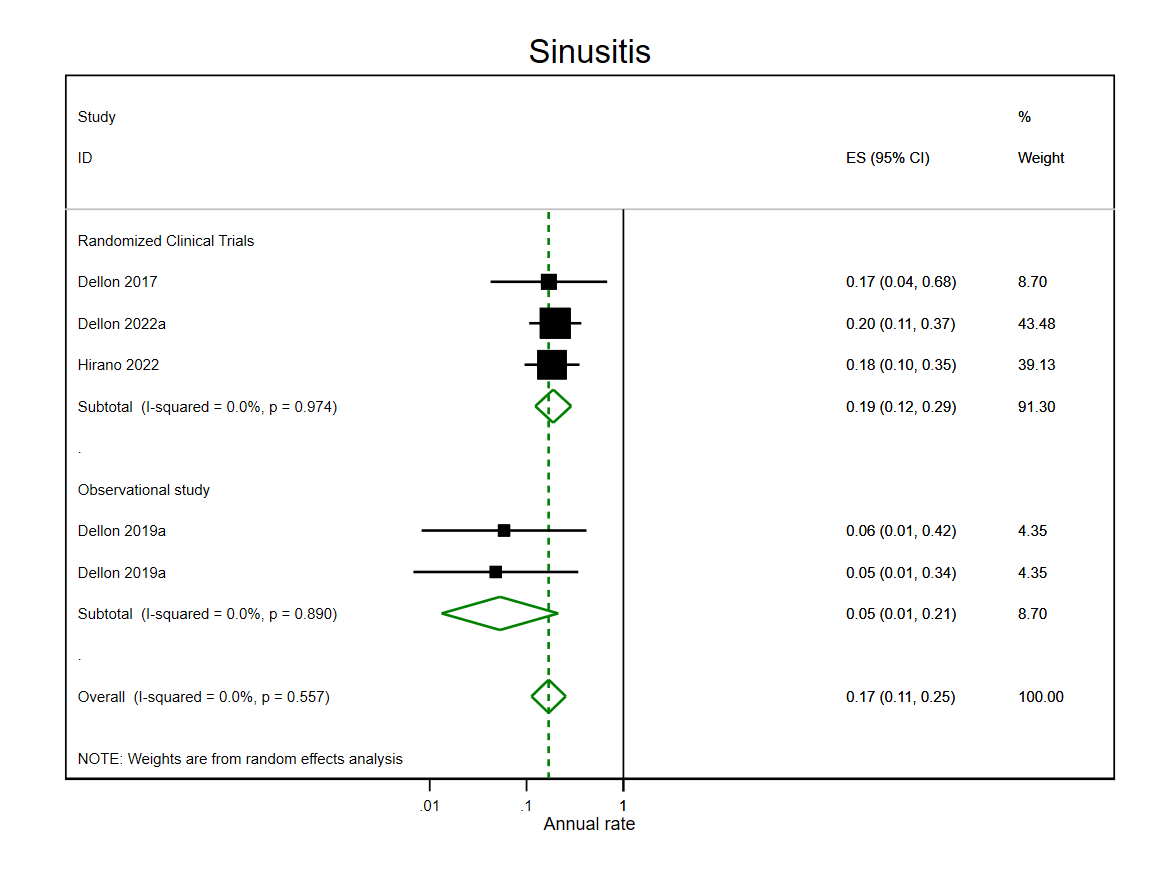


Figure E36. Forest plot of annual rate of gastro-intestinal disorders (events per person-year) in patients treated with TCS


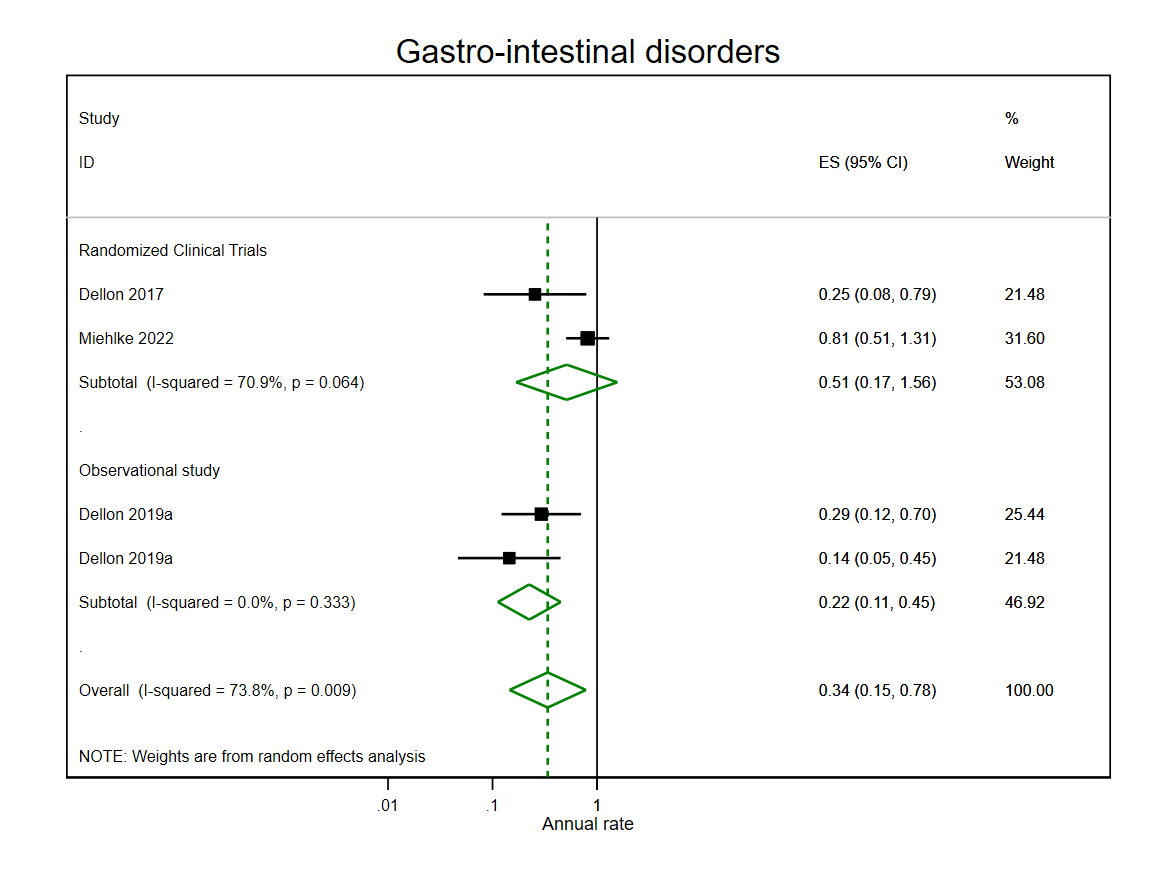


Figure E37. Forest plot of annual rate of diarrhea (events per person-year) in patients treated with TCS


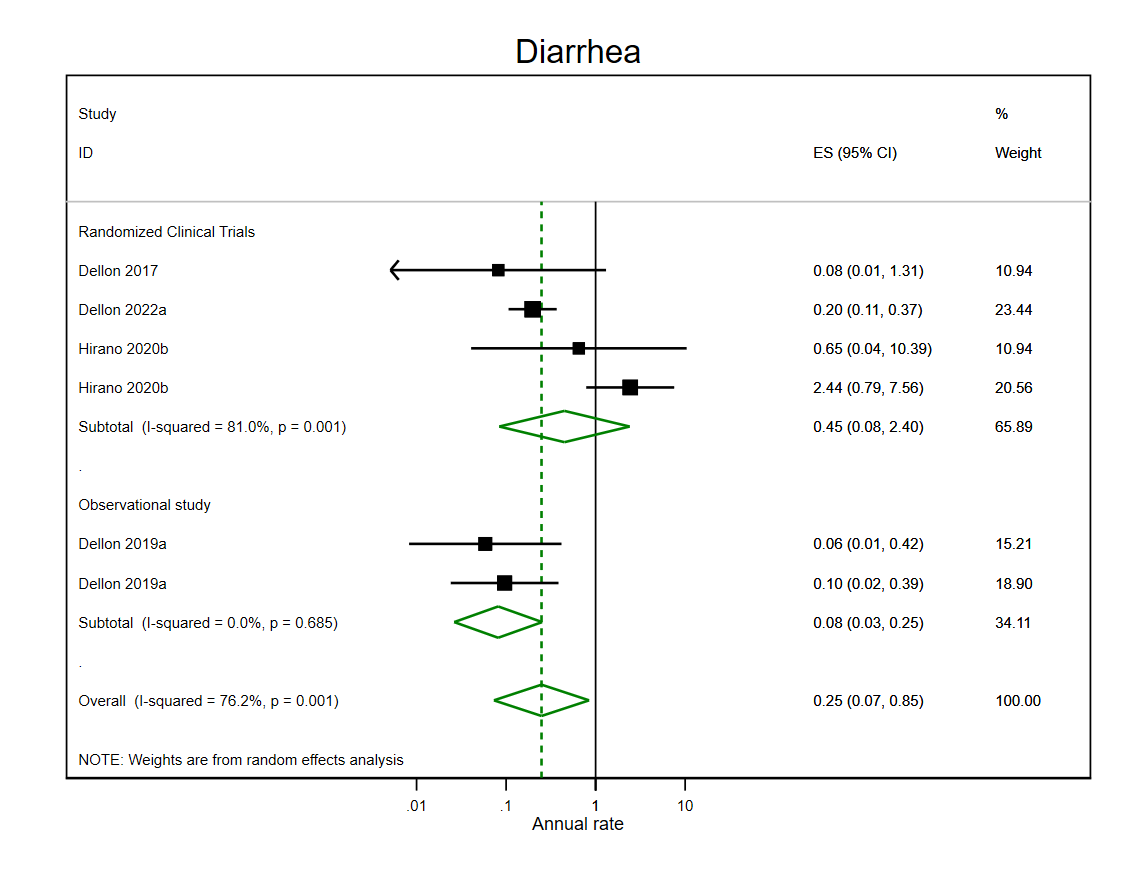


Figure E38. Forest plot of annual rate of nausea or vomiting (events per person-year) in patients treated with TCS


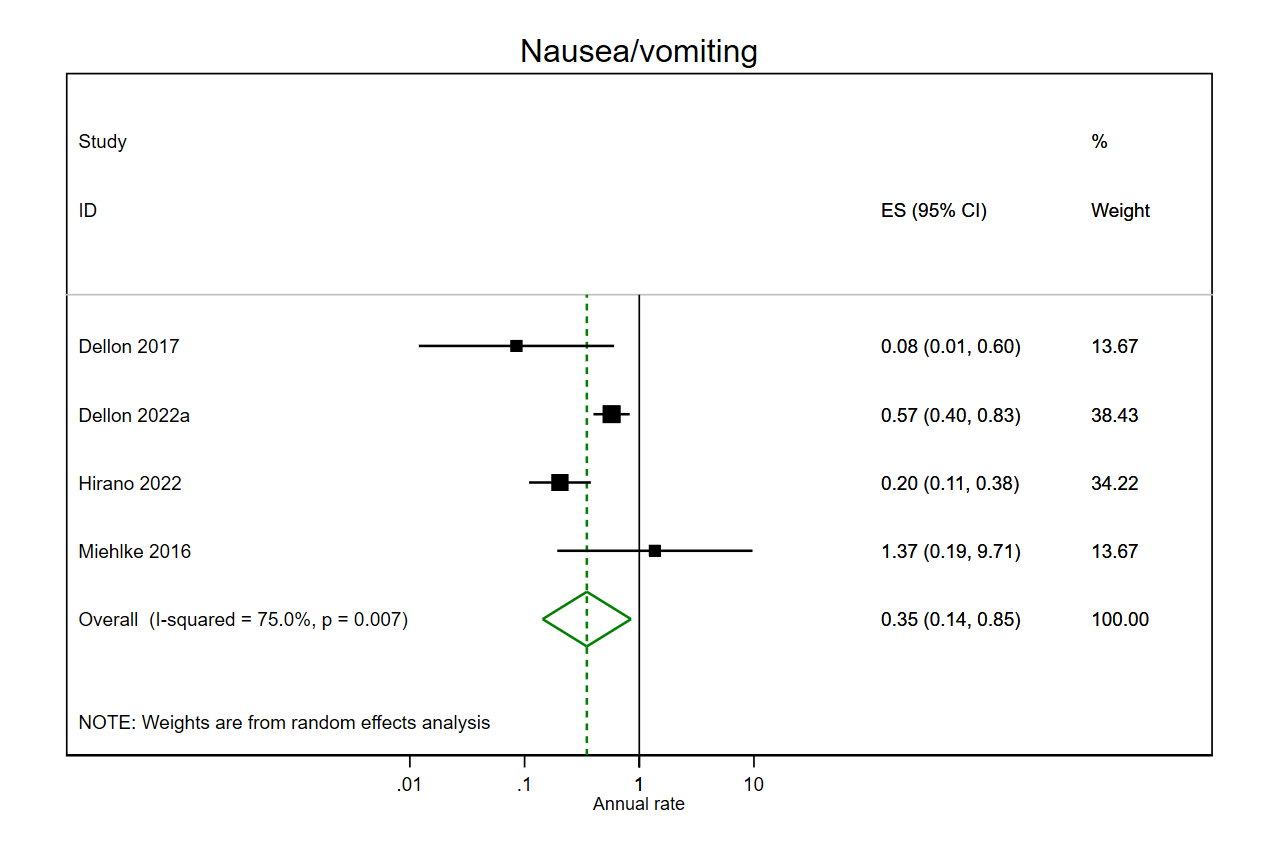


Figure E39. Forest plot of annual rate of abdominal pain/discomfort (events per person-year) in patients treated with TCS


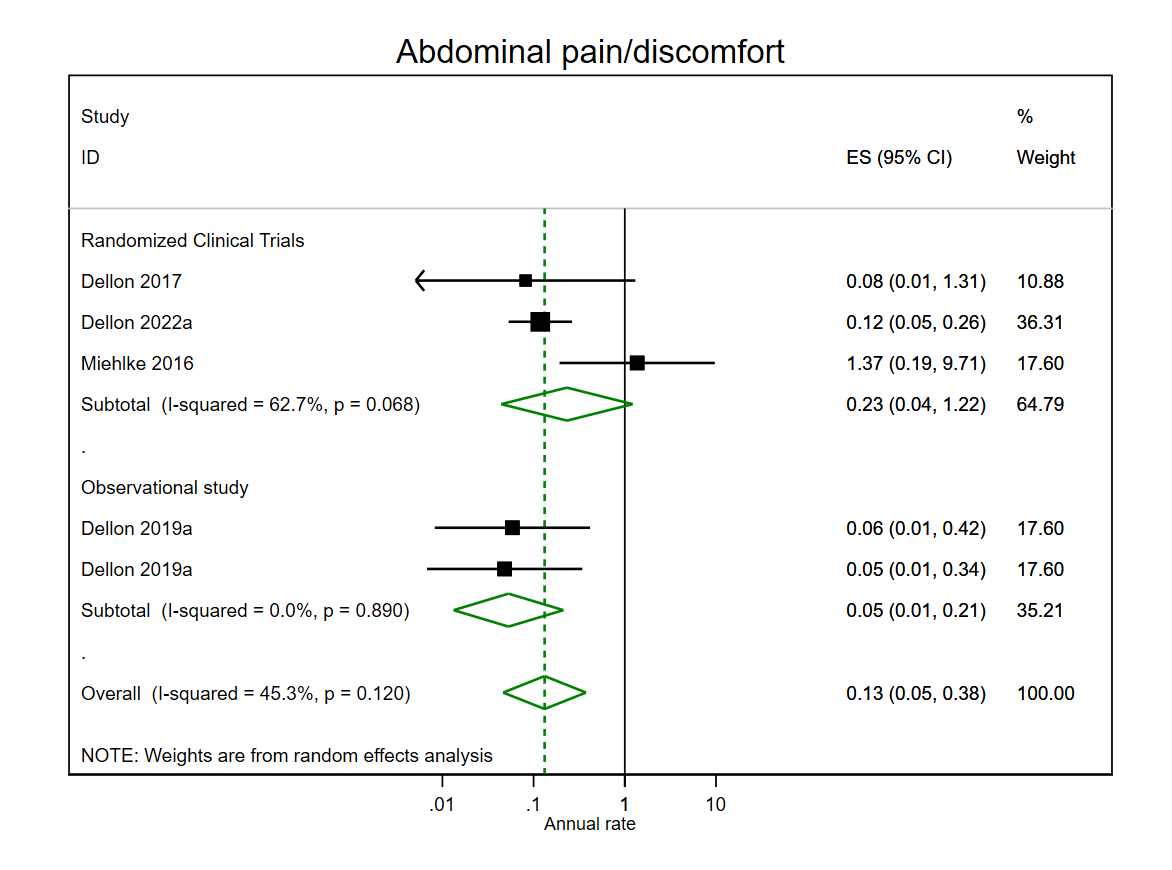


Figure E40. Forest plot of annual rate of respiratory disorders (events per person-year) in patients treated with TCS


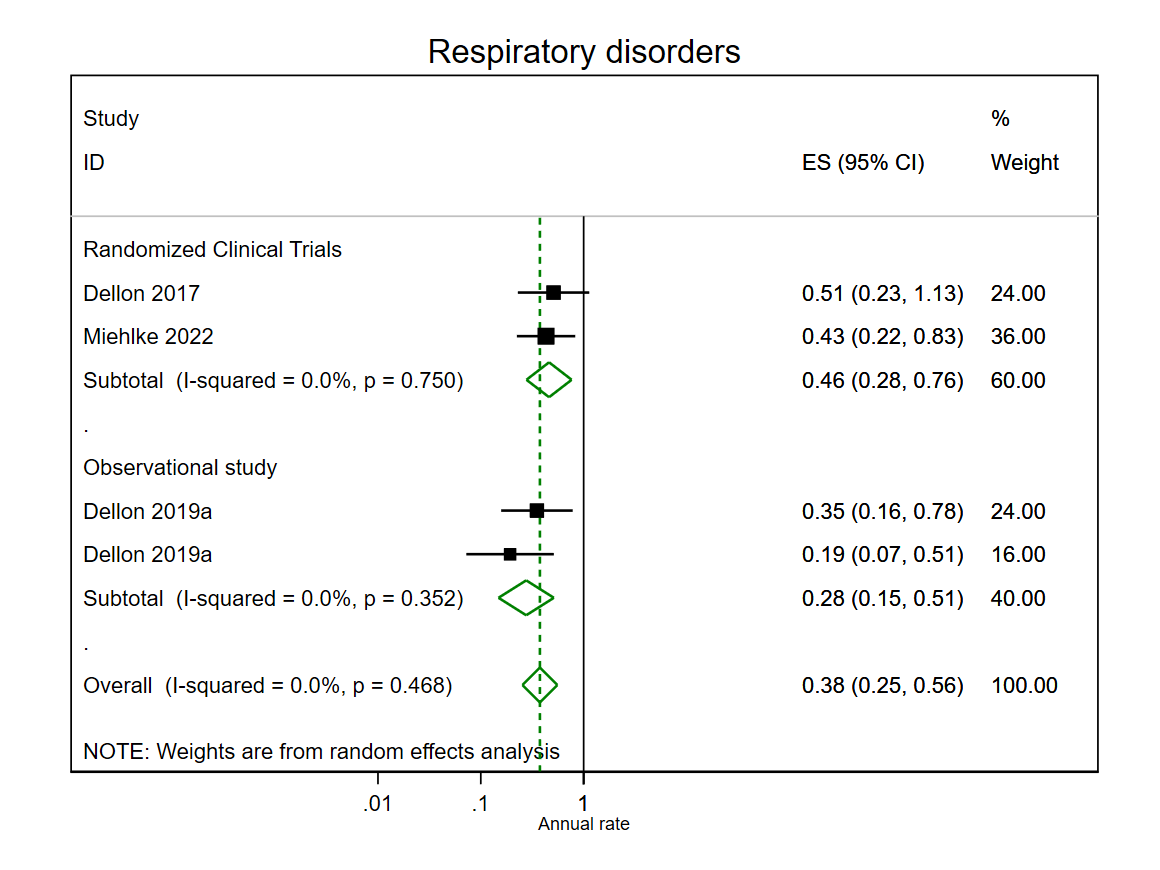


Figure E41. Forest plot of annual rate of oropharyngeal pain (events per person-year) in patients treated with TCS


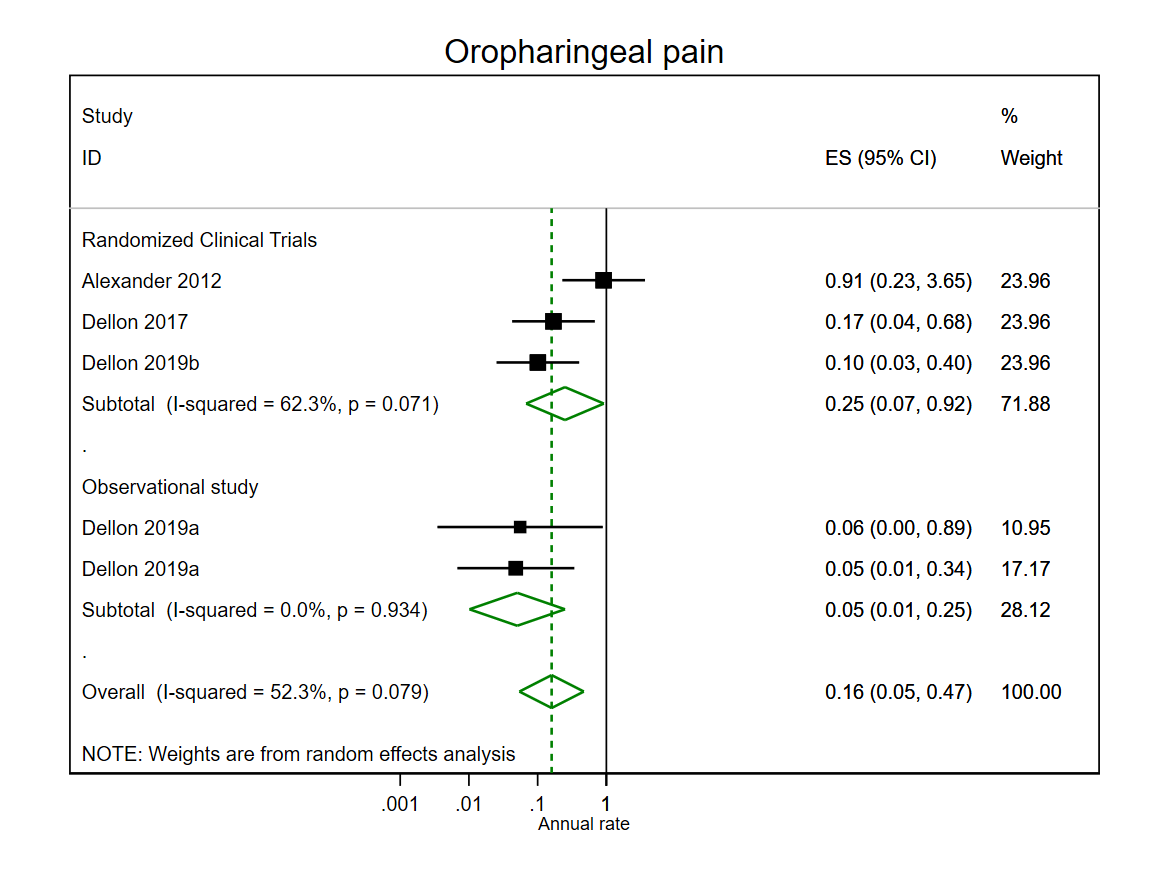


Figure E42. Forest plot of annual rate of cough (events per person-year) in patients treated with TCS


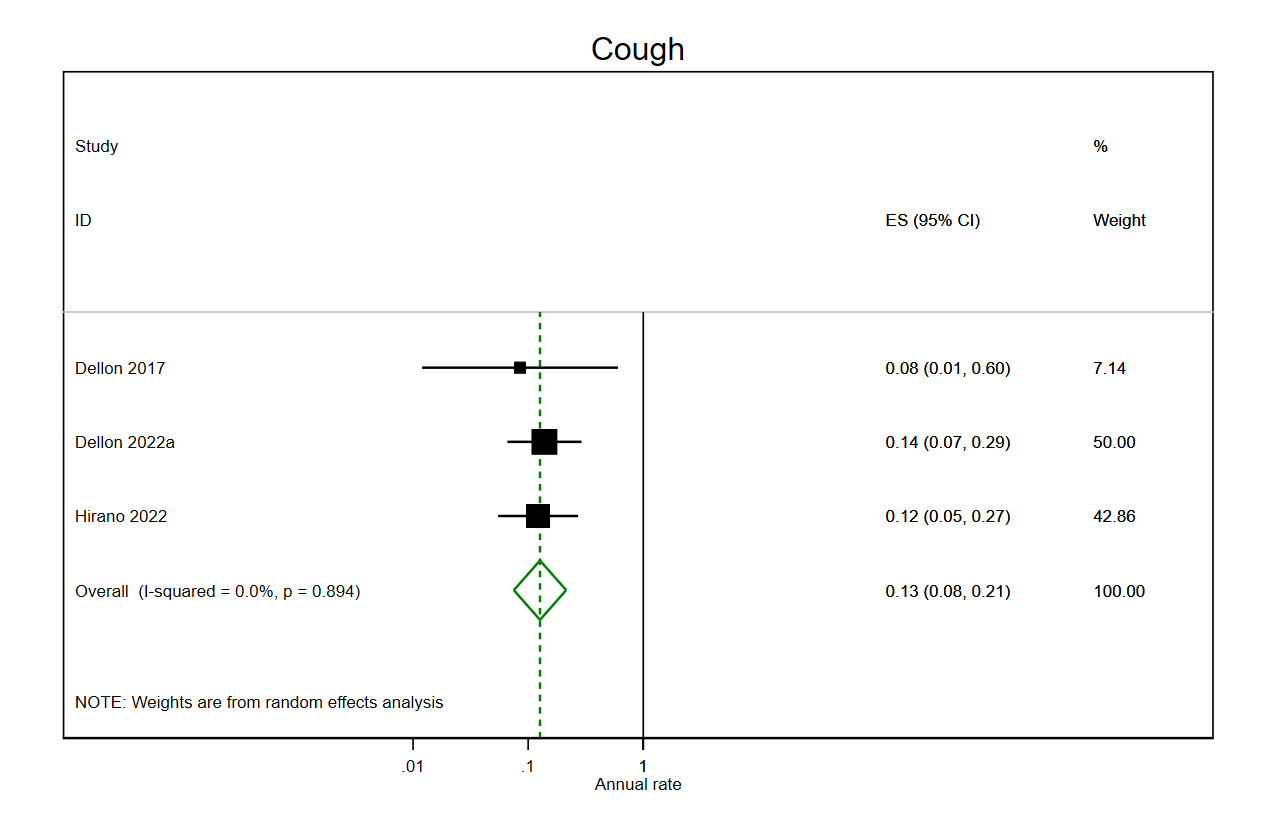


Figure E43. Forest plot of annual rate of dyspnea (events per person-year) in patients treated with TCS


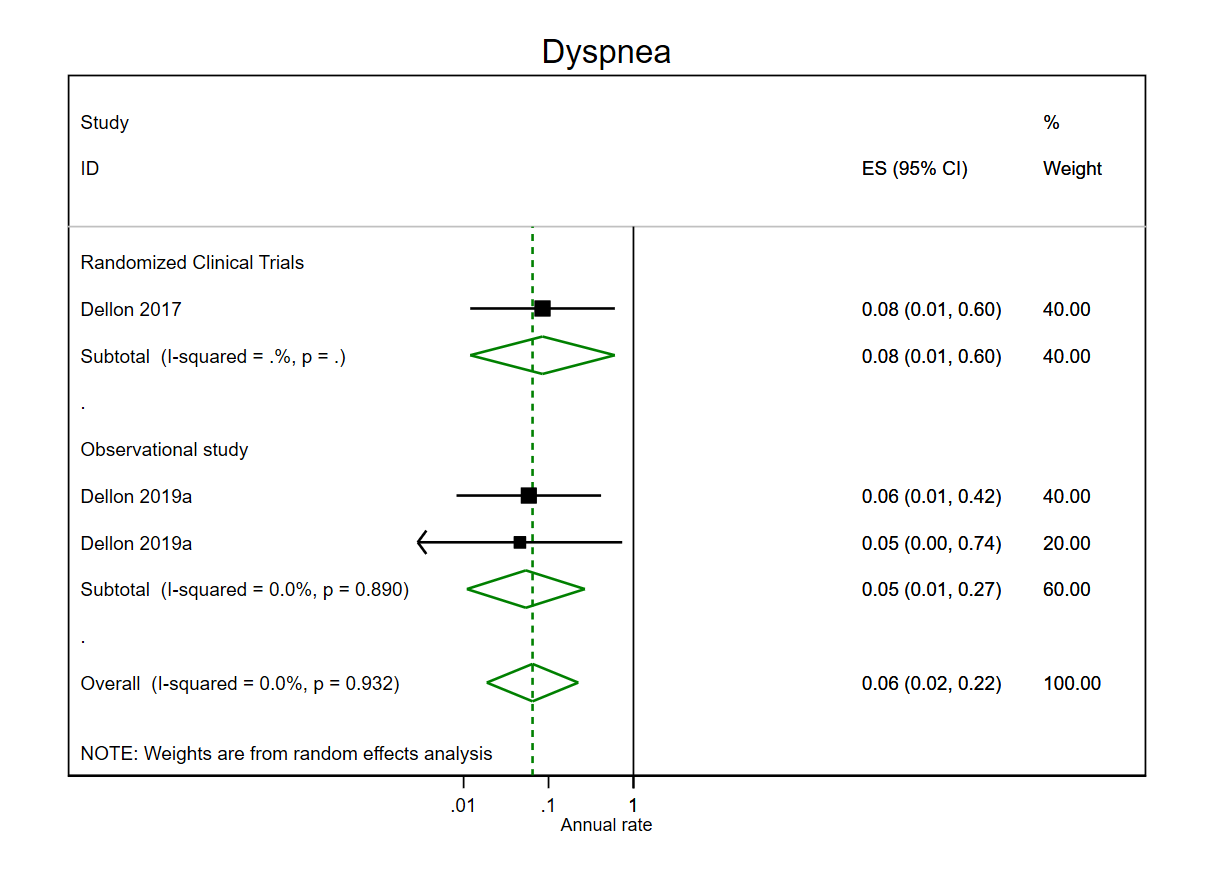


Figure E44. Forest plot of annual rate of skin disorders (events per person-year) in patients treated with TCS


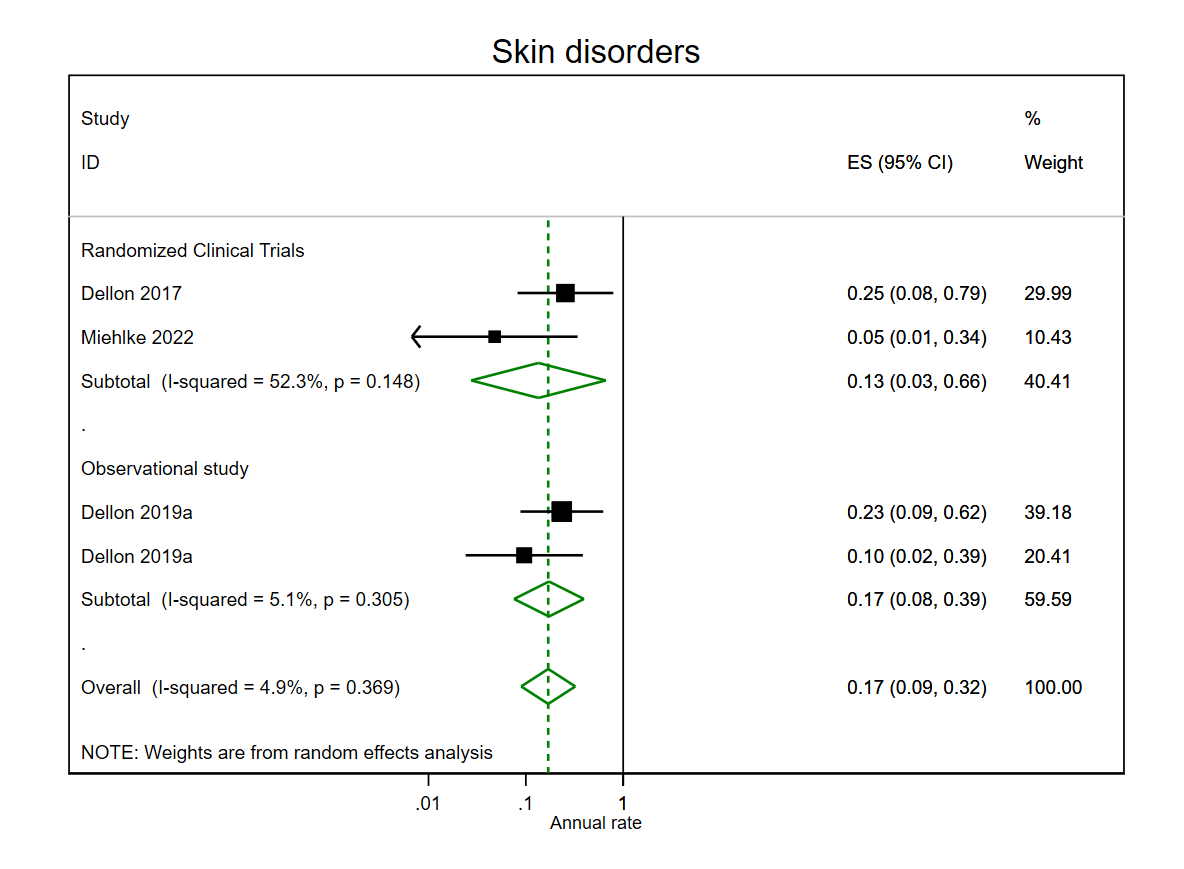


Figure E45. Forest plot of annual rate of acne (events per person-year) in patients treated with TCS


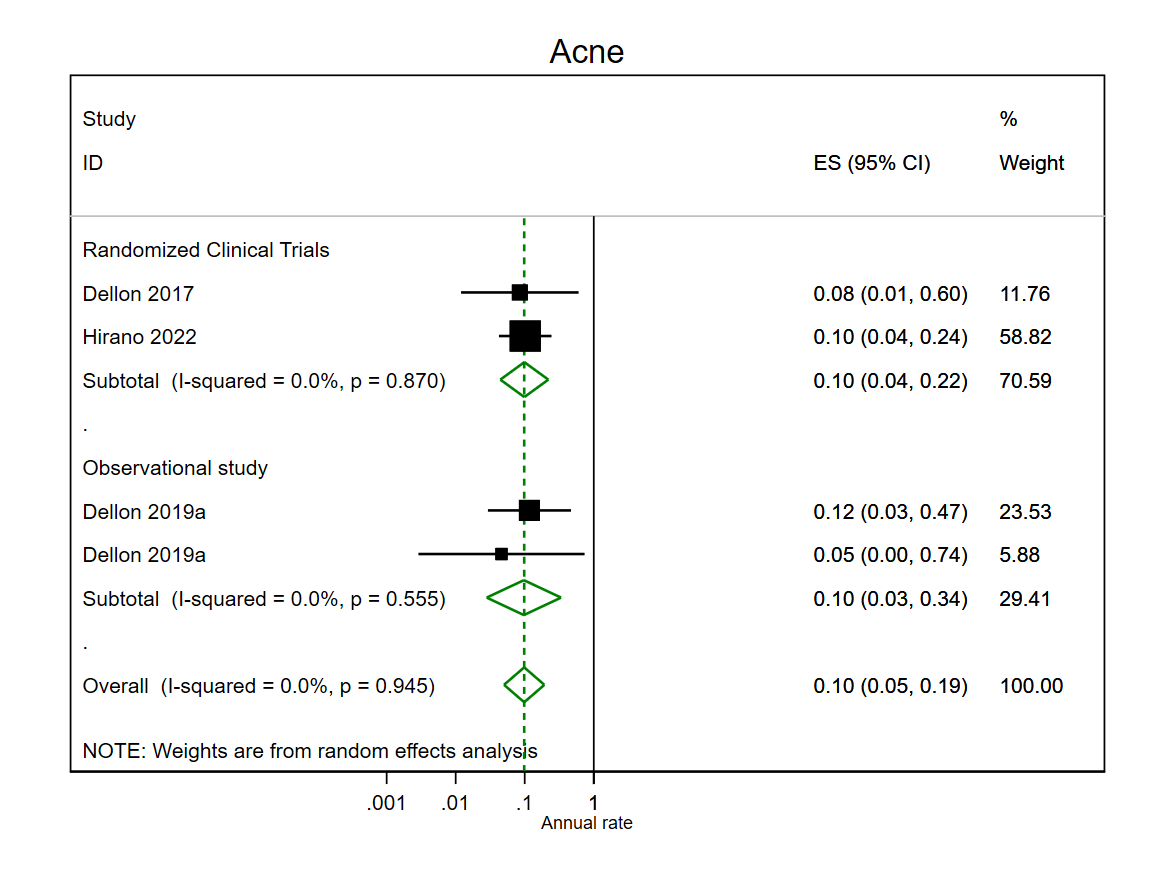


Figure E46. Forest plot of annual rate of contact dermatitis or hives (events per person-year) in patients treated with TCS


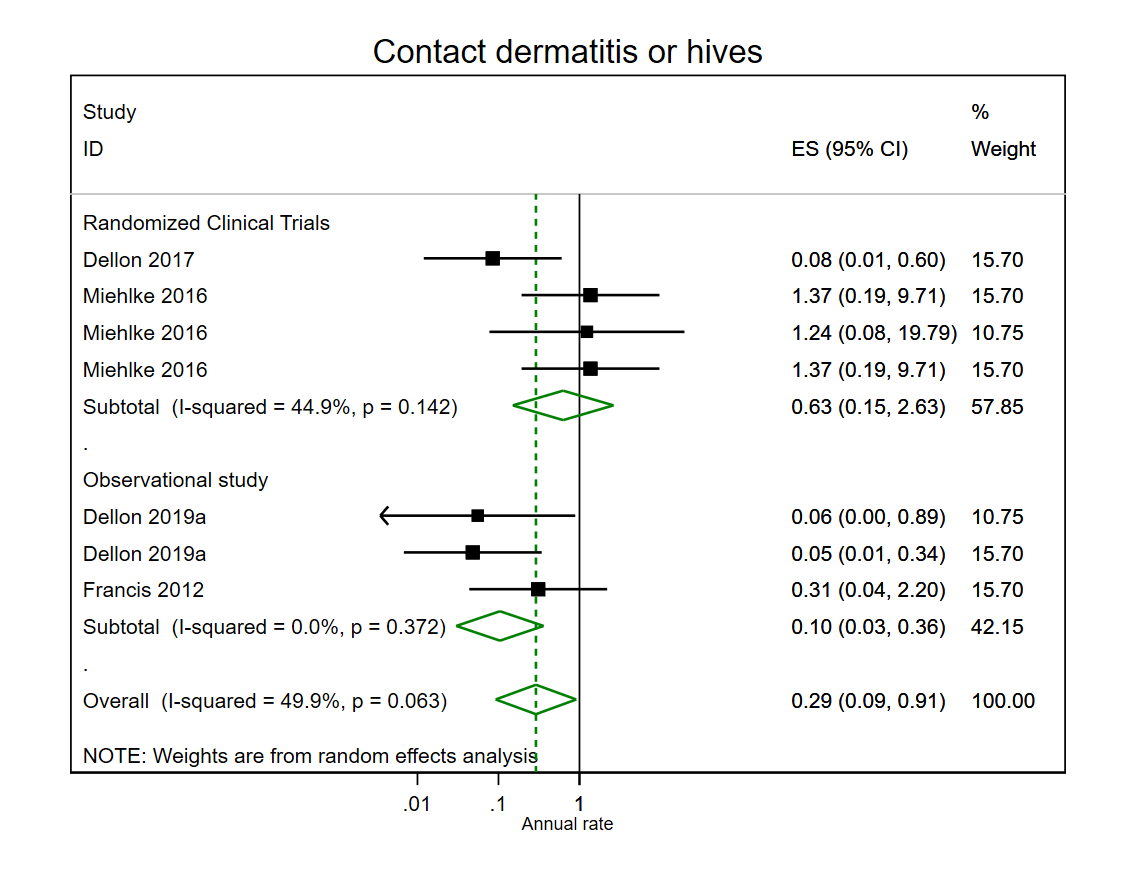


Figure E47. Forest plot of annual rate of other adverse events (events per person-year) in patients treated with TCS


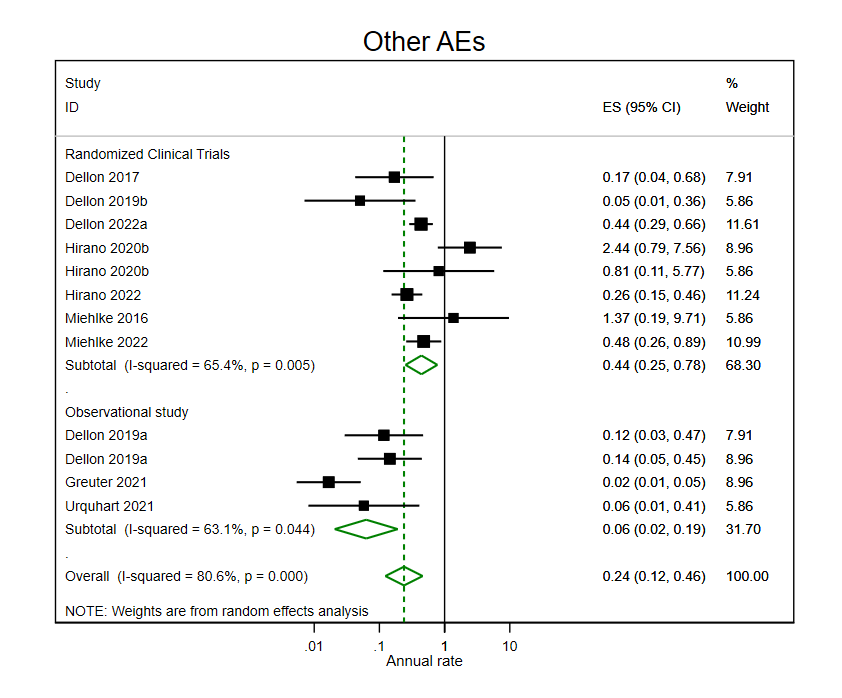


Figure E48. Forest plot of annual rate of chest pain (events per person-year) in patients treated with TCS


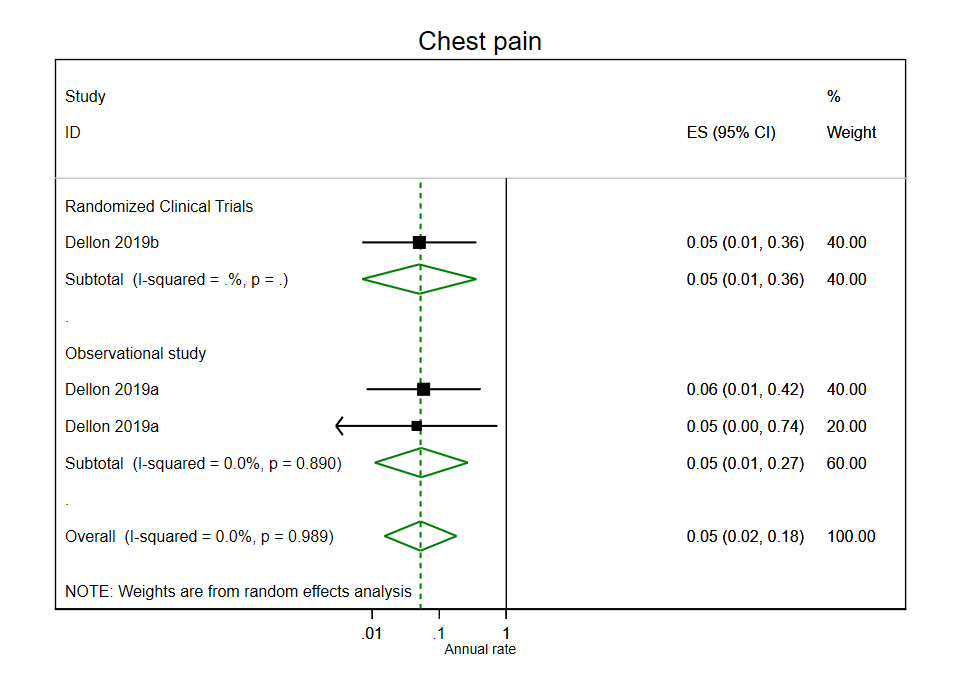


Figure E49. Forest plot of annual rate of blood cortisol decrease (events per person-year) in patients treated with TCS


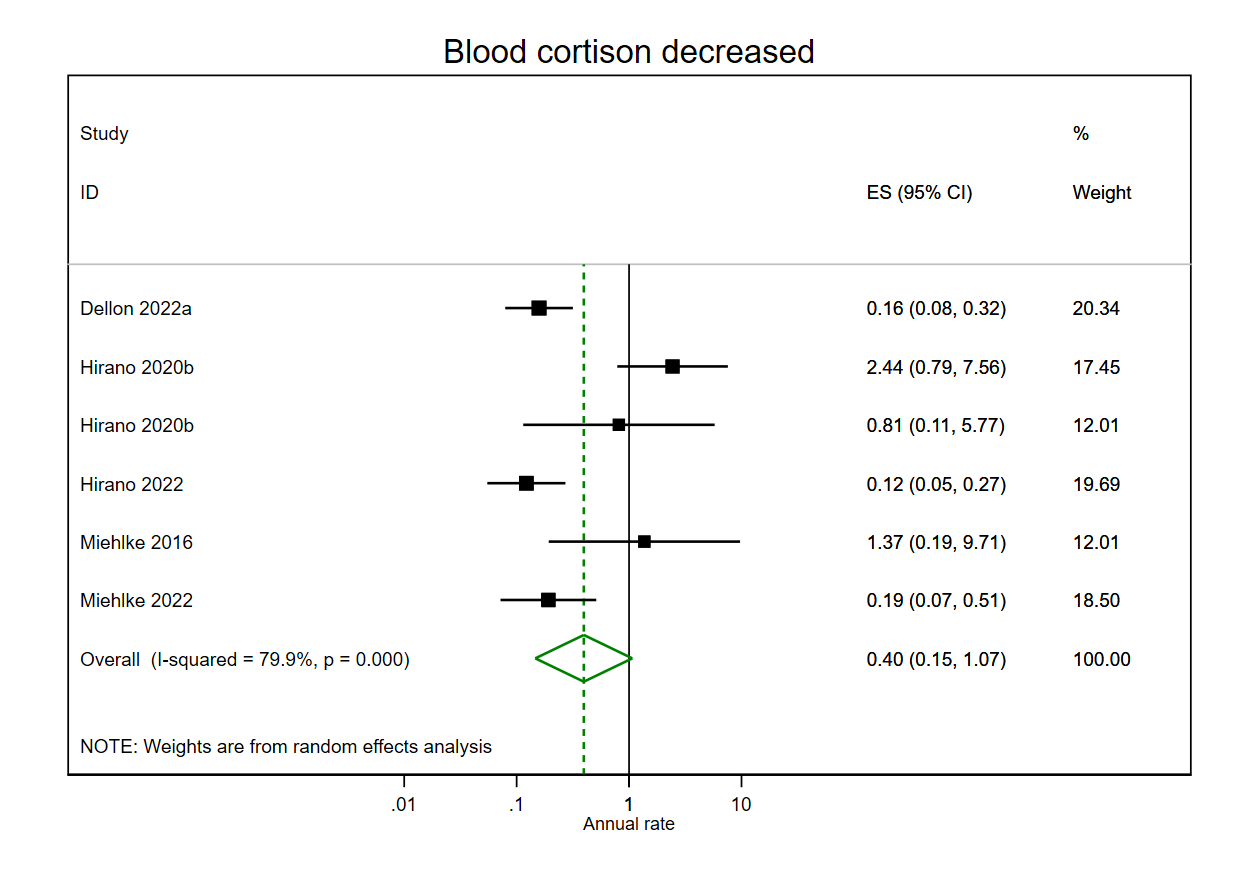


Figure E50. Forest plot of annual rate of fever (events per person-year) in patients treated with TCS


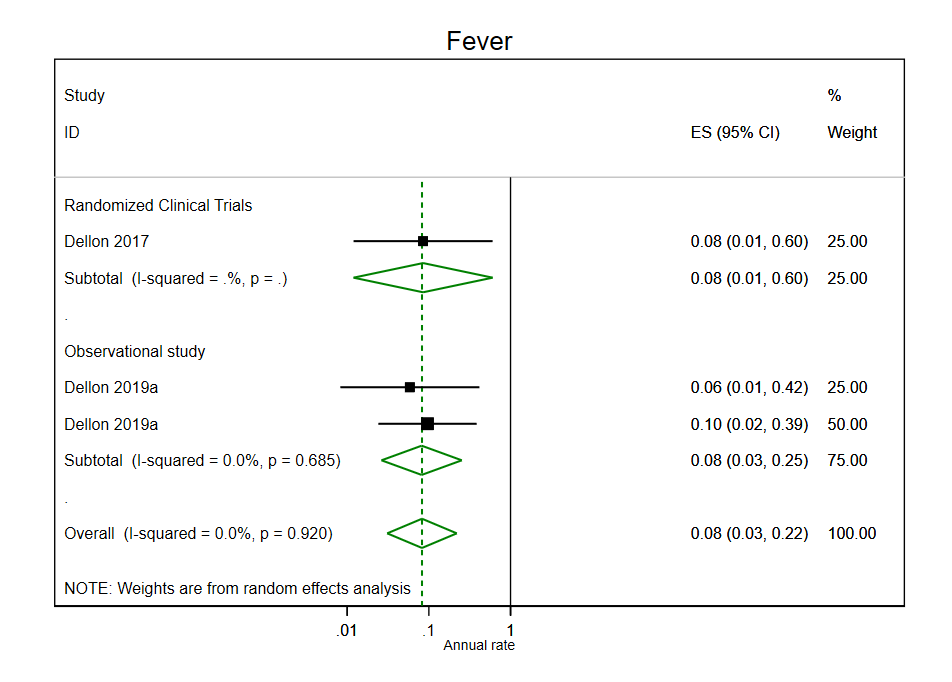


Figure E51. Forest plot of annual rate of fatigue (events per person-year) in patients treated with TCS


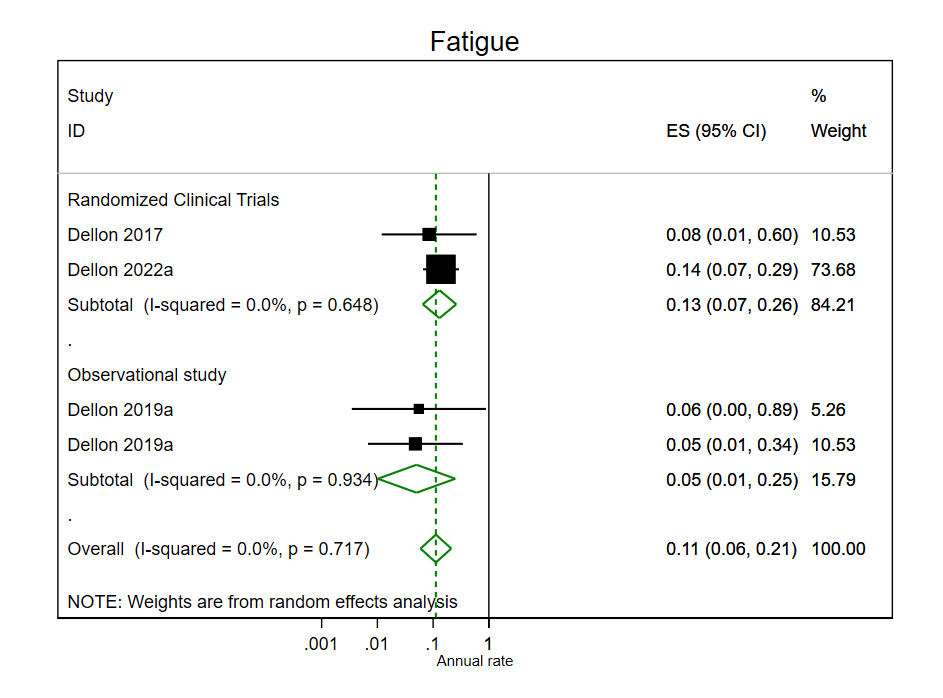


Figure E52. Forest plot of annual rate of psychiatric disorders (events per person-year) in patients treated with TCS


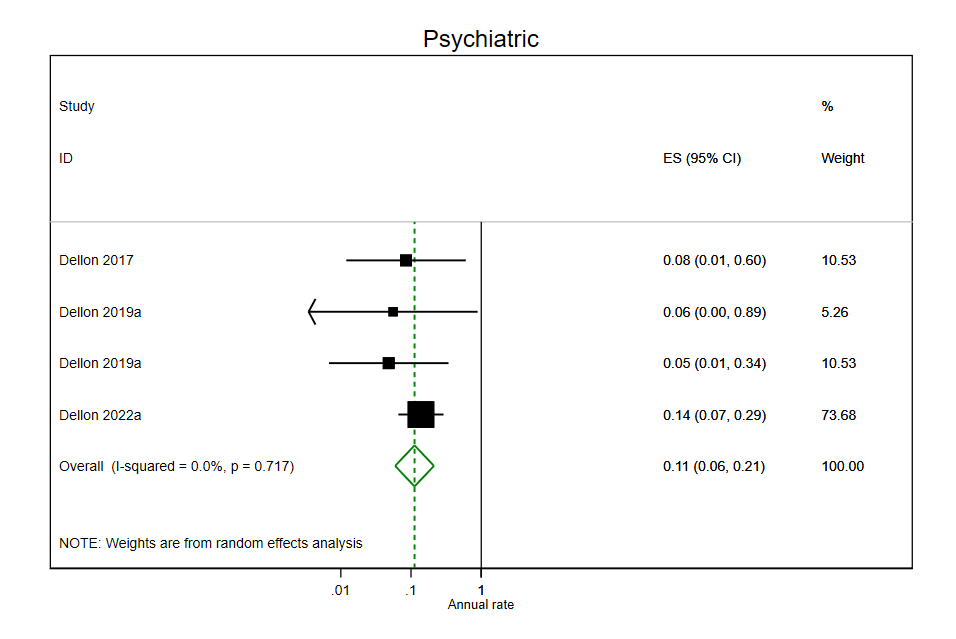


Table E1. Proportion of patients experienced adverse events stratified according to short-, mid-, or long-term follow-up

| Adverse events | Short-term FU (<12 weeks) | | | Mid-term FU (12-26 weeks) | | | Long-term FU (>26 weeks) | | |  |
| --- | --- | --- | --- | --- | --- | --- | --- | --- | --- | --- |
|  | **Studies** | **Pts** | **Estimate (95% CI)** | **Studies** | **Pts** | **Estimate (95% CI)** | **Studies** | **Pts** | **Estimate (95% CI)** |  |
| All TEAEs | 9 | 935 | 57.6% (54.6 to 60.9) | 2 | 82 | 61% (51.3 to 72.5) | 1 | 93 | 73.1% (64.6 to 82.7) | p<0.001 |
| Non serious | 9 | 935 | 55.5% (52.4 to 58.8) | 2 | 82 | 59.8% (50 to 71.4) | 1 | 93 | 71% (62.3 to 80.8) | p<0.001 |
| Serious | 9 | 935 | 2.1% (1.4 to 3.3) | 2 | 82 | 1.2% (0.2 to 8.6) | 1 | 93 | 2.2% (0.5 to 8.5) | 0.194 |
| Leading to discontinuation | 8 | 740 | 2.4% (1.5 to 3.8) | 2 | 82 | 3.7% (1.2 to 11.1) | - | - | NA | 0.505 |
| Hospitalizations | 2 | 432 | 0.7% (0.2 to 2.1) | - | - | NA | - | - | NA | NA |
| Dilation (any time) | 3 | 59 | 13.6% (7.1 to 25.8) | 1 | 27 | 66.7% (51.1 to 87) | 1 | 32 | 39.4% (25.6 to 60.5) | p<0.001 |
| Dilation (on treatment) | 3 | 59 | 5.1% (1.7 to 15.3) | 1 | 27 | 3.7% (0.5 to 25.3) | 1 | 34 | 2.9% (0.4 to 20.3) | 0.21 |
| Infection and infestation | 2 | 232 | 15.5% (11.5 to 21) | 2 | 82 | 41.5% (32.1 to 53.6) | - | - | NA | p<0.001 |
| Oral candidiasis | 9 | 509 | 2.6% (1.5 to 4.4) | 2 | 82 | 2.4% (0.6 to 9.6) | 3 | 147 | 2% (0.7 to 6.3) | 0.241 |
| Esophageal candidiasis | 11 | 909 | 8.7% (7 to 10.7) | 4 | 149 | 3.4% (1.4 to 7.9) | 5 | 216 | 5.6% (3.2 to 9.6) | 0.012 |
| UTI | 5 | 499 | 9.6% (7.4 to 12.6) | 3 | 122 | 12.3% (7.7 to 19.7) | 1 | 33 | 21.2% (11 to 40.9) | 0.01 |
| Sinusitis | 3 | 483 | 4.3% (2.9 to 6.6) | 2 | 82 | 2.4% (0.6 to 9.6) | - | - | NA | 0.429 |
| GI disorders | 2 | 232 | 8.6% (5.7 to 13.1) | 2 | 82 | 9.8% (5.1 to 18.8) | - | - | NA | 0.756 |
| Diarrhea | 4 | 286 | 4.5% (2.7 to 7.7) | 2 | 82 | 3.7% (1.2 to 11.1) | - | - | NA | 0.73 |
| Nausea/vomiting | 4 | 502 | 8.2% (6.1 to 11) | - | - | NA | - | - | NA | NA |
| Abdominal pain/discomfort | 3 | 289 | 2.4% (1.2 to 5) | 2 | 82 | 2.4% (0.6 to 9.6) | - | - | NA | 0.993 |
| Respiratory disorders | 2 | 232 | 6.5% (4 to 10.5) | 2 | 82 | 12.2% (6.8 to 21.8) | - | - | NA | 0.102 |
| Oropharyngeal pain | 3 | 199 | 3% (1.4 to 6.6) | 2 | 82 | 1.2% (0.2 to 8.6) | - | - | NA | 0.399 |
| Cough | 3 | 483 | 2.9% (1.7 to 4.9) | - | - | NA | - | - | NA | NA |
| Dyspnea | 1 | 51 | 2% (0.3 to 13.7) | 2 | 82 | 1.2% (0.2 to 8.6) | - | - | NA | 0.735 |
| Skin disorders | 2 | 232 | 1.7% (0.7 to 4.6) | 2 | 82 | 7.3% (3.4 to 15.8) | - | - | NA | 0.022 |
| Acne | 2 | 264 | 2.3% (1 to 5) | 2 | 82 | 2.4% (0.6 to 9.6) | - | - | NA | 0.93 |
| Contact dermatitis/hives | 5 | 136 | 2.9% (1.1 to 7.7) | 2 | 82 | 1.2% (0.2 to 8.6) | - | - | NA | 0.427 |
| Others | 8 | 828 | 6.4% (4.9 to 8.3) | 3 | 127 | 4.7% (2.2 to 10.3) | 1 | 82 | 3.7% (1.2 to 11.1) | 0.112 |
| Bolus impaction | - | - | NA | - | - | NA | 1 | 82 | 3.7% (1.2 to 11.1) | NA |
| Chest pain | 1 | 129 | 0.8% (0.1 to 5.5) | 2 | 82 | 1.2% (0.2 to 8.6) | - | - | NA | 0.747 |
| BCD | 6 | 648 | 3.5% (2.4 to 5.3) | - | - | NA | - | - | NA | NA |
| Fever | 1 | 51 | 2% (0.3 to 13.7) | 2 | 82 | 3.7% (1.2 to 11.1) | - | - | NA | 0.585 |
| Fatigue | 2 | 270 | 3% (1.5 to 5.9) | 2 | 82 | 1.2% (0.2 to 8.6) | - | - | NA | 0.399 |
| Dysguesia | 1 | 181 | 1.1% (0.3 to 4.4) | - | - | NA | - | - | NA | NA |
| Psychiatric | 3 | 613 | 2.9% (1.9 to 4.6) | - | - | NA | - | - | NA | NA |

BCD: blood cortisol decrease; GI: gastrointestinal; RCT: randomized clinical trial; TEAE: treatment emerging adverse event; UTI: upper tract infections

Table E2. Subgroup analysis based on pre-specified studies characteristics

|  | HR PPI  ≤15 eos/hpf | HR TCS  ≤15 eos/hpf | HR TCS  ≤5-7 eos/hpf | HR TCS  ≤1 eos/hpf | Partial CR** TCS | SMD* Symptoms score TCS | AMD* EREFS TCS | Dilation (any time) | Dilation (on treatment) | Oral candidiasis | Esophageal candidiasis |
| --- | --- | --- | --- | --- | --- | --- | --- | --- | --- | --- | --- |
| Base case | 46.2% (37.9 to 54.6) | 63.4% (57.4 to 69.4) | 55.8% (47.0 to 64.6) | 45.1% (34.3 to 55.9) | 59.5% (48.7 to 70.2) | -1.06 (-1.32 to -0.80) | -1.64 (-2.30 to -0.98) | 0.29 (0.14 to 0.57) | 0.30 (0.14 to 0.64) | 0.12 (0.05 to 0.27) | 0.32 (0.15 to 0.69) |
| EoE definition | p=0.005 | p=0.09 | p=0.699 | p=0.057 | p=0.119 | p=0.029 | p=0.493 | p=0.720 | p=0.847 | p=0.896 | p=0.897 |
| Pre 2018 GL | 37.2% (26.2 to 48.3) | 60.9% (53.4 to 68.5) | 54.8% (44.2 to 65.3) | 40.0% (30.6 to 49.1) | 52.7% (36.3 to 69.1) | -0.83 (-1.00 to -0.65) | -1.52 (-2.21 to -0.82) | 0.31 (0.13 to 0.78) | 0.32 (0.13 to 0.77) | 0.13 (0.05 to 0.34) | 0.31 (0.12 to 0.80) |
| Post 2018 GL | 55.0% (49.2 to 60.7) | 70.8% (62.2 to 79.4) | 58.1% (44.9 to 71.4) | 68.1% (40.6 to 95.7) | 69.3% (56.4 to 82.1) | -1.59 (-2.25 to -0.93) | -2.06 (-3.44 to -0.68) | 0.23 (0.06 to 0.92) | 0.27 (0.06 to 1.15) | 0.11 (0.01 to 1.04) | 0.35 (0.07 to 1.65) |
| Country | p=0.001 | p<0.001 | p<0.001 | p<0.001 | p=0.038 | p=0.002 | p<0.001 | p=0.003 | p=0.006 | p=0.335 | p=0.061 |
| Europe | 46.7% (39.6 to 53.7) | 71.4% (61.1 to 81.7)) | 70.1% (58.4 to 81.8) | 70.9% (56.6 to 85.2) | 73.3% (58.4 to 88.1) | -1.51 (-2.01 to -1.01) | -2.00 (-3.04 to -0.97) | 0.05 (0.01 to 0.20) | 0.04 (0.01 to 0.40) | 0.41 (0.03 to 5.43) | 1.06 (0.24 to 4.64) |
| US | 32.9% (21.1 to 44.7) | 55.0% (48.8 to 61.3) | 43.4% (33.3 to 53.5) | 34.3% (27.1 to 41.5) | 56.0% (40.2 to 71.8) | -0.68 (-0.87 to -0.49) | -1.12 (-2.00 to -0.25) | 0.59 (0.32 to 1.09) | 0.62 (0.34 to 1.16) | 0.11 (0.06 to 0.21) | 0.21 (0.09 to 0.46) |
| Europe + US | - | 87.0% (78.7 to 95.4) | 71.4% (60.3 to 82.4) | 64.5% (53.2 to 75.9) | 39.5% (17.2 to 61.8) | - | -3.06 (-2.24 to -1.04) | - | - | - | - |
| Asia | 67.3 (57.3 to 76.3) | 95.1% (87.9 to 100) | - | - | - | - | - | - | - | - | - |
| TCS drugs | - | p=0.433 | p=0.654 | p<0.001 | p=0.099 | p=0.004 | p=0.107 | p=0.302 | p=0.424 | p=0.03 | p=0.015 |
| FLU | - | 68.8% (58.1 to 79.5) | 50.9% (33.9 to 67.9) | 58.9% (48.4 to 69.5) | 60.3% (39.1 to 81.4) | -0.97 (-1.38 to -0.56) | -1.04 (-2.70 to 0.63) | 0.41 (0.11 to 1.57) | 0.69 (0.23 to 2.06) | 0.05 (0.00 to 2.20) | 0.13 (0.01 to 1.90) |
| BUD | - | 57.8% (44.7 to 71.0) | 58.0% (44.2 to 71.8) | 44.3% (23.9 to 64.7) | 48.7% (36.6 to 60.8) | -0.91 (-1.28 to -0.55) | -1.99 (-2.95 to -1.02) | 0.37 (0.13 to 1.09) | 0.37 (0.13 to 1.09) | 0.22 (0.11 to 0.44) | 0.70 (0.30 to 1.63) |
| FLU/BUD | - | 64.0% (55.5 to 72.5) | 51.2% (46.4 to 56.0) | 33.0% (27.7 to 38.4) | 76.8% (53.5 to 100) | -2.51 (-3.93 to -1.10) | -1.90 (-2.22 to -1.58) | 0.40 (0.03 to 5.60) | - | 0.02 (0.00 to 0.22) | 0.07 (0.01 to 0.66) |
| MF | - | 75.0% (50.9 to 91.3) | - | - | - | -0.82 (-1.18 to -0.46) | - | - | - | - | - |
| TCS formulation |  | p<0.001 | p<0.001 | p<0.001 | p=0.811 | p=0.069 | p=0.268 | p<0.001 | p<0.001 |  | p=0.005 |
| ODT (Jorveza®) |  | 85.5% (79.3 to 91.6) | 78.0% (69.5 to 86.6) | 71.8% (63.3 to 80.2) | 60.3% (40.6 to 79.9) | -1.89 (-2.98 to -0.81) | -1.23 (-2.53 to 0.08) | 0.02 (0.01 to 0.04) | 0.02 (0.01 to 0.04) | - | 1.10 (0.73 to 1.66) |
| Other |  | 59.4% (53.0 to 65.7) | 50.4% (41.1 to 59.7) | 32.1% (25.1 to 39.0) | 57.3% (43.3 to 71.3) | -0.87 (-1.04 to -0.69) | -2.02 (-2.53 to -1.52) | 0.48 (0.24 to 0.96) | 0.63 (0.32 to 1.22) |  | 0.29 (0.13 to 0.68) |
| Study quality | p=0.240 | p=0.359 | p=0.421 | p=0.162 | p=0.535 | p=0.091 | p=0.003 | p=0.112 | p=0.216 | p=0.941 | p=0.639 |
| Low quality | 49.2% (42.7 to 55.7) | 60.6% (51.8 to 69.4) | 50.8% (33.0 to 68.6) | 37.3% (25.6 to 48.9) | 63.2% (47.6 to 78.8) | -0.88 (-1.09 to -0.67) | -0.62 (-1.59 to 0.35) | 0.47 (0.21 to 1.03) | 0.46 (0.18 to 1.18) | 0.13 (0.02 to 0.80) | 0.43 (0.12 to 1.53) |
| High quality | 39.7% (25.3 to 54.1) | 66.3% (58.0 to 74.6) | 59.0% (50.0 to 68.2) | 50.5% (36.0 to 64.3) | 53.1% (40.2 to 72.0) | -1.40 (-1.96 to -0.83) | -2.45 (-3.14 to -1.77) | 0.14 (0.04 to 0.50) | 0.17 (0.05 to 0.63) | 0.12 (0.04 to 0.33) | 0.29 (0.10 to 0.80) |
| Sample size | p=0.314 | p=0.081 | p=0.029 | p=0.014 | p=0.197 | p=0.356 | p=0.263 | p=0.288 | p=0.213 | p=0.785 | p=0.304 |
| >50 patients | 51.0% (36.7 to 65.4) | 70.8% (61.8 to 79.8) | 68.1% (57.8 to 78.4) | 63.1% (43.9 to 82.3) | 50.2% (36.1 to 64.4) | -0.85 (-1.39 to -0.32) | -2.04 (-2.74 to -1.35) | 0.15 (0.03 to 0.63) | 0.07 (0.01 to 2.46) | 0.12 (0.05 to 0.29) | 0.18 (0.06 to 0.53) |
| <50 patients | 42.7 (35.2 to 50.2) | 59.9% (51.6 to 68.2) | 50.0 (37.3 to 62.6) | 36.4% (27.4 to 45.4) | 63.6 (49.0 to 78.3) | -1.14 (-1.44 to -0.83) | -1.27 (-2.42 to -0.11) | 0.38 (0.17 to 0.82) | 0.43 (0.20 to 0.93) | 0.15 (0.04 to 0.58) | 0.42 (0.13 to 1.42) |

AMD: absolute mean difference; BUD: budesonide; CR: clinical response; FLU: fluticasone; GL: guidelines; HR: histologic response; MF: mometasone furoate; ODT: oral disintegrated tablets; PPI: proton pump inhibitors; SMD: standardized mean difference; TCS: topical corticosteroids

* Change from baseline; ** EREFS≤ 2 or documented global improvement in EREFS compared with baseline

Table E3. Meta-regression analysis based on pre-specified studies characteristics; data are reported as mean effect and fraction of heterogeneity explained by each covariate (in parenthesis)

| Meta-regression | HR PPI  ≤15 eos/hpf | HR TCS  ≤15 eos/hpf | HR TCS  ≤5-7 eos/hpf | HR TCS  ≤1 eos/hpf | Partial CR** TCS | SMD* Symptoms score TCS | AMD* EREFS TCS | Dilation (any time) | Dilation (on treatment) | Oral candidiasis | Esophageal candidiasis |
| --- | --- | --- | --- | --- | --- | --- | --- | --- | --- | --- | --- |
| Heterogeneity (base case) | 97.4% | 91.7% | 96.1% | 96.2% | 94.3% | 89.2% | 97.2% | 95.9% | 92.8% | 65.6% | 91.9% |
| Age at enrollment | 1.03 (23.3%) | 1.02 (22.8%) | 1.03 (12.8%) | 1.02 (<1%) | 1.01 (<1%) | -0.04 (4.9%) | -0.07 (4.5%) | 0.96 (<1%) | 0.95 (<1%) | 1.01 (<1%) | 1.04 (<1%) |
| % male | 4.2 (8.7%) | 1.40 (<1%) | 2.26 (11.9%) | 4.84 (22.5%) | 1.42 (<1%) | 0.33 (<1%) | -3.25 (<1%) | 0.01 (13.6%) | 0.01 (12.3%) | 8.16 (12.7%) | 8.65 (25.5%) |
| Symptoms duration | - | 0.99 (<1%) | 1.05 (21.9%) | - | - | 0.04 (<1%) | -0.07 (<1%) | - | - | - | 1.22 (2.4%) |
| Clinical symptoms |  |  |  |  |  |  |  |  |  |  |  |
| Food impaction |  | 1.01 (<1%) | - | - | - | - | -0.62 (<1%) | - | - | - | - |
| Dysphagia | 0.64 (5.65%) | 0.67 (<1%) | - | 16.2 (8.6%) | - | -1.38 (<1%) | -7.32 (<1%) | - | - | - | - |
| Abdominal pain | - | 0.50 (<1%) | - | - | - | - | - | - | - | - | - |
| Heartburn | 0.54 (<1%) | 1.01 (<1%) | - | - | - | - | - | - | - | - | - |
| Endoscopic exitus |  |  |  |  |  |  |  |  |  |  |  |
| % edema | - | 1.58 (5.1%) | - | - | - | - | -2.65 (29.4%) | - | - | - | - |
| % rings | 0.97 (<1%) | 1.27 (<1%) | 0.60 (2.0%) | - | - | - | 0.42 (<1%) | - | - | - | - |
| % exudates | 0.78 (<1%) | 1.13 (<1%) | - | - | - | -1.15 (95.4%) | -4.13 (55.2%) | - | - | - | - |
| % furrows | 1.69 (10.2%) | 1.10 (<1%) | 0.97 (<1%) | - | - | 0.24 (<1%) | -3.15 (54.8%) | - | - | - | - |
| % strictures | 0.16 (40.0%) | 0.64 (7.2%) | - | - | - | - | 0.24 (<1%) | - | - | - | - |
| Mean EREFS | 0.77 (58.7%) | 0.99 (<1%) | 0.98 (<1%) | 0.94 (9.8%) | 1.08 (<1%) | -0.14 (4.2%) | -0.44 (16.1%) | - | - | - | 1.47 (6.0%) |
| Comorbidities |  |  |  |  |  |  |  |  |  |  |  |
| % atopic history | 1.88 (4.3%) | 3.38 (45.1%) | 1.70 (5.2%) | - | 0.44 (9.7%) | -2.07 (<1%) | -2.99 (2.2%) | - | - | - | - |
| % asthma | 0.51 (5.6%) | 0.80 (<1%) | 0.80 (<1%) | - | - | -2.95 (<1%) | - | - | - | - | - |
| % rhinitis | 0.66 (<1%) | 0.87 (<1%) | - | - | - | -2.62 (<1%) | - | - | - | - | - |
| % food allergies | 0.39 (35.2%) | 0.38 (43.1%) | - | - | - | 7.36 (23.3%) | - | - | - | - | - |
| % dermatitis | 2.90 (<1%) | - | - | - | - | - | - | - | - | - | - |
| Dose TCS (mg/day) | - | 1.03 (5.3%) | 0.99 (<1%) | 0.96 (<1%) | 0.85 (19.2%) | 0.04 (<1%) | -0.21 (<1%) | 1.87 (24.1%) | 1.90 (29.2%) | 1.57 (11.1%) | 1.32 (<1%) |
| Tx duration (wks) | 0.99 (13.2%) | 1.00 (<1%) | 1.00 (<1%) | 1.01 (7.1%) | 1.00 (<1%) | -0.01 (<1%) | 0.01 (1.5%) | - |  |  | 0.02 (27.9%) |
| % concomitant PPI | - | - | - | - | 0.31 (17.7%) | -0.39 (4.2%) | -0.36 (<1%) | - | - | - |  |

AMD: absolute mean difference; BUD: budesonide; CR: clinical response; FLU: fluticasone; GL: guidelines; HR: histologic response; MF: mometasone furoate; PPI: proton pump inhibitors; SMD: standardized mean difference; TCS: topical corticosteroids

* Change from baseline; ** EREFS≤ 2 or documented global improvement in EREFS compared with baseline

# Additional Material 6

Figure E1. Model scheme used to simulate EoE evolution over the 3 years of analysis


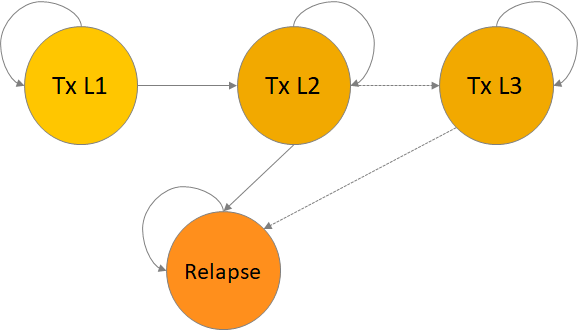


L1: first line, L2: second line; L3: third line; Tx: treatment.

Patients started PPI as first line faced other two lines of alternatives: in second line they can switch to TCS and/or FED or they can continue PPI adding TCS and/or FED; in third lines PPI was not permitted anymore and patients can add TCS to FED (or FED to TCS) or switch to FED if treated with TCS in second line (or to TCS if treated with FED in second line). Patients started all the other simulated treatments (i.e., TCS, PPI+FED, TCS+FED, or PPI+TCS) as first line, can only add TCS to FED (or FED to TCS) as second line or switch to FED if treated with TCS in first line (or to TCS if treated with FED in first line). Subsequent treatment includes a mix of PPI, TCS, FED or observation independently from the previous lines.

Table E1. Clinical and economic input used in the simulation model

| Variable | Value (95% CI if available) | Source/Assumption |
| --- | --- | --- |
| Italian resident population 18+ years |  |  |
| Year 1 | 49,222,730 | ISTAT 2024 [A] |
| Year 2 | 49,229,723 | ISTAT 2024 [A] |
| Year 3 | 49,245,102 | ISTAT 2024 [A] |
| Italian resident population 12-18 years |  |  |
| Year 1 | 3,977,396 | ISTAT 2024 [B] |
| Year 2 | 3,946,915 | ISTAT 2024 [B] |
| Year 3 | 3,891,331 | ISTAT 2024 [B] |
| EoE incidence (x 100,000) |  |  |
| 18+ years | 7 (1 to 18.3) | Arias et al. 2015 [C] |
| 12-18 years | 5.1 (1.5 to 10.9) | Arias et al. 2015 [D] |
| New EoE cases in the year |  |  |
| Year 1 | 3,648 | A x C + B x D |
| Year 2 | 3,648 | A x C + B x D |
| Year 3 | 3,648 | A x C + B x D |
| % starting 1^st^ line with PPI and/or TCS | 78.1% (68 to 88) | Based on meta-analysis of 19 studies reported information on first line treatment (see Figure E1) |
| Annual EoE cases starting treatment |  |  |
| Year 1 | 2,850 | New EoE cases in the year x 78.1% |
| Year 2 | 2,849 | New EoE cases in the year x 78.1% |
| Year 3 | 2,848 | New EoE cases in the year x 78.1% |
| First line distribution |  | Average of Desai 2005, Eluri 2020a, Kahn 2015, Kim 2018, Kim 2021, King 2010, Lin 2018, Lingblom 2014, Murray 2016, Nagarajan 2023, Okimoto 2021, Philpott 2016a, Podboy 2019, Schreiner 2023, Stern 2018, Tomomatsu 2013, Tourlamain 2020, Vermeulen 2018 |
| PPI | 53.3% |  |
| TCS | 36.4% |  |
| PPI+FED | 4.5% |  |
| TCS+FED | 1.7% |  |
| PPI+TCS | 4.1% |  |
| Distribution after PPI failure |  | Average of Desai 2005, Kim 2018, Kim 2021, Lin 2018, Nagarajan 2023, Okimoto 2021, Philpott 2016a, Tomomatsu 2013, Tourlamain 2020 |
| switch to TCS | 52.6% |  |
| switch to FED | 2.4% |  |
| switch to TCS+FED | 0.4% |  |
| add-on TCS | 25.9% |  |
| add-on FED | 14.3% |  |
| add-on TCS+FED | 4.4% |  |
| Distribution after TCS failure |  | Average of Desai 2005, Kim 2018, Kim 2021, Lin 2018, Nagarajan 2023, Okimoto 2021, Philpott 2016a, Tomomatsu 2013, Tourlamain 2020 |
| FED | 84.7% |  |
| TCS+FED | 15.3% |  |
| Distribution after FED failure |  | Average of Desai 2005, Kim 2018, Kim 2021, Lin 2018, Nagarajan 2023, Okimoto 2021, Philpott 2016a, Tomomatsu 2013, Tourlamain 2020 |
| TCS | 99.2% |  |
| TCS+FED | 0.8% |  |
| Distribution after PPI+FED failure |  | Average of Desai 2005, Kim 2018, Kim 2021, Lin 2018, Nagarajan 2023, Okimoto 2021, Philpott 2016a, Tomomatsu 2013, Tourlamain 2020 |
| TCS | 94.9% |  |
| FED | 4.3% |  |
| TCS+FED | 0.8% |  |
| Distribution after TCS+FED failure |  | Average of Desai 2005, Kim 2018, Kim 2021, Lin 2018, Nagarajan 2023, Okimoto 2021, Philpott 2016a, Tomomatsu 2013, Tourlamain 2020 |
| TCS | 95.6% |  |
| FED | 4.4% |  |
| Distribution after PPI+TCS failure |  | Average of Desai 2005, Kim 2018, Kim 2021, Lin 2018, Nagarajan 2023, Okimoto 2021, Philpott 2016a, Tomomatsu 2013, Tourlamain 2020 |
| TCS | 94.9% |  |
| FED | 4.3% |  |
| TCS+FED | 0.8% |  |
| Response assessment FU |  |  |
| PPI | 2 months | Based on average PPI trial duration |
| TCS | 6 months | Average TCS duration before gastroscopy |
| FED | 3 months | Average FED duration (4-6 elimination diet) |
| Response |  |  |
| PPI |  | Table E1 in the main paper (HR, partial/complete CR or ER) |
| TCS |  | Table E1 in the main paper (HR, partial/complete CR or ER) |
| FED |  | HR assumed equal to PPI, CR and ER assumed equal to the average between response to PPI and TCS [Laserna-Mendieta 2023] |
| PPI+FED |  | Estimated by applying RR_PPI+FED vs. PPI_=1.128 [Philpott 2016a] |
| TCS+FED |  | Estimated by applying OR_TCS+FED vs. monotherapy_=0.729 to the average response to PPI and TCS [from Schreiner 2022] |
| PPI+TCS |  | Estimated by applying OR_PPI+TCS vs. monotherapy_=1.419 to the average response to PPI and TCS [from Schreiner 2022] |
| Esophageal dilation |  |  |
| PPI (cases x 100 PY) | 16 (7 to 39) | Based on meta-analysis of 19 studies reported information on first line treatment (see Figure E1) |
| TCS (cases x 100 PY) | 30 (14 to 64) | Table E2 in the main paper (dilation rate on treatment) |
| FED (cases x 100 PY) | 19.6 (8.9 to 43.8) | Assumed equal to dilation rate without drug treatment, estimated by applying OR_NT vs. monotherapy_=0.850 to the average rate with PPI and TCS [from Schreiner 2022] |
| Relapse rate |  |  |
| PPI (cases x 100 PY) | 14.6 (9.2 to 23) | Table E1 in the main paper (relapse rate with PPI) |
| TCS (cases x 100 PY) | 34.8 (22.3 to 54.4) | Table E1 in the main paper (relapse rate to TCS) |
| FED (cases x 100 PY) | 22.4 (17.8 to 28.8) | Assumed the average between PPI and TCS [Laserna-Mendieta 2023] |
| Monthly costs treatment |  |  |
| PPI | € 10.52 | 44% esomeprazole (20 mg die, pack 28 tabs 40 mg € 10.92), 44% omeprazole (20 mg die, pack 28 tans € 8.45), 4% pantoprazole (20 mg die, pack 28 tabs 40 mg € 10.94), 4% lansoprazole (30 mg die, pack 28 tabs 30 mg € 10.75), 4% rabeprazole (20 mg die, pack 28 tabs 20 mg € 8.84) |
| TCS | € 27.64 | 68.6% fluticasone (880 mcg die, 120 puff 250 mcg € 14.39), 31.4% budesonide (1,5 g die, 200 puff 200 mcg € 27.95) |
| FED | € 22.00 | One dietician’s visit every month (code 89.7) |
| Cost dilation | € 161.04 | 50% outpatient procedure without biopsy (€ 64.90, code 45.13), 35% outpatient procedure with biopsy (€ 123.55, code 45.16), 10% day-hospital (€ 295, DRG 183), 5% hospitalization (€ 959, DRG 183) + antibiotics prophylaxis (€ 7.90 amoxicillin + clavulanic acid) |
| Monthly cost relapse | € 26.90 | Based on distribution of treatments among prevalent EoE patients, with overlap: 53.2% PPI, 44.9% TCS, 30.4% FED, 10% observation (average of Chang 2021a, Leigh 2019, Lin 2018, Lucendo 2013, Maradey-Romero 2015, Menard-Katcher 2013, Nicodème 2013, Ocampo 2023, Podboy 2019, Safroneeva 2018, Safroneeva 2020, Savarino 2020, Schreiner 2022, Vermeulen 2018) |
| Monthly cost AEs | € 65.50 | Sum of management cost for each AE |
| Oral candidiasis | € 2.48 | ER visit cost x annual rate/12 |
| Esophageal candidiasis | € 6.62 | ER visit cost x annual rate/12 |
| Other infections | € 24.28 | (4% € 1,247 [DRG 69] + 96% ER visit cost) x annual rate/12 |
| GI disorders | € 4.35 | average cost nausea/diarrhea/vomit € 153.51 [Lazzaro 2013] x annual rate/12 |
| Respiratory disorders | € 9.83 | (4% € 1,689 [DRG 100] + 96% ER visit cost) x annual rate/12 |
| Skin disorders | € 6.00 | ER visit cost x annual rate/12 |
| BCD | € 8.28 | ER visit cost x annual rate/12 |
| Fever | € 1.72 | (4% € 463.45 [Lazzaro 2013] + 96% ER visit cost) x annual rate/12 |
| Fatigue | € 1.22 | € 133.26 [Lazzaro 2013] x annual rate/12 |
| Psychiatric disorders | € 0.73 | € 79.78 [Lazzaro 2013] x annual rate/12 |

AE: adverse event; BCD: blood cortisol decrease; CI: confidence intervals; ER: emergency room; EoE: eosinophilic esophagitis; FED: food elimination diet; GI: gastrointestinal; PPI: proton pump inhibitors; PY: person years; TCS: topical corticosteroids

Figure E2. Meta-analysis on proportion of patients started PPI and/or TCS as first line of treatment


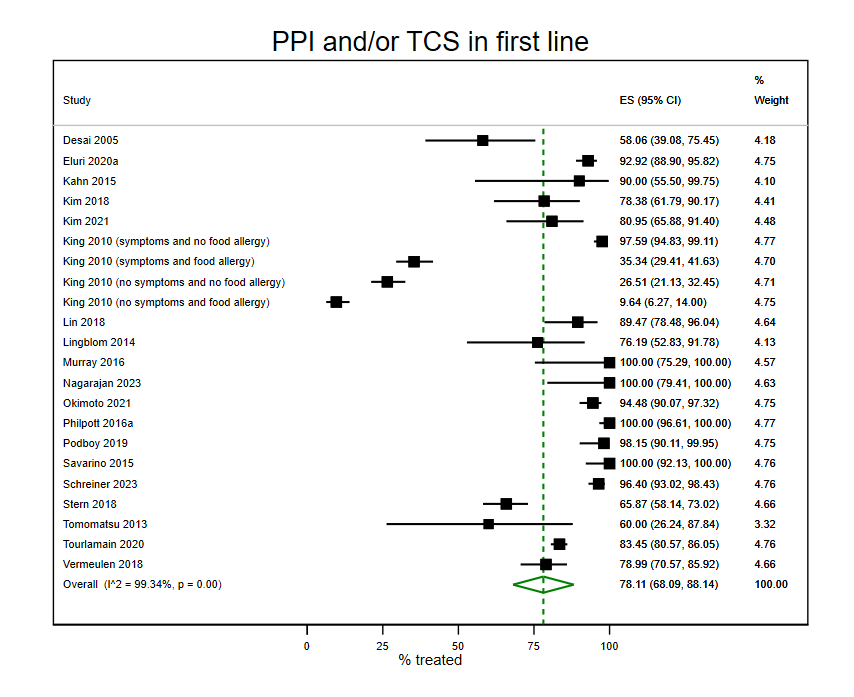


Figure E3. Meta-analysis on dilation rate (cases per PY) during treatment with PPI


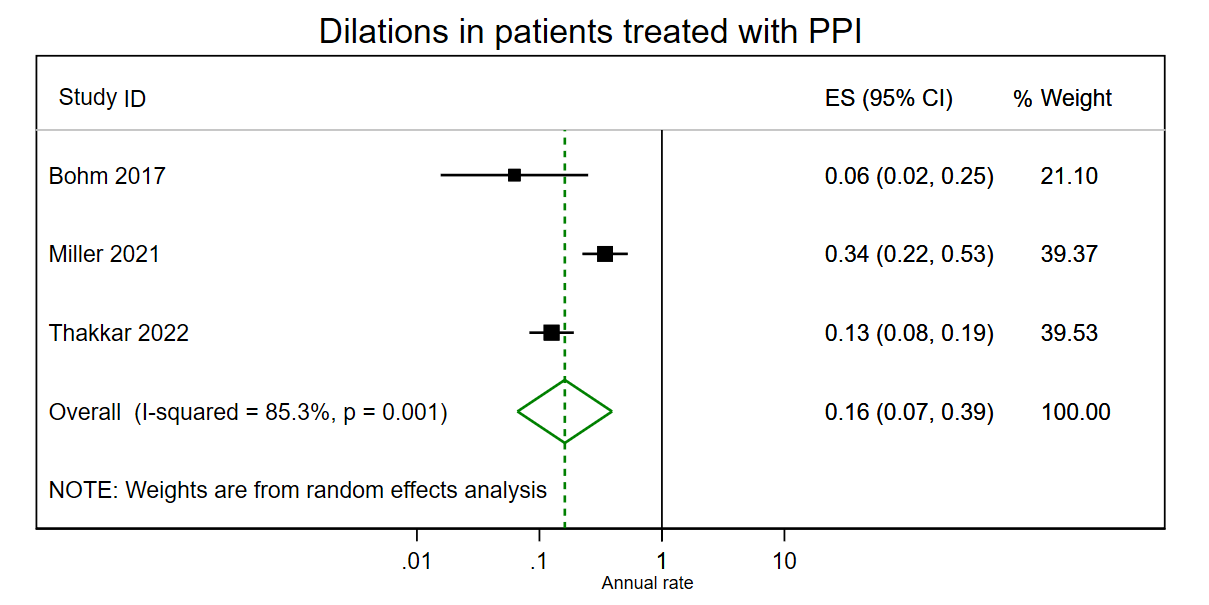


Table E2. Total and detailed mean cost per patients according to response assessment based on HR≤15 eos/hpf.

| Cost | PPI | TCS | PPI+FED | TCS+FED | PPI+TCS |
| --- | --- | --- | --- | --- | --- |
| Treatment cost | € 486.25 | € 672.57 | € 783.61 | € 1,070.87 | € 969.57 |
| Dilation cost | € 1,403.86 | € 1,693.85 | € 1,484.72 | € 1,756.99 | € 1,761.60 |
| Relapse cost | € 560.43 | € 1,310.49 | € 549.06 | € 1,651.65 | € 1,759.96 |
| AE cost | € 315.85 | € 333.86 | € 311.51 | € 304.28 | € 266.43 |
| **Total cost** | **€ 2,766.39** | **€ 4,010.77** | **€ 3,128.90** | **€ 4,783.79** | **€ 4,757.56** |

Table E3. Total and detailed mean cost per patients according to response assessment based on HR≤5-7 eos/hpf.

| Cost | PPI | TCS | PPI+FED | TCS+FED | PPI+TCS |
| --- | --- | --- | --- | --- | --- |
| Treatment cost | € 478.42 | € 637.47 | € 718.58 | € 995.21 | € 918.96 |
| Dilation cost | € 1,471.45 | € 1,691.36 | € 1,536.52 | € 1,754.44 | € 1,763.02 |
| Relapse cost | € 608.88 | € 1,211.44 | € 625.52 | € 1,570.52 | € 1,687.01 |
| AE cost | € 374.93 | € 368.13 | € 360.49 | € 335.09 | € 295.92 |
| **Total cost** | **€ 2,933.68** | **€ 3,908.40** | **€ 3,241.11** | **€ 4,655.26** | **€ 4,664.91** |

Table E4. Total and detailed mean cost per patients according to response assessment based on HR≤1 eos/hpf.

| Cost | PPI | TCS | PPI+FED | TCS+FED | PPI+TCS |
| --- | --- | --- | --- | --- | --- |
| Treatment cost | € 447.23 | € 587.97 | € 655.21 | € 903.39 | € 849.55 |
| Dilation cost | € 1,511.95 | € 1,687.75 | € 1,567.71 | € 1,751.66 | € 1,765.36 |
| Relapse cost | € 584.88 | € 1,071.92 | € 609.02 | € 1,451.05 | € 1,575.60 |
| AE cost | € 431.42 | € 416.26 | € 414.03 | € 381.28 | € 341.36 |
| **Total cost** | **€ 2,975.48** | **€ 3,763.90** | **€ 3,245.97** | **€ 4,487.38** | **€ 4,531.87** |

Table E5. Total and detailed mean cost per patients according to response assessment based on partial clinical response.

| Cost | PPI | TCS | PPI+FED | TCS+FED | PPI+TCS |
| --- | --- | --- | --- | --- | --- |
| Treatment cost | € 472.28 | € 663.41 | € 785.17 | € 1,050.66 | € 956.40 |
| Dilation cost | € 1,387.00 | € 1,686.80 | € 1,471.97 | € 1,757.10 | € 1,761.97 |
| Relapse cost | € 515.73 | € 1,261.79 | € 492.99 | € 1,622.37 | € 1,737.17 |
| AE cost | € 314.03 | € 341.86 | € 313.79 | € 315.88 | € 275.58 |
| **Total cost** | **€ 2,689.04** | **€ 3,953.86** | **€ 3,063.92** | **€ 4,746.01** | **€ 4,731.12** |

Table E6. Total and detailed mean cost per patients according to response assessment based on complete clinical response.

| Cost | PPI | TCS | PPI+FED | TCS+FED | PPI+TCS |
| --- | --- | --- | --- | --- | --- |
| Treatment cost | € 417.34 | € 537.86 | € 606.35 | € 838.54 | € 796.94 |
| Dilation cost | € 1,527.08 | € 1,685.73 | € 1,580.07 | € 1,749.88 | € 1,767.71 |
| Relapse cost | € 534.93 | € 933.74 | € 541.06 | € 1,342.11 | € 1,477.59 |
| AE cost | € 468.69 | € 466.32 | € 458.87 | € 424.23 | € 381.94 |
| **Total cost** | **€ 2,948.04** | **€ 3,623.65** | **€ 3,186.35** | **€ 4,354.76** | **€ 4,424.18** |

Table E7. Total and detailed mean cost per patients according to response assessment based on partial endoscopic response.

| Cost | PPI | TCS | PPI+FED | TCS+FED | PPI+TCS |
| --- | --- | --- | --- | --- | --- |
| Treatment cost | € 473.03 | € 689.79 | € 829.57 | € 1,113.64 | € 995.22 |
| Dilation cost | € 1,340.30 | € 1,693.05 | € 1,435.41 | € 1,758.55 | € 1,761.04 |
| Relapse cost | € 477.19 | € 1,351.92 | € 434.98 | € 1,687.57 | € 1,792.31 |
| AE cost | € 275.66 | € 316.71 | € 280.79 | € 291.02 | € 253.53 |
| **Total cost** | **€ 2,566.18** | **€ 4,051.47** | **€ 2,980.75** | **€ 4,850.78** | **€ 4,802.10** |

Table E8. Total and detailed mean cost per patients according to response assessment based on complete endoscopic response.

| Cost | PPI | TCS | PPI+FED | TCS+FED | PPI+TCS |
| --- | --- | --- | --- | --- | --- |
| Treatment cost | € 460.92 | € 633.22 | € 744.31 | € 988.57 | € 914.13 |
| Dilation cost | € 1,419.93 | € 1,681.72 | € 1,497.68 | € 1,755.63 | € 1,763.22 |
| Relapse cost | € 521.79 | € 1,166.22 | € 508.87 | € 1,547.82 | € 1,671.98 |
| AE cost | € 349.56 | € 370.87 | € 346.58 | € 344.67 | € 301.95 |
| **Total cost** | **€ 2,752.20** | **€ 3,852.03** | **€ 3,097.44** | **€ 4,636.69** | **€ 4,651.28** |
